# Reproductive developmental transcriptome analysis of *Tripidium ravennae* (Poaceae)

**DOI:** 10.1186/s12864-021-07641-y

**Published:** 2021-06-28

**Authors:** Nathan Maren, Fangzhou Zhao, Rishi Aryal, Darren Touchell, Wusheng Liu, Thomas Ranney, Hamid Ashrafi

**Affiliations:** 1 Department of Horticultural Science, North Carolina State University, Campus Box 7609, Raleigh, NC 27695-7609 USA; 2 College of Agriculture, Nanjing Agricultural University, Nanjing, 210095 China; 3 Mountain Crop Improvement Lab, Department of Horticultural Science, Mountain Horticultural Crops Research and Extension Center, North Carolina State University, 455 Research Drive, Mills River, NC 28759-3423 USA

**Keywords:** *Tripidium ravennae*, Transcriptome sequencing, Differential gene expression, Next-generation sequencing, Floral transition, Flowering, Inflorescence development, Reproduction, RNA sequencing, Seed development

## Abstract

**Background:**

*Tripidium ravennae* is a cold-hardy, diploid species in the sugarcane complex (*Poaceae* subtribe *Saccharinae*) with considerable potential as a genetic resource for developing improved bioenergy and ornamental grasses. An improved understanding of the genetic regulation of reproductive processes (e.g., floral induction, inflorescence development, and seed development) will enable future applications of precision breeding and gene editing of floral and seed development. In particular, the ability to silence reproductive processes would allow for developing seedless forms of valuable but potentially invasive plants. The objective of this research was to characterize the gene expression environment of reproductive development in *T. ravennae.*

**Results:**

During the early phases of inflorescence development, multiple key canonical floral integrators and pathways were identified. Annotations of type II subfamily of MADS-box transcription factors, in particular, were over-represented in the GO enrichment analyses and tests for differential expression (FDR *p*-value < 0.05). The differential expression of floral integrators observed in the early phases of inflorescence development diminished prior to inflorescence determinacy regulation. Differential expression analysis did not identify many unique genes at mid-inflorescence development stages, though typical biological processes involved in plant growth and development expressed abundantly. The increase in inflorescence determinacy regulatory elements and putative homeotic floral development unigenes at mid-inflorescence development coincided with the expression of multiple meiosis annotations and multicellular organism developmental processes. Analysis of seed development identified multiple unigenes involved in oxidative-reductive processes.

**Conclusion:**

Reproduction in grasses is a dynamic system involving the sequential coordination of complex gene regulatory networks and developmental processes. This research identified differentially expressed transcripts associated with floral induction, inflorescence development, and seed development in *T. ravennae*. These results provide insights into the molecular regulation of reproductive development and provide a foundation for future investigations and analyses, including genome annotation, functional genomics characterization, gene family evolutionary studies, comparative genomics, and precision breeding.

**Supplementary Information:**

The online version contains supplementary material available at 10.1186/s12864-021-07641-y.

## Background

The need for and importance of alternative energy sources becomes increasingly essential as global energy demands grow with concomitant fossil fuel reserve depletion. Bioenergy crops suitable as fuel for heat, electric power generation, and processing into cellulosic ethanol continue to attract attention as alternative fuel sources. Members of the grass family *Poaceae* subtribe *Saccharinae*, also known as the sugarcane complex, have gained attention for their broad adaptability, pest resistance, high biomass yields, and potential for perennially sequestering large amounts of carbon with few inputs on marginal lands [1–5].

The *Saccharinae* are diverse, spanning numerous genera include *Erianthus, Miscanthus*, *Saccharum,* and *Tripidium* [6–8]. Though previously placed in the genus *Saccharum* L. [9], then *Erianthus* (L.) P. Beauv [10]., Valdés and Scholz [7] transferred the four members of Old-World *Erianthus* into *Tripidium* based on both molecular and morphological features. The genus *Tripidium* currently circumscribes all the Old-World members of *Erianthus* sect. *Ripidium* resolving the debate over *Tripidium* spp. taxonomy since the partial treatment of the group by Valdés and Scholz [6, 7]. *Tripidium ravennae* (L.) H. Scholz (syn. *E. ravennae*, *S. ravennae* (Ravenna grass) is diploid (2*n* = 2*x* = 20) and cold-hardy to USDA Zone 5b [5]. Ravenna grass has a broad native range spanning Eastern Europe, North Africa, and Southwestern Asia, but has naturalized in several locales of the new world [10–12]. The use of *T. ravennae* as a landscape ornamental or on marginal lands and riparian areas for erosion control purposes [13–15] created an opportunity for escape, and it is now considered weedy in some areas [16, 17].

Conventional breeding is well suited for improving complex traits such as yield and cold-hardiness. Maren et al. [3] recently reported on new interspecific *Tripidium* hybrids with significantly higher biomass yields than *Miscanthus ×giganteus* and cold hardiness to USDA Zone 6b/7a. However, plant biotechnology has considerable potential to augment conventional breeding and make value-added improvements in elite clonally propagated cultivars without compromising other genetically complex and desirable traits. For example, silencing key reproductive processes could reduce reseeding and invasive potential of valuable bioenergy grass clones.

The role of plant reproduction in crop production and yield encouraged extensive research into the genetics of flowering among agricultural cereals such as wheat (*Triticum* spp.) [18–20] and barley (*Hordeum vulgare*) [21–23]. However, current information on the genetics and translational genomics of reproductive development in perennial grass species of the *Panicoideae* (including *Tripidium* spp.), *Aristidoideae*, *Chloridoideae*, *Micrairoideae*, *Arundinoideae*, and *Danthonioideae* (PACMAD) clade of the *Poaceae* is limited [24]. A foundation for future application of precision breeding and gene editing depends on a detailed understanding of the reproductive process’ genetic regulation. Transcriptomic and RNA-sequencing analyses allow for examining gene expression regardless of prior sequencing context and enable the identification of candidate genes for modification [25, 26]. The processes of floral initiation [27], inflorescence development [28, 29], and seed development [30, 31] involve multifaceted changes in gene expression. An extensive literature search identified minimal genetic information or gene expression analysis of *T. ravennae*. The aim of this study was to characterize the genetic control and differentially expressed transcripts in reproductive development pathways of the diploid perennial bioenergy grass *T. ravennae*.

## Results

### Transcriptome assembly and functional annotation

**Fig. 1:**
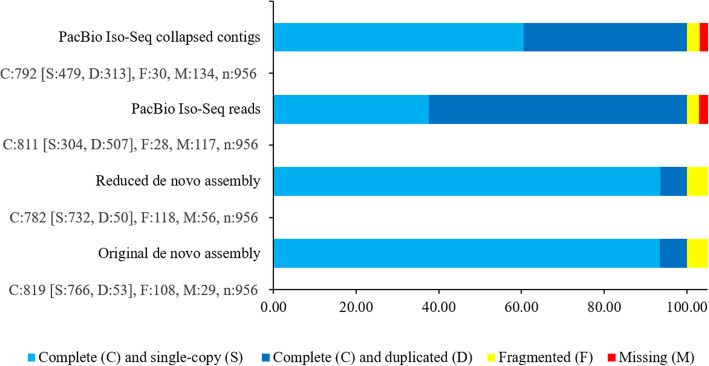
*Tripidium ravennae* transcriptome quality assessment with BUSCO analysis. C, S, D, F, and M represent complete, single, duplicate, fragmented, and missing BUSCOs, respectively

**Fig. 2:**
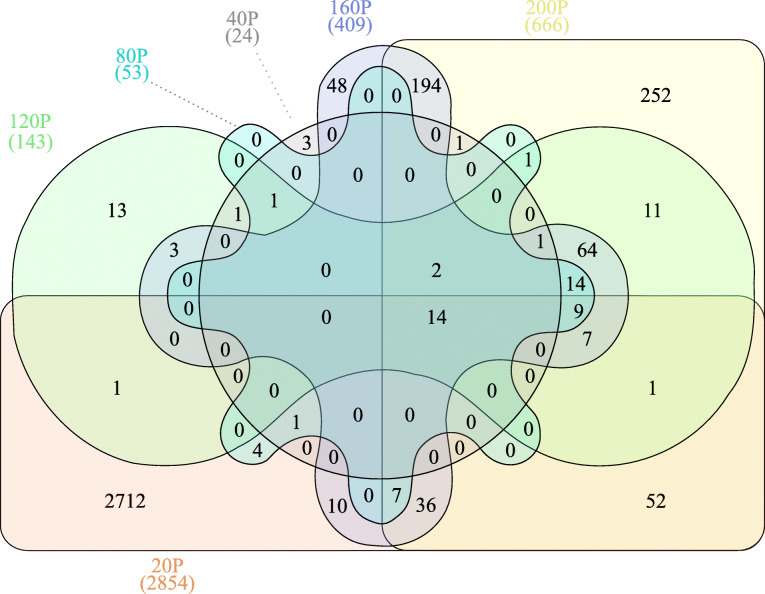
Venn diagram of differentially expressed unigenes in inflorescence development. Numbers represent unigenes with an absolute and minimum two-fold change in expression relative to controls (FDR *p*-value < 0.05).  Inflorescence meristem sampling at ~ 20 cm from culm base (20P).  Inflorescence meristem sampling at ~ 40 cm from culm base (40P).  Inflorescence meristem sampling at ~ 80 cm from culm base (80P).  Inflorescence meristem sampling at ~ 120 cm from culm base (120P).  Inflorescence meristem sampling at ~ 160 cm from culm base (160P).  Inflorescence meristem sampling at ~ 200 cm from culm base (200P)

**Fig. 3:**
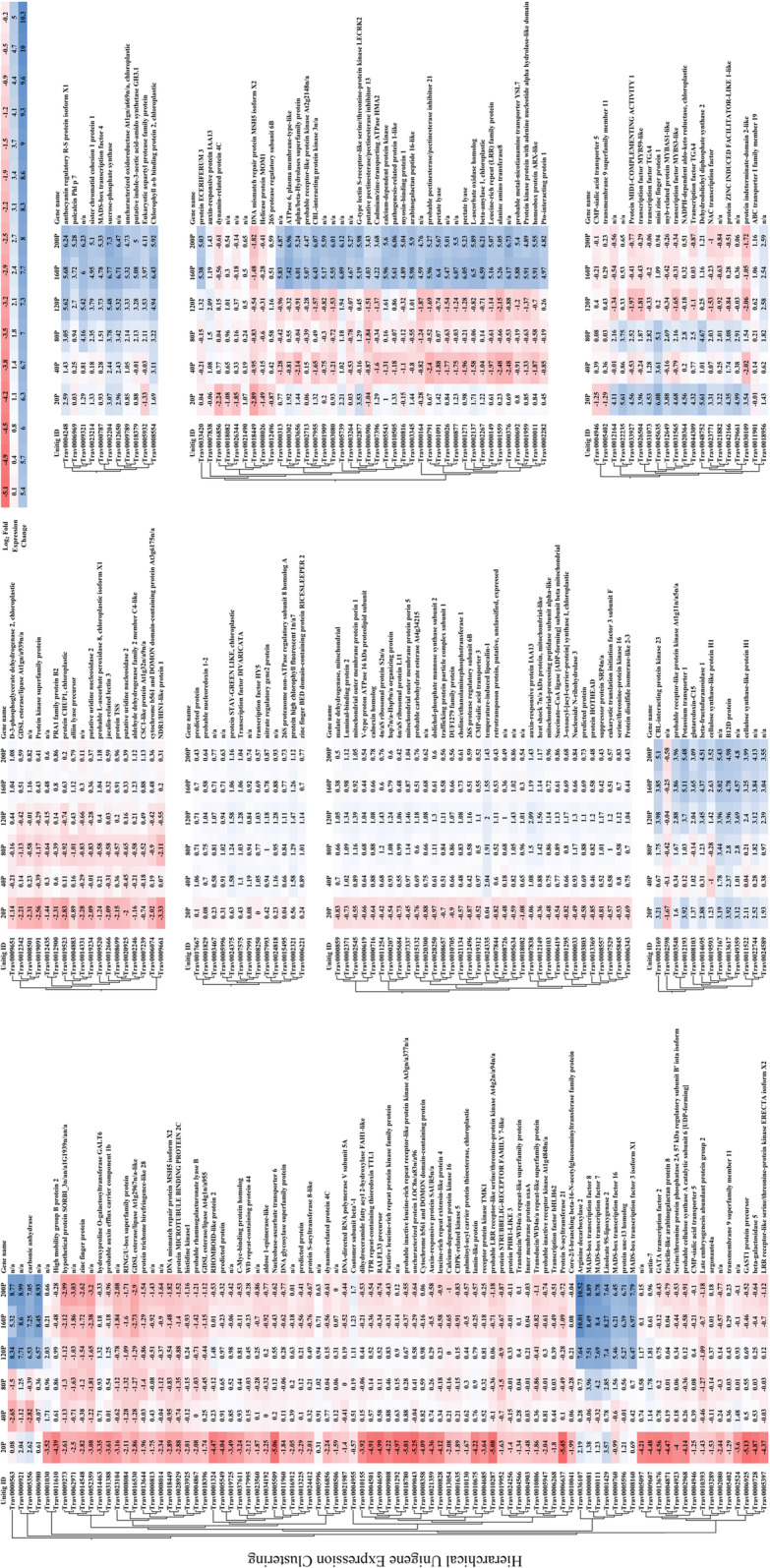
Heat map of hierarchically clustered inflorescence unigenes. Absolute values of transcript per million normalized gene expression ranged from a minimum of two-fold to > 3000-fold change in expression relative to controls (FDR *p* < 0.05). Columns represent log_2_-fold expression values of all biological replicates in developing inflorescences (Table 5). Clustering utilized transcript per million (TPM) normalized expression values to iteratively calculate pair-wise manhattan distances between all clusters and joining clusters of proximity. Branch length represents the distance between clusters and reflects the similarity of expression profiles for co-expressed genes within the two clusters

**Fig. 4:**
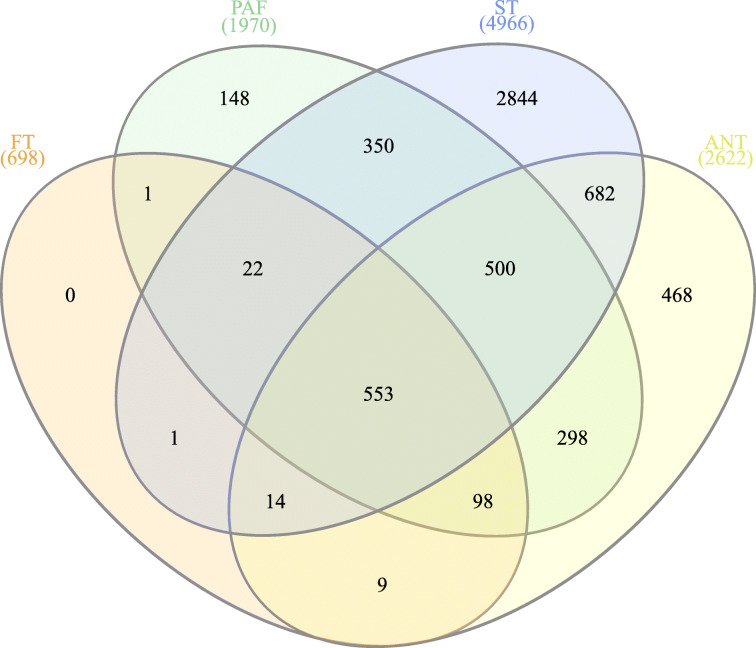
Venn diagram of late spikelet and flower development. Number values represent unigenes with an absolute minimum two-fold change in gene expression (FDR *p* < 0.05). DEGs were derived from statistical tests within floral tissue relative to developing inflorescence controls for boot stage florets (FT), pre-anthesis florets (PAF), anther tissue (ST), anthesis florets (ANT)

Sequencing of vegetative, developing inflorescence, floret, and seed tissues yielded 687 million raw reads (Supplemental Table 1). The primary de novo assembly utilized 15.4 million paired and quality-trimmed reads and comprised 95% of all quality-filtered reads yielding an assembly with 156,724 contigs (N50: 1265; Table 1; Supplemental Fig. 1a-c). BUSCO analysis, utilizing the 956 Plantae core set, revealed an 85.6% completion rate (Fig. 1). Alignment of the transcriptome contigs to the draft genome assembly of *T. ravennae* (Maren et al., in preparation) and cluster-based enrichment reduced the contig set by 68%, yielding a transcriptome assembly with 105,307 unitigs (N50: 1494; Table 1). Similarity-based clustering and genomic alignment reduced the transcript set by 33%, reducing the representation of complete conserved orthologs (BUSCO core genes) by 4.5%. The reduced transcript sets functional annotation identified 33,782 unigenes with at least one of the 130,460 annotations (Supplemental Tables 8; 9; Figs. 2, 3 and 4). Across all samples, 41,234 unigenes were expressed greater than five transcripts per million (TPM) in at least two biological samples and two-fold change (absolute value of log_2_ tagwise dispersion values) between two or more samples. Of those unigenes, 36,127 were transcribed and differentially expressed in at least one of the 78 pair-wise tests of differential expression.

**Table 1:** Summary statistics of sequencing reads, assembly, and annotations

Category	Value^**x**^		
	Primary de novo Assembly	Redundancy Reduced Assembly	Collapsed Iso-Seq Set
Raw reads		670,892,713	1,463,943
Total length of raw reads (Mbp)		206,368	288
Quality filtered & trimmed reads		615,064,938	1,270,618
Total length of prepared reads (Mbp)		80,819	146
Total Contigs	156,724	105,307	65,696
Read utilization (%)	95	78	87
Avg. length of contigs	941	1058	2227
Contig size N50	1265	1494	3581
Min. contig length	300	300	80
Max. contig length	21,363	21,363	9831
Total nucleotides in assembly	147,537,886	111,466,222	146,320,100
Annotation Statistics			
InterProScan	156,723 (99%)	105,307 (100%)	48,482 (74%)
BLASTx (nr) hits	85,974 (55%)	45,192 (43%)	59,553 (91%)
Uniquely aligning to genome^y^	99,110 (63%)	95,274 (90%)	63,042 (96%)
Functionally annotated^z^	66,810 (43%)	33,782 (32%)	51,536 (78%)

^x^Columns represent the three assemblies utilized in the differential gene expression analysis. The primary de novo assembly comprised of next-generation sequence reads assembled using a *k-mer* size of 41 and a bubble size of 350 in the Qiagen CLC Genomics Workbench 11.0. The redundancy-reduced assembly was developed via reference mapping to multiple members in the *Andropogonae* and further reduced by cluster enrichment with CD-Hit. The collapsed iso-seq set was developed from pooled RNA samples of the same experimental tissue and collapsed with the Cupcake TOFU pipeline

^y^Transcripts mapped, with GMAP, to a single genomic locus of a preliminary reference assembly with 95% coverage and identity

^z^Unigenes annotated with one or more gene ontology terms

### Differential expression and GO enrichment of inflorescence development

Differential expression analysis of inflorescence samples identified 3463 unigenes with an absolute two-fold minimum change in expression (FDR *p* < 0.05; Figs. 2 and 3; Supplemental Table 5). Basic metabolic processes of carbohydrate biosynthesis, redox processes, and cell wall growth were prominent along with sexual reproduction categories in the hypergeometric annotation tests (*p* < 0.05). Among the unigenes involved in plant reproduction and morphogenesis, MADS-box transcription factors and other floral integrators were prominent in all inflorescence samples. Transcriptome assembly sequences were named as “Trav” followed by a seven-digit number, which have been put in parenthesis here and thereafter. *MADS-box transcription factor 18* (*MADS18* – Trav0007787) [32], *Sorghum bicolor MADS22-X2* (*SbMADS22-*X2 – Trav0022274) [33], *MADS*-*box protein SUPPRESSOR OF OVEREXPRESSION OF CO 1* (*SOC1* – Trav0019736) [34, 35], and *MADS14*-X2 (Trav0004731) [32, 36], the type II subfamily of MADS-box transcription factors, were expressed at elevated transcript abundance levels in all inflorescences sampled. Transcript abundance changes were most prominent in the early phases of inflorescence development, with 2712 (69%) differentially expressed genes (DEGs) that accounted for most of the upregulated expression (Fig. 2). Early inflorescence development marked transcript abundance changes within basic developmental processes and flavonol and lignin production with *CAFFEIC ACID O-METHYLTRANSFER*AS*E* (*COMT* – Trav0002740) [37, 38]. Several floral development genes, including multiple expansin proteins, zinc finger protein *CONSTANS*-like unigenes [23, 34, 35]*, HEADING DATE 3B-*like (*HD3B*)-like (Trav0007479) [34]*, MADS47-X1* (Trav0004133), *MADS50* (Trav0015948), *MADS55* (Trav0005904), *MADS56* (Trav0017955) [32, 33, 39–41]*,* and *GRAIN NUMBER, PLANT HEIGHT, AND HEADIN*G *DATE7* (*Ghd7* – Trav0011675) [23, 42, 43] were upregulated coinciding with the transition from vegetative to reproductive growth. The abundance of DEGs diminished after the vegetative to inflorescence development shift. As inflorescences matured into the middle and later stages of development, DEG abundance increased. Unigenes relevant in floral meristem determinacy, meiosis, ovule, carpel, and stamen development increased including *PARTING DANCERS* isoform X1 (*PTD*)-X1 (Trav0016008) [44]*, CHROMOSOME TRANSMISSION FIDELITY 7* (*CTF7* – Trav0008006) [45]*, POLLEN DEFECTIVE IN GUIDANCE 1* (*POD1* – Trav0005801) [46, 47], *SELF-PRUNING* (*SP* – Trav0029292) [48]*, AGAMOU*S *LIKE 6-like MADS-box transcription factor* (*AGL6*)-like (Trav0022760) [41] as well as *MADS3-X1* (Trav0008160), *MADS7* (Trav0000111), and *MADS8* (Trav0000081) [32, 33, 39–41]. The DEG abundance substantially increased as inflorescences grew and expanded beyond the flag leaf. Unigenes involved in plant metabolic, putative floral development processes in the embryo sac, meiosis, and pollen development were identified including with *ETERNAL TAPETUM 1* (*EAT1* – Trav0038135) [49, 50]*, MEIOSIS ARRESTED AT LEPTOTENE 1* (*MEL1* – Trav0018140) [50, 51], *Expansin*-*B9* and -*B11* (*EXPB9* – Trav0000513 & *EXPB11* – Trav0000400) [52], *FLOWERING LOCUS T protein* (*FT* – Trav0015919) [53], and *floral homeotic protein AGAMOUS*-like (*AG*)-like (Trav0011776) [54].

Throughout inflorescence development, multiple unigenes and those possessing functional annotations were revealed in the differential expression analyses. Relative to vegetative controls, inflorescence samples at the earliest stages of development had the greatest abundance of DEGs, corresponding with the floral transition (Fig. 2). The fold change in gene expression varied considerably and ranged to greater than ±8000 times the expression levels observed in the vegetative meristem control samples. Table 2 enumerates a list of genes of interest with tissue-specific expression patterns, and those whose fold changes in gene expression were most pronounced for a given stage of inflorescence development. The genes listed in Table 2 comprise a shortlist of utilitarian targets for studying gene functional genomics and gene knockout mutations that might serve to limit reproduction. This information furthermore provides the opportunity for the identification of tissue-specific promoters.

**Table 2:** Unigenes upregulated with stage specific expression patterns in *Tripidium* inflorescence development

Gene name	Unitig ID	E-value^x^	% Id^x^	Fold Change^y^	FDR *p*-value^y^	TPM Normalized Means						
						VM	20P	40P	80P	120P	160P	200P
Inflorescence meristems at 20 cm (20P)												
Mini zinc finger protein 1	Trav0045635	6.1E^−28^	85.5	67.9	2.8E^−3^	1.7	106.9	16.1	59.7	1.6	3.1	2.9
Dehydrodolichyl diphosphate synthase 2	Trav0048752	3.3E^−93^	89.3	48.8	7.7E^−3^	0.7	35.0	1.2	20.1	0.7	1.5	1.7
n/a	Trav0022235	n/a	n/a	48.7	3.1E^−3^	9.6	400.4	13.4	123.6	9.6	11.8	13.6
Transcription factor *TGA4*	Trav0031073	3.1E^−123^	87.8	23.1	8.6E^−4^	7.5	153.9	14.9	51.8	4.9	5.6	6.5
n/a	Trav0012164	n/a	n/a	17.2	4.3E^−3^	28.2	424.0	21.5	120.0	8.9	16.4	17.6
Inflorescence meristems at 40 cm (40P)												
n/a	Trav0005961	n/a	n/a	402.5	2.1E^−6^	0	8.5	52.7	29.3	28.7	26.4	19.8
n/a	Trav0020850	n/a	n/a	203.4	2.1E^−5^	0	10.4	26.3	25.2	24.7	14.4	8.7
Inflorescence meristems at 80 cm (80P)												
MADS-box trans. factor 34	Trav0005828	4.2E^−103^	86.8	210.4	5.5E^−5^	0.2	2.7	2.2	68.2	144.2	168.5	180.8
Inflorescence meristems at 120 cm (120P)												
n/a	Trav0000921	n/a	n/a	204.8	5.0E^−2^	2.1	7.5	0.7	4.7	367.0	752.9	1063.1
Sucrose-phosphate synthase	Trav0002204	0	93.7	109.1	1.9E^−6^	0.8	7.0	4.4	12.1	35.4	91.2	146.0
MADS-box trans. factor 7	Trav0000111	3.6E^−171^	92.0	202.0	1.4E^−3^	0.5	1.1	0.3	10.0	101.9	171.3	233.0
Inflorescence meristems at 160 cm (160P)												
n/a	Trav0012650	n/a	n/a	104.4	5.0E^−6^	0.7	5.2	3.2	7.6	25.4	69.5	60.4
Carbonic anhydrase	Trav0005356	5.3E^−58^	96.6	152.0	4.0E^−2^	4.7	24.0	0.4	3.1	337.7	573.4	571.9
Inflorescence meristems at 200 cm (200P)												
Arginine decarboxylase 2	Trav0036107	0	81.2	1472.0	8.2E^−3^	0.1	0.9	0.1	0.3	41.1	213.8	294.0
n/a	Trav0006980	n/a	n/a	487.1	1.1E^− 3^	2.2	3.0	1.8	2.7	183.0	677.9	959.9
MADS-box trans. factor 8	Trav0000081	4.5E^−175^	96.2	471.5	2.7E^−6^	0.6	1.5	0.5	10.2	107.0	218.1	300.9
Protein unc-13 homolog	Trav0005596	0	90.2	104.5	5.0E^−3^	1.9	3.8	1.4	2.6	61.0	137.7	174.1
MADS-box trans. factor 16	Trav0000760	5.5E^−27^	83.7	87.3	2.8E^−4^	1.0	0.4	0.5	2.9	40.7	70.5	85.2
MADS-box trans. factor 4	Trav0000787	4.6E^−76^	98.3	40.2	7.0E^−6^	3.7	6.3	3.8	9.9	41.7	89.3	139.4
Sister chromatid cohesion 1 protein 1	Trav0023214	0	88.8	34.2	4.5E^− 4^	0.5	1.2	0.5	2.6	6.6	15.1	17.8

^x^E-value and % Id derived from BLAST and InterPro Scan results and annotated utilizing BLAST2GO default annotation rules

^y^Fold change and FDR *p*-values are derived from EDGE tests between the vegetative control and inflorescence development stage of each underlined subsection within the table

### Differential expression and GO enrichment in floral development

**Fig. 5:**
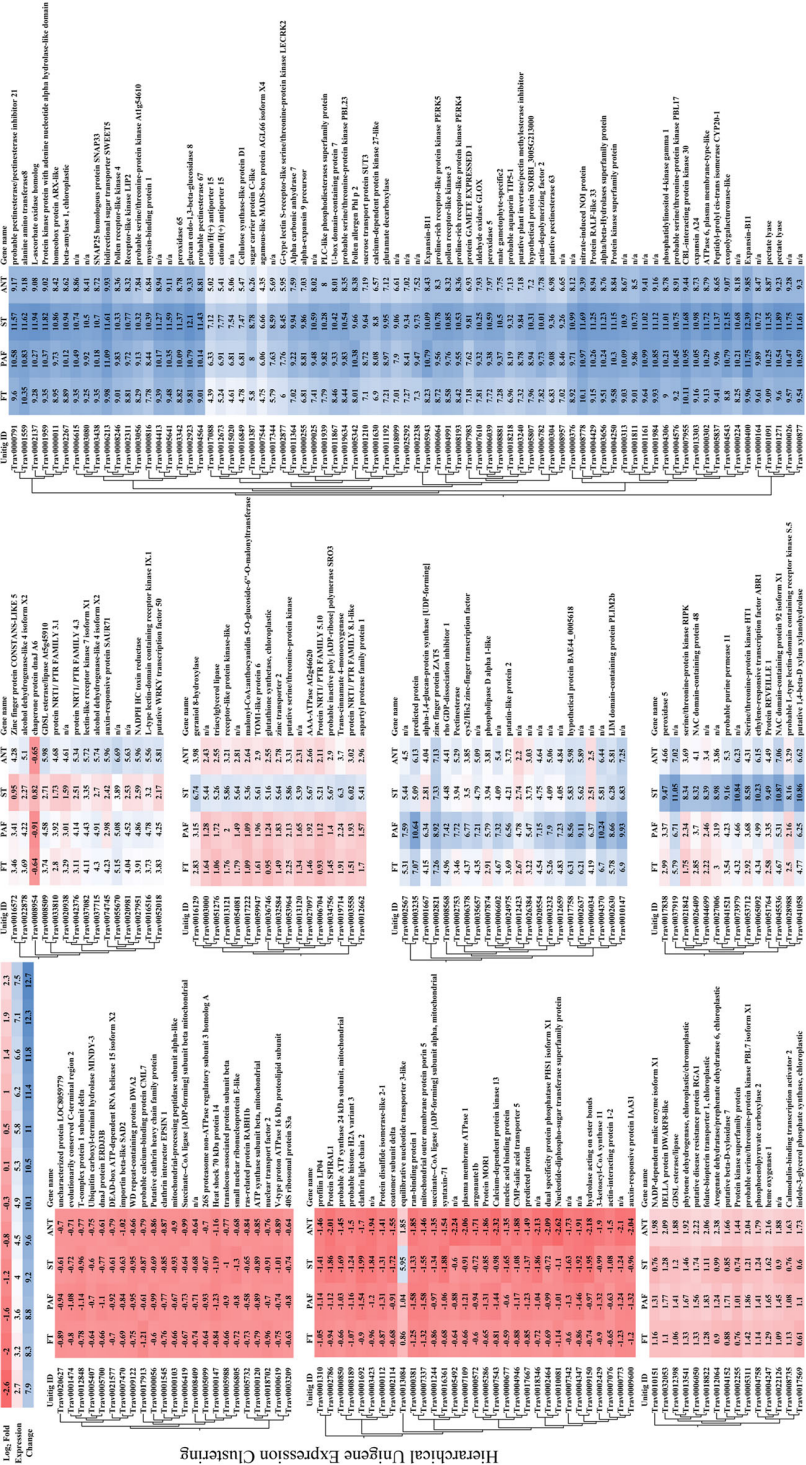
Heat map of hierarchically clustered floral development unigenes. Absolute values of transcript per million normalized gene expression ranged from a minimum of two-fold to > 8000-fold change in expression relative to controls (FDR *p* < 0.05). Columns represent log_2_-fold expression values of all biological replicates in inflorescences at 120 cm of development (120P), developing boot stage florets (FT), pre-anthesis florets (PAF), stamens (ST), and florets at anthesis (ANT) (Table 5). Clustering utilized transcript per million (TPM) normalized expression values to iteratively calculate pair-wise Manhattan distances between all clusters and joining clusters of proximity. Branch length represents the distance between clusters and reflects the similarity of expression profiles for co-expressed genes within the two clusters

Differential gene expression profiling of floret development identified 5988 unigenes with the absolute minimum two-fold change in expression (FDR *p* < 0.05; Figs. 4 and 5; Supplemental Table 6). Among floret development samples, DEG abundance was highest in stamen tissues (4966), followed by anthesis florets (2622), pre-anthesis florets (1970), and early development florets (698). At the earliest stage of floret development, multiple MADS-box transcription factors, including *AGL6*-like [41], were differentially regulated along with *protein DROOPING LEAF* (*DL*)-X1 (Trav0014582) [55] and *AG*-like (Trav0011776) [54]. As florets developed into pre-anthesis florets, differential gene expression analysis revealed multiple genes in floral organ morphogenesis, embryo sac, embryonic, and pollen development relative to late inflorescence development samples. DEGs implicated in floral development included guanine nucleotide-binding protein *NUCLEOSTEMIN LIKE 1* (*NSN1* – Trav0003163) [56, 57], *SOMATIC EMBRYOGENESIS RECEPTOR KINASE* 2 (*SERK2* – Trav0003828) [58], *FLOWERING PROMOTING FACTOR 1-LIKE* 2 (*FPF1*)-2 (Trav0026700) [59], and *BTB/POZ AND TAZ DOMAIN-CONTAINING PROTEIN 3* (*BT3* – Trav0015326) [60]. Relative to late inflorescence development samples, unigenes regulating pollen integument, cellular growth, and karyogamy increased in the DEG sets in pre-anthesis florets and stamen tissues, including protein *NUCLEAR FUSION DEFECTIVE 4* (*NFD4*)-X2 (Trav0000417) [61], and *putative receptor protein CRINKLY 4* (*CR4* – Trav0011689) [62].

Floral tissue samples can provide detailed information to identify genes of interest for future applications of precision breeding and gene editing of reproductive processes. The utility of full-length transcripts to key floral integrators and the potential they have in the identification of multiple gene-editing mutation regions may be a key asset in precision breeding and gene editing. DEG analysis revealed tissue-specific gene expression dynamics in all floral samples. Within flower specific tissues, stamen tissues yielded the greatest abundance of upregulated differential gene expression (Fig. 4). Gene expression dynamics varied widely in stamen samples, with some genes demonstrating a 7000-fold difference in expression relative to late stage inflorescence controls. Genes of interest were amassed into a table based on tissue-specific expression and large fold changes between controls and a given floral sample (Table 3). The tissue expression specificity of these genes provides candidate genes for continued study or applied plant breeding purposes to limit reproduction in *Tripidium*.

**Table 3:** Unigenes upregulated with stage specific expression patterns in *Tripidium* flower development

Gene name	Unitig ID	E-value^x^	% Id^x^	Fold Change^y^	FDR *p*-value^y^	TPM Normalized Means				
						120P	FT	PAF	ST	ANT
Stamens (ST)										
Glucan endo-1,3-beta-glucosidase 8	Trav0002923	0	92.1	4394.1	1.9E^−7^	0.1	208.4	322.4	749.7	156.3
Homeobox protein ARX-like	Trav0000011	1.4E^−112^	79.7	1855.9	6.4E^−12^	2.1	903.4	1226.8	2510.5	678.1
Pollen receptor-like kinase 3	Trav0004991	0	82.4	1850.4	6.4E^−10^	0.2	94.7	167.5	331.5	86.5
Male gametophyte-specific2	Trav0008881	0	89.0	1451.8	1.1E^−10^	0.1	36.8	121.0	242.5	55.7
Pollen receptor-like kinase 4	Trav0008246	0	84.8	1286.6	2.8E^−8^	0.3	174.5	246.1	323.0	122.8
Ethylene-responsive trans. factor ABR1	Trav0065092	5.5E^−33^	74.1	1204.8	3.2E^−27^	0.1	4.8	5.1	208.8	16.4
*GAMETE EXPRESSED 1*	Trav0007983	0	87.8	898.2	8.4E^−12^	0.3	51.6	53.6	232.4	45.4
*REVEILLE 1*	Trav0051764	0	82.0	719.6	3.2E^−34^	3.4	19.3	23.5	1736.8	73.9
Xylanase inhibitor protein 1	Trav0043283	0	91.2	565.4	3.0E^−13^	2.9	66.0	68.7	1142.4	343.9
Florets at anthesis (ANT)										
n/a	Trav0031057	n/a	n/a	2120.1	2.2E-9	0.8	372.7	260.8	1269.9	1753.1
5-pentadecatri-enyl resorcinol O-methyltransferase	Trav0039042	1.9E^−161^	78.1	1345.4	9.4E^−10^	0.3	34.5	12.6	118.1	531.8
Myosin-binding protein 1	Trav0000816	0	78.8	114.9	7.6E^−6^	1.1	227.8	279.5	1010.0	126.2
Pre-anthesis florets (PAF)										
Expansin-B11	Trav0005943	2.3E^−39^	93.3	1765.5	7.8E^−12^	0.2	80.9	371.5	206.4	0.2
Predicted protein	Trav0003235	0	78.9	1600.0	1.4E^−6^	0.1	27.8	263.7	6.3	16.2
Pollen allergen *Phl p 2*	Trav0005342	7.9E^−48^	80.9	810.2	9.7E^−10^	0	31.4	127.6	71.2	45.8
Zinc finger protein *ZAT5*	Trav0002821	2.6E^−88^	55.8	485.6	3.9E^−6^	0.1	36.4	91.3	28.3	0.1
Boot stage florets (FT)										
AG-like MADS-box protein *AGL66* X4	Trav0007544	0	82.8	26.9	5.6E^−3^	0.4	11.8	22.7	32.7	9.8
Retrotransposon protein, putative, unclassified	Trav0016899	0	63.8	24.5	0.04	0.3	9.8	11.8	7.5	17.1
auxin-responsive protein *SAUR71*	Trav0074745	6.8E^−40^	81.7	18.8	0.02	0.2	6.3	1.8	1.2	21.4
Zn finger protein *CONSTANS-LIKE 5*	Trav0016572	8.3E^−19^	83.0	11.0	4.3E^−3^	9.6	94.5	67.2	12.8	172.3

^x^E-value and % Id derived from BLAST and InterPro Scan results and annotated utilizing BLAST2GO default annotation rules

^y^Fold change and FDR *p*-values for each gene are derived from EDGE tests between inflorescence meristem samples at 120 cm of development and each underlined subsection within the table

### Differential expression and GO enrichment in seed development

**Fig. 6:**
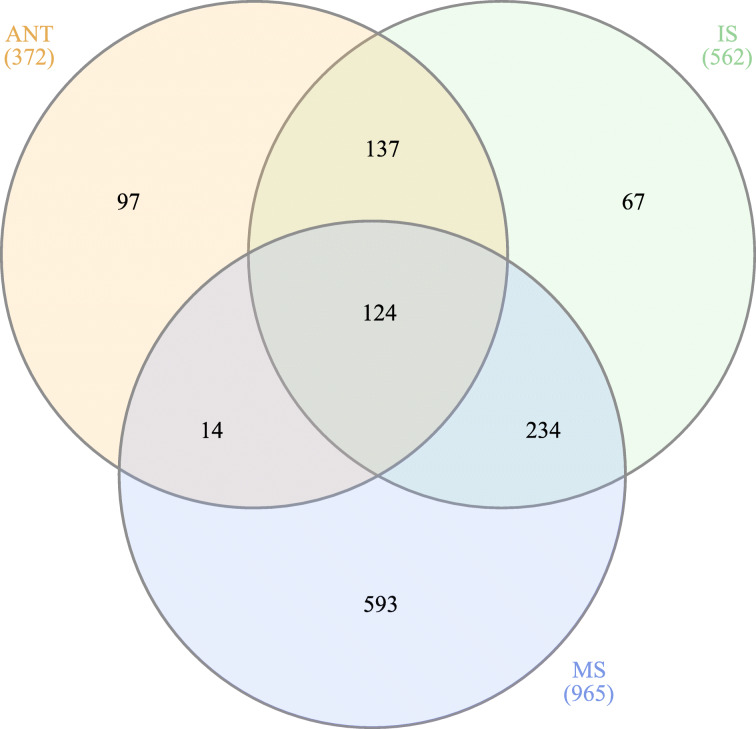
Venn diagram of seed development. Number values represent unigenes with an absolute minimum two-fold change in gene expression within seed tissue relative to controls (FDR *p* < 0.05). DEGs derive from statistical tests within seed tissues relative to developing inflorescence controls for florets at anthesis (ANT), florets with immature seeds (IS), and florets with mature seeds (MS)

**Fig. 7:**
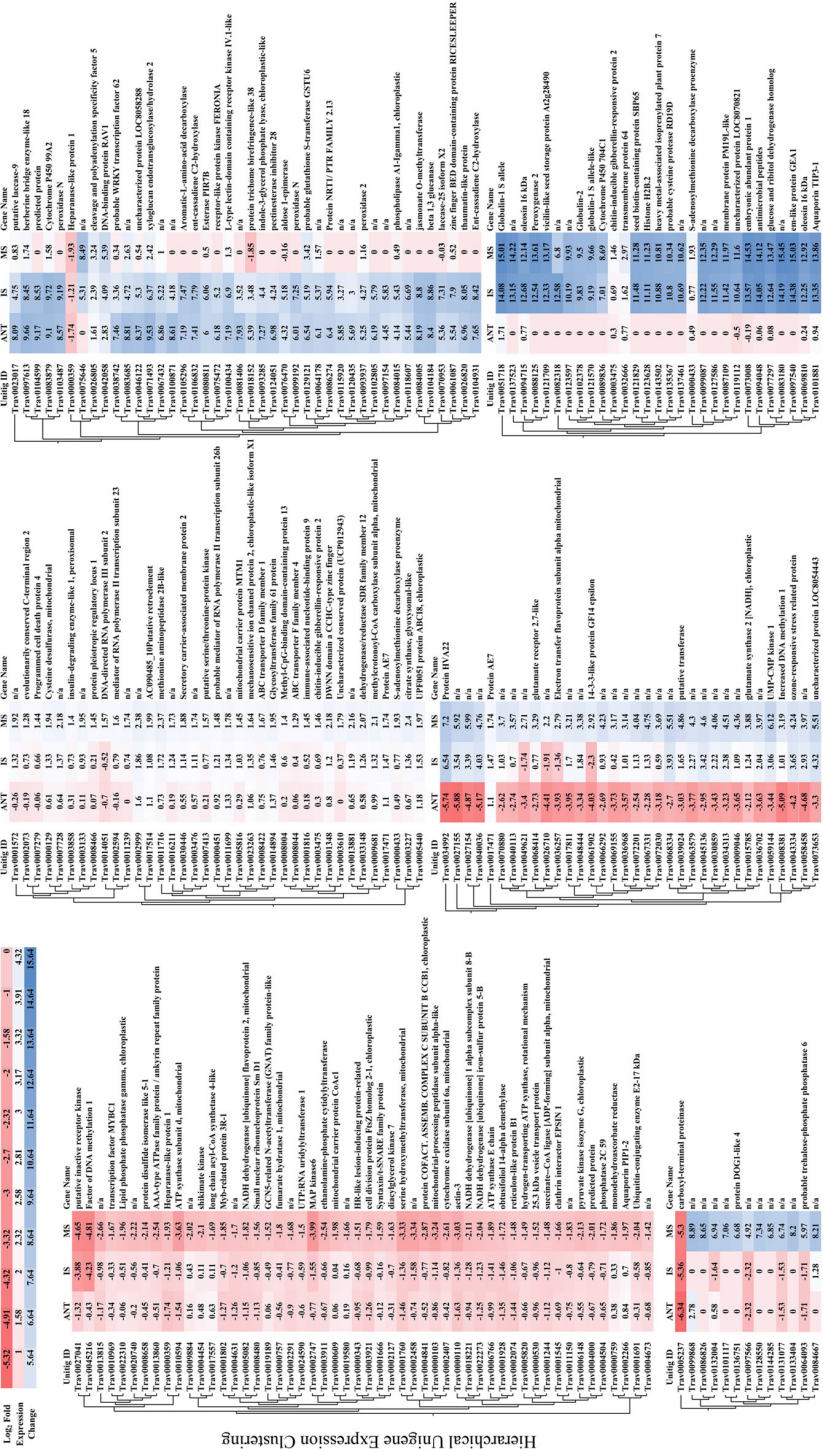
Heat map of hierarchically clustered seed development unigenes. Absolute values of transcript per million normalized gene expression ranged from a minimum of two-fold to > 8000-fold change in expression relative to controls (FDR *p* < 0.05). Columns represent log_2_-fold expression values of all biological replicates of developing florets at anthesis (ANT), immature seed (IS), and mature seed (MS) (Table 5). Clustering utilized transcript per million (TPM) normalized expression values to iteratively calculate pair-wise manhattan distances between all clusters and joining clusters of proximity. Branch length represents the distance between clusters and reflects the similarity of expression profiles for co-expressed genes within the two clusters

Differential gene expression profiling of seed development identified 1266 unigenes with an absolute minimum two-fold change in expression (FDR *p* < 0.05; Figs. 6 and 7; Supplemental Table 7). GO term enrichment analysis identified 654 terms throughout seed development. Many GO terms coincided with basic organismal processes and development. Mature seed samples had the highest number of DEGs (965) relative to immature seed (562) or anthesis florets (372). In the mature seed, several DEGs involved in multicellular organism development and seed maturation were identified. The functional annotation of these sequences indicated that they were regulatory protein *VIVIPAROUS 1* (*VP1* – Trav0080007) [63], *WUSCHEL-related homeobox 6* (*WOX6* – Trav0041173) [64], *LATE EMBRYOGENESIS ABUNDANT 18* (*LEA18* – Trav0122482) [65], and *SUCROSE SYNTHASE 3* (*SUS3* – Trav0008473) [66]. In the transition from anthesis florets to immature seed development, DEG analysis identified 80 DEGs with multiple unigenes in embryo development, seed oil-body biogenesis, and response to abscisic acid. Noteworthy annotations included *OIL BODY-ASSOCIATED PROTEIN 1A* (*OBAP1A* – Trav0053823) [67], *VICILIN*-like (*VIC*)-like (Trav0081216) [68], *LATE EMBRYOGENESIS ABUNDANT EMB564* (*LEA-EMB564* – Trav0096979) [65], and dehydrin *Rab25*-like (*Rab25* – Trav0064814) [69].

Seed production is a significant contributor to the successful cultivation of many plants. In developing seed samples, a variety of genes with increased expression levels involved in homeostatic regulatory roles for metal ion uptake, redox processes, heat shock, and genes known to interact under abiotic stress were found in the dataset. Fold changes in gene expression between late inflorescence stage development controls and mature seeds varied considerably and were expressed at as much as a 16,000-fold difference (Fig. 6). A shortlist of genes of interest within these analyses were amassed in Table 4 because of their tissue-specific expression pattern. Taken together, these genes represent several potential targets that may drastically limit plant reproductive characteristics in an applied precision breeding context.

**Table 4:** Unigenes upregulated with stage specific expression patterns in *Tripidium* seed development

Gene name	Unitig ID	E-value^x^	% Id^x^	Fold Change^y^	FDR *p*-value^y^	TPM Normalized Means			
						200P	ANT	IS	MS
Mature seeds (MS)									
Globulin-1 S allele	Trav0051718	0	83.5	32,919.7	7.1E^−7^	0.4	1.4	7883.0	16,578.5
Embryonic abundant protein 1	Trav0073008	4.2E^−41^	95.7	23,575.7	5.8E^−6^	0.26	0.19	4424.2	9490.7
Aquaporin TIP3–1	Trav0101881	1.8E^−130^	94.0	14,828.8	8.4E^−6^	0.1	0.28	2266.5	3572.9
Vicilin-like seed storage protein At2g28490	Trav0121709	3.8E^−87^	87.7	9135.7	1.6E^−5^	0	0	628.7	1237.7
n/a	Trav0084667	n/a	n/a	281.5	6.7E^−16^	0	0	0.2	40.1
n/a	Trav0133404	n/a	n/a	276.5	1.4E^−9^	0	0	0	40.3
Immature seeds (IS)									
Em-like protein GEA1	Trav0097540	3.7E^−49^	87.0	21,364.3	1.5E^−4^	0	0	2616.3	4543.1
Oleosin 16 kDa	Trav0094715	1.9E^−45^	77.2	7209.0	2.2E^−5^	0.1	0.2	1195.2	907.0
n/a	Trav0099087	n/a	n/a	5148.0	9.4E^−5^	0	0	584.4	707.9
Seed biotin-containing protein SBP65	Trav0121829	1.7E^−50^	76.5	3075.0	9.0E^−5^	0	0	348.7	334.6
Metal-assoc. isoprenylated plant protein 7	Trav0143502	1.5E^−55^	77.0	2029.7	6.4E^−4^	0	0	230.2	244.4
n/a	Trav0137461	n/a	n/a	1776.7	1.7E^−4^	0	0	201.9	212.6
Globulin-2	Trav0102378	7.8E^−59^	91.5	953.5	8.3E^−5^	0	0	109.6	96.3
Cytochrome P450 99A2	Trav0083879	0	78.5	842.3	3.7E^−4^	0	78.4	93.3	0.3
Florets at anthesis (ANT)									
Berberine bridge enzyme-like 18	Trav0097613	0	91.1	807.0	6.7E^−6^	0	100.3	36.4	0.3
Xyloglucan endotransglucosylase/hydrolase 2	Trav0071493	1.4E^−74^	77.7	790.4	7.8E^−3^	0	87.2	7.4	0.6
n/a	Trav0083685	n/a	n/a	470.4	2.0E^−3^	0	47.1	2.8	0.7
n/a	Trav0100871	n/a	n/a	418.9	4.9E^−5^	0	45.4	1.9	0
Uncharacterized protein LOC8058288	Trav0046122	0	72.6	363.8	2.5E^−3^	0.1	74.6	7.0	0.2
n/a	Trav0081406	n/a	n/a	257.9	1.3E^−4^	0	26.3	1.1	0
Ent-cassadiene C2-hydroxylase	Trav0106832	3.1E^−24^	80.5	186.9	0.01	0	21.2	24.0	0

^x^E-value and % Id derived from BLAST and InterPro Scan results and annotated utilizing BLAST2GO default annotation rules

^y^Fold change and FDR *p*-values for each gene are derived from EDGE tests between inflorescence meristem samples at 200 cm of development and each underlined subsection within the table

### Gene expression analysis and validation of selected candidate genes by real-time qPCR

**Fig. 8:**
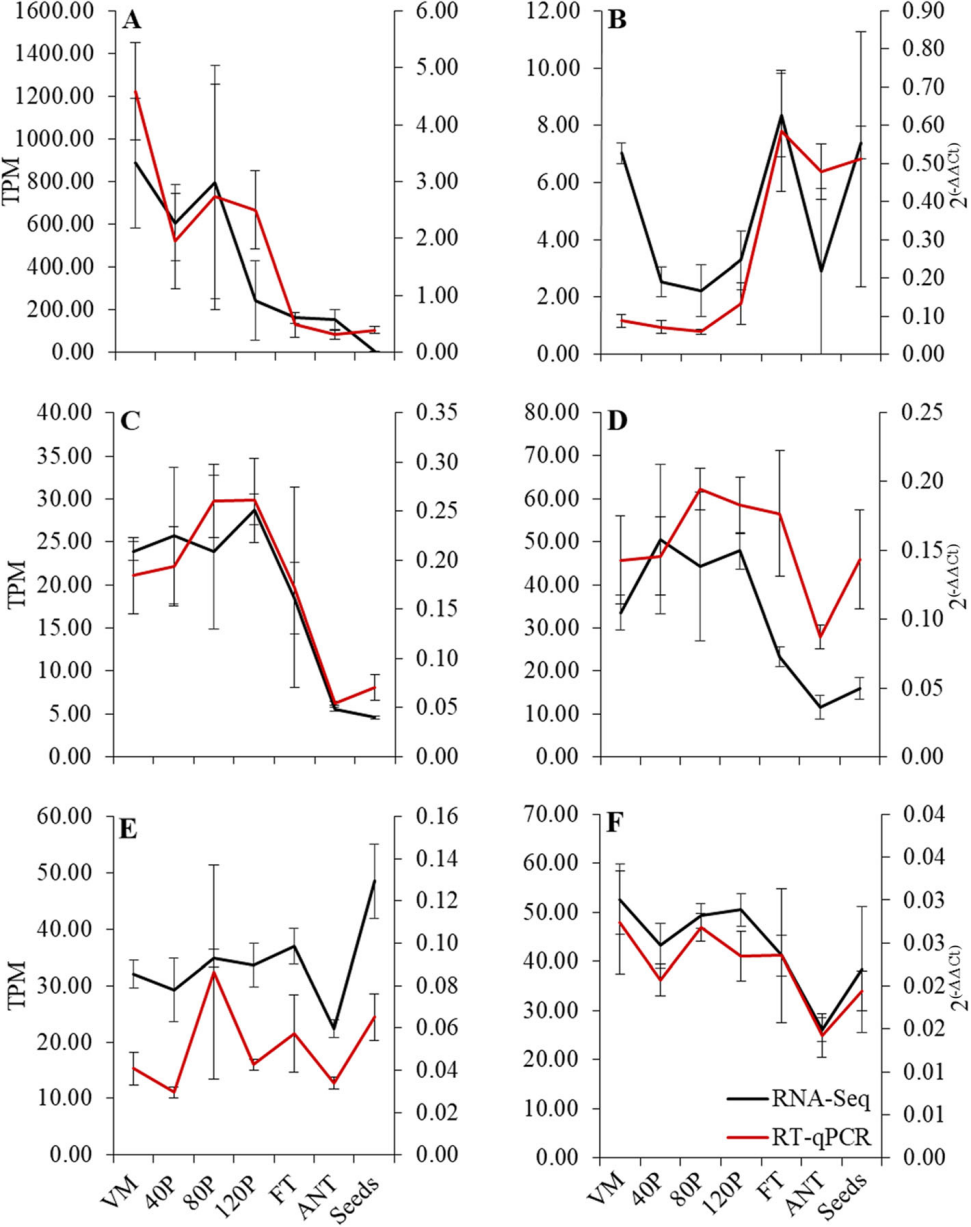
Validation of RNA-Seq expression levels with qPCR. Black and red lines represent relative gene expression levels observed in the RNA-Seq and qPCR respective datasets. RNA-seq data are transcript per million (TPM) normalized means ± SEM. Relative gene expression of qPCR data utilized using BI1-like protein gene and translationally controlled tumor protein homolog *TCTPH* as reference genes and calculated with the 2^(-ΔΔCt)^ algorithm. **a** Trav0002740 (caffeic acid 3-O-methyltransferase). **b** Trav0032568 (NUCLEAR FUSION DEFECTIVE 4 isoform X2). **c** Trav0016008 (PARTING DANCERS isoform X1). **d** Trav0008006 (CHROMOSOME TRANSMISSION FIDELITY 7). **e** Trav0015779 (RNA-DIRECTED DNA METHYLATION 4). **f** Trav0014920 (POLLEN DEFECTIVE IN GUIDANCE 1)

The expression patterns between RNA-Seq data and qPCR data demonstrated a positive mean Spearman rank correlation (Fig. 8). Overall, the six genes selected for the assessment validated similar relative rank and transcript abundances throughout development. *CAFFEIC ACID 3-O-METHYLTRANSFERASE* (*COMT* - Trav0002740) is involved in the lignin biosynthetic pathway [38]. In *Tripidium,* unigenes designated with this annotation expressed abundantly in vegetative meristematic tissues and declined throughout inflorescence, floral, and seed development. *NUCLEAR FUSION DEFECTIVE 4* (*NFD4* – Trav0032568) is a gene of interest for its role in karyogamy or nuclear fusion following pollination and during female gametophyte formation [61]. Overall transcript abundance was low and increased during the critical phases of floral development. *PARTING DANCERS 1* (*PTD1* – Trav0016008) is essential in cross-over formation during meiosis [44]. Reports of transcriptional activity of *PTD1* in rice place the highest activity in anthers and pistils at heading [44]. The notable difference in the number of possible meiocytes sampled in developing inflorescences versus those of floral development tissues likely explains some of the disparity in expression patterns between *Tripidium* and rice. *CTF7* functions in the plant body during double-stranded break repair associated with cell division. The expression dynamics of this DNA repair associated protein expressed abundantly during active phases of inflorescence growth. *RNA-DIRECTED DNA METHYLATION 4* (*RDDM4* – Trav0015779) is putatively involved in the epigenetic modification system of plants [70]. Expression of *RDDM4* in *Tripidium* was relatively consistent throughout inflorescence, diminished in anthesis floral samples, and increased in mature seeds. *POLLEN DEFECTIVE IN GUIDANCE 1* (*POD1* – Trav0014920) dysfunction in *Arabidopsis* has demonstrated major impacts on vegetative and reproductive growth [46, 47]. The expression of *POD1* in *Tripidium* demonstrated relatively consistent expression throughout inflorescence and floral development.

## Discussion

This study aimed to scrutinize the gene regulatory environment of developing inflorescences, flowers, and seeds to maximize the knowledge base for transgenic and gene editing based plant improvements. Our approach examined both within and between-group testing to identify transcript abundance changes specific to a given stage of development.

### Inflorescence development and floral transition

The abundance of differentially expressed unigenes revealed the magnitude of gene expression changes involved in floral induction. The transition from the vegetative meristem to the reproductive meristem follows an abundance of regulatory changes coinciding with the increased expression of homeobox genes, MADS-box transcription factors, and plant hormones [28]. The members of the expansin family were significantly over-represented in the hypergeometric annotation tests as well as the differential expression analysis, which identified nine different transcript annotations of expansins as well as other canonical floral induction integrators. As culms produced new vertical growth, the upregulation of multiple expansin family members validated their role in the reproductive process of *Tripidium* [71–73].

Many of the classic ABC(D) E model MADS-box transcription factors are essential in the fate of meristem identity and determinacy [41, 74, 75]. Within the developing inflorescence, 21 different MADS-box elements demonstrated differential expression patterns. The expression dynamics and identification of *MADS18*, *SbMADS22*-X2, *MADS-box protein SOC1*, and *MADS14*-X2 [36, 40] in inflorescence meristem samples demonstrate the conservation of roles of these floral organ identity transcription factors in *Tripidium*. *FLOWERING LOCUS T* (*FT*), *HEADING DATE 3A* (*HD3A*) [76], and *VERNALIZATION 3* (*VRN3*) [77] are synonyms for florigen, now well characterized in *Arabidopsis*, rice, and wheat [21, 53, 78]. The most significant sequence homology of the *Tripidium* florigen transcript identified *FT* (*TrFT* – Trav0056419) within the transcriptome. Within inflorescence development samples, *TrFT* demonstrated the highest expression levels in the floral transition (20P), as reported in *Sorghum bicolor* [53]. As an inflorescence matures into the late stages of development, the determinacy of the meristems changes in several plant species. *SELF-PRUNING* (*SP*) is the homolog of *CENTRORADIALIS* and *TERMINAL FLOWER 1* of *Arabidopsis* and functionally maintains floral meristem indeterminacy [48]. The *SP* expression patterning in *Tripidium* followed expectations with consistent expression throughout inflorescence development. The floral homeotic protein *AG*-like genes increased in expression in *Tripidium* inflorescences throughout maturation. *AG*-like genes are broadly involved in floral development and coordination of the floral body plan; therefore, it might have a role in *Tripidium* floral transition and organization. Concomitant decreases in *WUSCHEL*-related homeobox expression were observed as *AGL* increased, capitulating the relationship between these two interacting genes in *Tripidium* [79].

In rice and barley, their respective *CONSTANS-like 9* (*COL9*) and *CONSTANS1* (*CO1*) expression increases in the early weeks of plant development and diminished following the floral transition [21, 35]. As *COL9* abundance represses *EARLY HEADING DATE 1*, delaying flowering in rice, *COL9* (Trav0008717) expression diminished in *Tripidium* with concomitant increased *HEADING DATE 3* (*HD3*)-like transcription from early to mid-inflorescence stages of development [21, 80, 81]. *Grain number, plant height, and heading date 7* (*Ghd7*) is a photoperiodic responsive gene with upstream repressive effects on *EHD1* and subsequently *HD3A* in rice [76]. The translational dynamics of *Ghd7* in *Tripidium* support a role throughout inflorescence development [23, 42, 43, 76].

### Floral and reproductive development

The identity genetics of floral development in grasses consists of several MADS-box transcription factors and their interacting proteins in articulating the floral body plan. The translation of floral homeotic class genes from the core eudicots to the monocots is tenuous due to various synonyms applied with computationally derived annotations. The road map best suited to this disambiguation lies in the consensus between expression dynamics and functional genomics. The *AGL6-like* MADS-box transcription factor is active in perianth and gynoecium development in rice with pleiotropic effects on lodicules, stamens, and carpels [82]. In *Tripidium*, the expression of *AGL6*-like follows similar patterns of expression in developing inflorescences and floral tissues reported in *Oryza sativa* [41], supporting its floral organ-specific expression and the significance of its role in floral organ specification. The role of floral organ specification and meristem determinacy are also functions of the *YABBY* genes *DL1* and *DL2* in rice carpel specification and maize [55, 83, 84]. While the tissue expression specificity between branch meristems and inflorescence meristems was not established in these experiments, the dynamic gene expression profile in inflorescence and developing floral tissues support its role in *Tripidium* floral determinism [55, 83, 84]. In wheat, the *TaAGL6* acts on staminate floral development by working on *TaAPETALA3* [85]. In *Tripidium*, the homeotic activity of staminate floral development could be identified by the dramatic increase in transcript abundance of floral homeotic protein *AG*-like in samples at 80 cm of growth.

### Meiosis, embryogenesis, and caryopsis development

As inflorescences matured into the middle stages of development and beyond, transcript abundance increased. Among the changes in transcript abundance, multiple meiotic genes delineated the next phase in reproduction. Transcription factor *EAT1* is a constituent of the *TAPETUM DEGENERATION RETARDATION* (*TDR*) heterodimer significant in tapetum development [86, 87]. Interestingly, the expression pattern of *EAT1* follows a distribution significant in both early and post-meiosis cells [50]. This expression pattern, and our observations in *Tripidium*, support mid-inflorescence stage microsporogenesis. The conformity of expression patterns of the *POD1* transcript to those reported by Dai et al. [46] furthermore provides evidence for the conservation of function in *Tripidium*. It is noteworthy that the sampling strategy employed in this study was sufficient to successfully identify unigenes of protein *PTD*, *CTF7,* and *SHORTAGE IN CHIASMATA I*. Differential expression analysis of meiotic unigenes in developing inflorescences highlights their significance in the reproductive process as well as the abundance of meiocytes captured here. A more comprehensive characterization of the regulatory dynamics of meiocytes involved in mega- and microsporogenesis will require additional examination in *Tripidium* [44, 45, 88].

The translational dynamics of floral development is foundational to plant reproduction. When the inflorescence has emerged from the flag leaf, the changes in floral morphology are apparent, but many of the morphological changes are manifestations of the developmental design laid out during inflorescence development. Many of the genes responsible for gamete production and flower development were highly expressed at the mid-inflorescence stages of development. One of the primary benefits of the sampling strategy and data analysis approaches used in this study has been to sample multiple stages of development and to compare all possible pair-wise comparisons. This has enabled the capitulation for several canonical floral genes in a novel species as well as highlighted the sequence identity of multiple novel genes.

## Conclusion

*Tripidium* is an enigmatic plant with considerable potential as a landscape and bioenergy crop given its ornamental merit and high biomass production on marginal lands with minimal inputs. A greater understanding of gene expression throughout reproductive development provides context for gene function analysis in these basic biological processes. This research focused on the molecular genetics of plant reproduction in *T. ravennae,* including identifying diverse genes related to inflorescence, flower, and seed development. The expression dynamics of unigenes detailed in this study provide a guide for future biological studies on functional and comparative genomics and the development of biotechnology applications, including precision breeding.

## Materials and methods

### Plant material and sample collection

Vegetative and inflorescence meristems as well as floral development tissues were collected from three plants on the week of August 9th, 2017, from the North Carolina Arboretum, Asheville, NC. Inflorescence meristematic tissue from reproductive culms was collected at various heights from the ground, representing floral development progression. Inflorescences had emerged from more developed culms, but inflorescence meristems at earlier development stages were apparent within the leaf sheath of immature culms. Floral development tissues were collected at the floret boot stage, pre-anthesis, anthesis stages, and mature stamens. Spikelets containing immature and mature seeds were collected from the same plants in September and October of 2017. Culm segments, inflorescences, and developing seed samples containing target tissues were collected and immediately placed in 15 or 50 mL centrifuge tubes vials with 5–20 mL of RNAlater® Stabilization Solution (Ambion®, Life Technologies TM). Centrifuge tubes containing sample tissue were frozen in the field on a dry ice bed before transport to the laboratory and stored at − 80 °C (Table 5). Excess plant tissue was trimmed and removed or enriched under a stereomicroscope in sterile 100 mm Petri dishes containing approximately ~ 5–10 mL fresh RNAlater®. Immature floret tissue samples (FT) were purified from bulk collected inflorescence tissue before emergence from the flag leaf sheath. Pre-anthesis floret tissue samples (PAF) were purified from bulk collected inflorescence tissue that had emerged from the flag leaf sheath. The observation of spikelet expansion and glume extrusion identified PAF samples, but no evidence of anther or stigma extrusion was observed. Stamen tissue samples (ST) were purified from bulk collected floral spikelet tissues when anthers were exposed entirely outside of the glumes, and pollen was visibly dehiscing. Samples of florets at anthesis (ANT) were purified from bulk collected floral spikelet tissues by amassing florets showing stigma protrusion from the floret’s lemma and palea. Immature and mature seeds were processed by removing first, and second-order rachilla from the bulk collected tissue before tissue lysis and homogenization. Sample tissue lysis and homogenization were processed in liquid nitrogen by mortar and pestle.

**Table 5:** List of samples, their codes, and corresponding sampling heights of tissues collected for RNA-seq analysis

Tissue	Sample code	Plant A^w^ (cm)	Sample code	Plant B^w^ (cm)	Sample code	Plant C^w^ (cm)
Inflorescence development tissues^x^						
VM (Vegetative meristem)	VMA	0	VMB	0	VMC	0
20P (~ 20 cm) meristem	20PA	16	20 PB	21	20PC	13
40P (~ 40 cm) meristem	40PA	25	40 PB	40	40PC	44
80P (~ 80 cm) meristem	80PA	60	80 PB	106	80PC	70
120P (~ 120 cm) meristem	120PA	133	120 PB	121	120PC	120
160P (~ 160 cm) meristem	160PA	170	160 PB	157	160PC	170
200P (~ 200 cm) meristem	200PA	203	200 PB	193	200PC	198
Floral development tissues^y^						
FT (boot stage florets)	FTA	n/a	FTB	n/a	FTC	n/a
PAF (pre-anthesis florets)	PAFA	n/a	PAFB	n/a	PAFC	n/a
ANT (Florets at anthesis)	ANTA	n/a	ANTB	n/a	ANTC	n/a
ST (Stamens)	STA	n/a	STB	n/a	STC	n/a
Seed development tissues^z^						
IS (Immature seeds)	ISA	n/a	ISB	n/a	ISC	n/a
MS (Mature seeds)	MSA	n/a	MSB	n/a	MSC	n/a

^w^Measurements are the actual distance (cm) from the base of the culm to the inflorescence node subtending the meristem for each sample

^x^20P, 40P, 80P, 120P, 160P, and 200P represented six sampling groups for inflorescence development and grouped by the approximate distance from the base of the culm where those samples were collected

^y^FT, floral spikelet tissue excised from boot stage inflorescences. PAF, floral spikelet tissues sampled from mature inflorescences before stigma extrusion from the palea and lemma. ANT, samples at the stage of floret development where stigmas were pollen receptive and bright red. ST, samples of fresh dehiscent anther tissues

^z^IS, samples of floret tissue approximately one-two weeks post-anthesis. MS, mature florets

### RNA isolation, library preparation, and sequencing

Total RNA was extracted from all tissues using the Spectrum® Plant Total RNA Kit (Sigma-Aldrich, Burlington MA). DNA was digested on-column with the Sigma-Aldrich DNase10 (DNASE10) kit per the manufacturer’s instructions. RNA concentration and integrity were quantitated with the QubitTM fluorimeter (Life TechnologiesTM) and the 2100 Bioanalyzer (Agilent) before library preparation, respectively. RNA samples were poly-adenylation purified, and cDNA libraries were prepared using the BiooScientific (a PerkinElmer Co.) NEXTFlex Rapid Directional RNA-Seq kit with a target insert size of 200–300 bp. Libraries were sequenced using the Illumina HiSeq 4000, 150 bp PE by Novogene (Sacramento, CA). The RNA of all samples was mixed and used to construct Pacific Biosciences Iso-Seq libraries (Protocol # 101–070-200 version 6) with three size fractions (no size selected, < 4 kb, and > 4 kb). The libraries were sequenced with four cells of a PacBio Sequel I system at NC State Genomic Sciences Laboratory.

### Transcriptome assembly and functional annotation

Read quality was inspected for quality with FastQC. Trimming was conducted with CLC Genomics WB (CLC – GWB, V11.0.1, QIAGEN) to remove adapter sequences and low-quality reads (Q < 20). Multiple de novo transcriptome assemblies were constructed with the CLC – GWB using different k-mer (word size) and the bubble size of de Bruijn graph combinations and assessed for the number of contiguity and N50 (Supplemental Fig. 1a). The assembly with the lowest number of contigs but the largest N50 was selected which maximized the yield of complete BUSCOs (Supplemental Fig. 1b & c). The final assembly was mapped (GMAP, V2015-07-23, [89]) to a draft genome assembly of *T. ravennae* (Maren et al., Unpub.) as well as multiple reference genomic assemblies within the *Andropogoneae* tribe, including *Sorghum bicolor* [46], *Saccharum officinarum* [90], and *Zea mays* [91]. The GMAP mapping was carried out to enrich the transcriptome for plant transcripts and eliminate the transcripts of sample surface contaminants [92]. Contigs with a 95% identity and match score to two or more reference genomes were retained. The transcriptome was analysed for redundancy with CD-HIT software with 95% identity to make a nonredundant set [92, 93]. Functional annotation was carried out on a local server using BLASTx and the nr (NCBI non-redundant protein 12/2018 version) database. Searches were limited to the first 20 significant results with an E-cutoff value of 1.0E-6. Unitigs were functionally annotated utilizing default annotation rules in the BLAST2GO package [94]. The unitigs and their BLASTX results were imported into the BLAST2GO package for functional gene annotation [94]. Gene ontology (GO) term and functional annotation assignments followed InterPro scan, using the European Molecular Biology Laboratory-European Bioinformatics Institute (EMBL-EBI) database, KEGG pathway analysis [95], Rfam annotation [96], and GO mapping based characterizations online on the BLAST2GO package [97].

### Differentially expressed gene (DEG) and gene ontology (GO) enrichment analysis

Quality trimmed and filtered reads from all samples were mapped to the final transcriptome assembly with default parameters in the CLC – GWB. Statistical tests for the determination of differential gene expression utilize an exact test-like generalized linear model (GLM) similar to that performed in DESeq and EdgeR [98, 99]. In developing inflorescences, the test of differential expression utilized non-flowering controls for comparison. The statistical tests for differential expression in developing floral spikelets utilized two or more pair-wise comparisons between developing inflorescences and the sample query (e.g., FT, PAF, ST, ANT). Two or more pair-wise statistical tests between inflorescence controls, floral samples, and developing seeds comprise DEG calls. Genes of interest were filtered from differentially expressed genes table in CLC-GWB using a threshold *p* ≤ 0.05 and a two-fold threshold change. Venn diagrams were generated from gene lists using InteractiVenn [100]. TPM normalized expression values for DEG’s presented in each Venn diagram (Figs. 2, 4, and 6) were subjected coexpression analysis utilizing the R package “MBCluster.Seq” (version 1.0) [101]. Negative binomial modeling parameters were strategically adjusted to evaluate the number of coexpression clusters. Probability estimates greater than 0.9 for each member of a pair of genes coexpressing within the same cluster, as the parameters were adjusted, were used in the progressive selection of the clustering strategy as well as their gene memberships (Supplementary Fig. 5). The final clustering utilized a reduced set of expression data containing the listing of genes from the most probable coexpressing gene set identified within the preliminary analyses. Individual clusters were analyzed independently in CLC-GWB with the hierachical clustering algorithm in the development of the heat maps presented in Figs. 3, 5, and 7.

### Gene expression analysis by RT-qPCR

Bioinformatically derived differential expression statistics of inflorescence, floret, and seed development were screened for novel and putative genes in reproductive development to validate the sample set with RT-qPCR according to Zhao et al. [102]. GO enrichment by hypergeometric test (*p* ≤ 0.05; CLC-GWB) aided in selecting sequences from the test set for over-representation (Supplementary Table 2, 3, & 4). Unigenes were filtered for significant differential expression (FDR *p* ≤ 0.05) within the subset of relevant inflorescence samples. Final transcript selections were made on the unique mapping (GMAP; V2015-07-23, [89]) of the transcript to the reference genome assembly with concomitant support for gene architecture from the PacBio Iso-Seq data set. Primers were designed for each gene to maximize coverage for gene structures, which uniquely identified the isoform of interest. Multiple internal controls were selected from the RNA-seq data set by filtering the expression data set for unigenes with a minimum expression value of 200 transcripts per million (TPM), a mean value of less than 2000 TPM, and having a CV less than 0.35 [103]. Relative gene expression analysis was used in the evaluation of PCR data in the determination of gene expression values and calculated following the 2^-∆∆Ct^ method [104].

## Supplementary Information

**Additional file 1: Table S1.** Sequencing statistics. **Figure S1a-c.** Transcriptome assembly. **Figure S2.** Annotation statistics for primary de novo assembly. **Figure S3.** Annotation statistics for cluster enriched assembly. **Figure S4.** Annotation statistics for PB Iso-Seq sequences. **Table S2.** GO-term enrichment for upregulated transcripts during inflorescence development. **Table S3.** GO-term enrichment for upregulated transcripts during flower development. **Table S4.** GO-term enrichment for upregulated transcripts during seed development. **Table S5.** Excel workbook including summaries of DEG’s in inflorescence development. **Table S6.** Excel workbook including summaries of DEG’s in floral development. **Table S7.** Excel workbook including summaries of DEG’s in seed development. **Supplemental List 1.** List of FASTA formatted sequences associated with Fig. 8 and Tables 2, 3, and 4. **Table S8.** Table export of annotations for the cluster enriched de novo transcriptome assembly. **Table S9.** Table export of annotations for the collapsed Iso-seq transcript set.

## Data Availability

The datasets generated and/or analyzed during the current study are available in the NCBI Bioproject repository PRJNA623617, Tripidium ravennae (ID 623617) (https://www.ncbi.nlm.nih.gov/bioproject/PRJNA623617).

## Abbreviations

ANT: Florets at anthesis
CV: Coefficient of variation
DEG: Differentially expressed gene
DTE: Differentially expressed transcript
FT: Boot stage florets
GO: Gene ontology
IS: Florets with immature seeds
MS: Florets with mature seeds
PAF: Pre-anthesis florets
RNA: Ribonucleic acid
RNA-seq: RNA sequence
ST: Stamens
TPM: Transcripts per million
VM: Vegetative meristem
20P: Inflorescence meristem sampled at ~ 20 cm from the base of the culm
40P: Inflorescence meristem sampled at ~ 40 cm from the base of the culm
80P: Inflorescence meristem sampled at ~ 80 cm from the base of the culm
120P: Inflorescence meristem sampled at ~120 cm from the base of the culm
160P: Inflorescence meristem sampled at ~160 cm from the base of the culm
200P: Inflorescence meristem sampled at ~200 cm from the base of the culm
