# Supplementary material for: Reproductive developmental transcriptome analysis of Tripidium ravennae (Poaceae)
Source: BMC Genomics. 2021 Jun 28;22:483. doi: 10.1186/s12864-021-07641-y (PMC8237498; doi:10.1186/s12864-021-07641-y)
Supplement: Supplementary file 1 — Additional file 1: Table S1. Sequencing statistics. Figure S1a-c. Transcriptome assembly. Figure S2. Annotation statistics for primary de novo assembly. Figure S3. Annotation statistics for cluster enriched assembly. Figure S4. Annotation statistics for PB Iso-Seq sequences. Table S2. GO-term enrichment for upregulated transcripts during inflorescence development. Table S3. GO-term enrichment for upregulated transcripts during flower development. Table S4. GO-term enrichment for upregulated transcripts during seed development. Table S5. Excel workbook including summaries of DEG’s in inflorescence development. Table S6. Excel workbook including summaries of DEG’s in floral development. Table S7. Excel workbook including summaries of DEG’s in seed development. Supplemental List 1. List of FASTA formatted sequences associated with Fig. 8 and Tables 2, 3, and 4. Table S8. Table export of annotations for the cluster enriched de novo transcriptome assembly. Table S9. Table export of annotations for the collapsed Iso-seq transcript set. [file 12864_2021_7641_MOESM1_ESM.zip › SF1-TranscriptomeAssembly.pptx]

## Slide 1
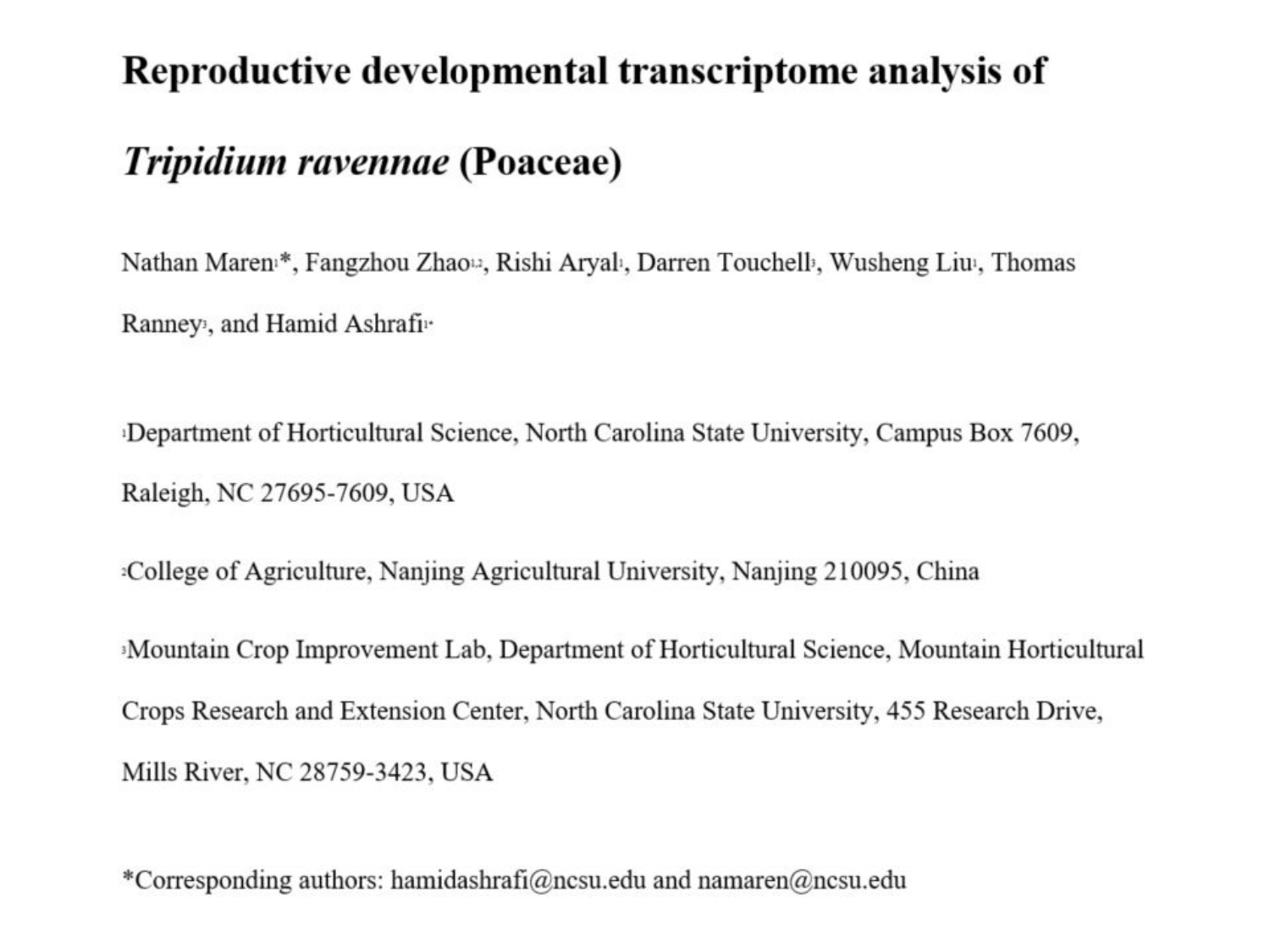

## Slide 2
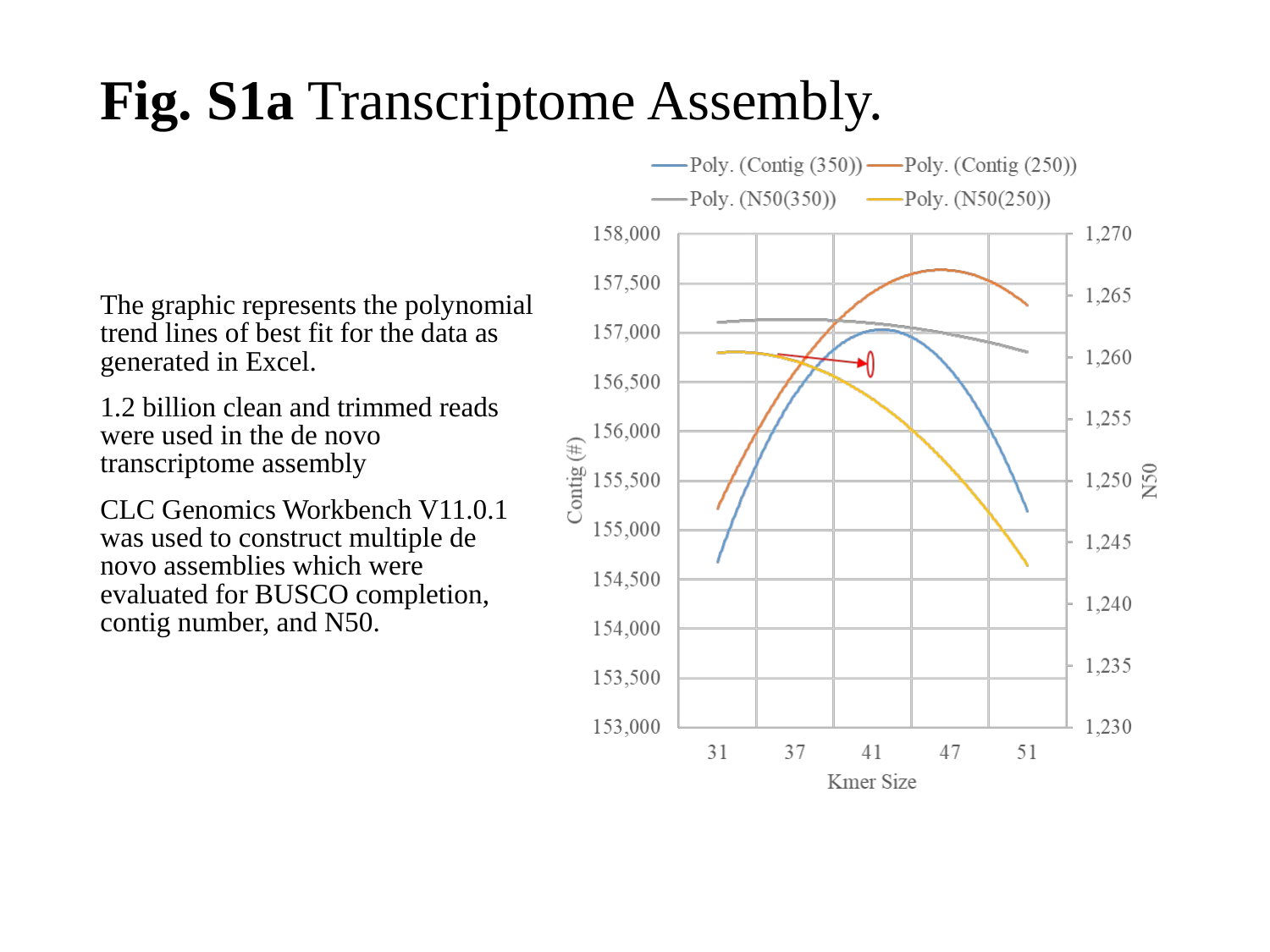

# Fig. S1a Transcriptome Assembly.
### Chart
| Category | | | | |
|---|---|---|---|---|
| 31 | 154679.0 | 155095.0 | 1263.0 | 1261.0 |
| 37 | 156475.0 | 156907.0 | 1262.0 | 1258.0 |
| 41 | 156724.0 | 157257.0 | 1265.0 | 1258.0 |
| 47 | 156927.0 | 157534.0 | 1260.0 | 1251.0 |
| 51 | 155095.0 | 157351.0 | 1261.0 | 1243.0 |
The graphic represents the polynomial trend lines of best fit for the data as generated in Excel.
1.2 billion clean and trimmed reads were used in the de novo transcriptome assembly
CLC Genomics Workbench V11.0.1 was used to construct multiple de novo assemblies which were evaluated for BUSCO completion, contig number, and N50.

## Slide 3
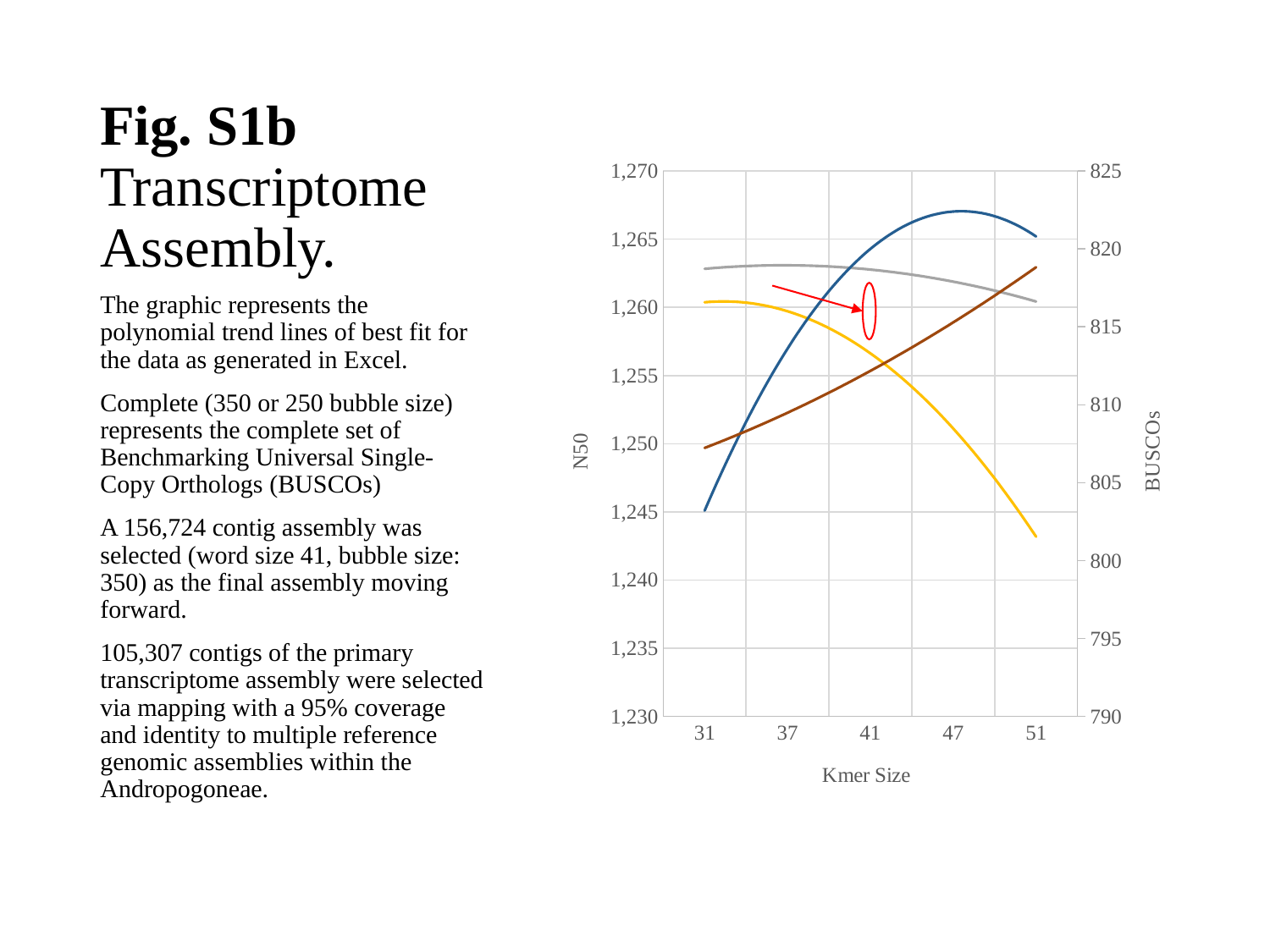

# Fig. S1b Transcriptome Assembly.
### Chart
| Category | | | | |
|---|---|---|---|---|
| 31 | 1263.0 | 1261.0 | 803.0 | 808.0 |
| 37 | 1262.0 | 1258.0 | 814.0 | 807.0 |
| 41 | 1265.0 | 1258.0 | None | 815.0 |
| 47 | 1260.0 | 1251.0 | 822.0 | 814.0 |
| 51 | 1261.0 | 1243.0 | 821.0 | 819.0 |
The graphic represents the polynomial trend lines of best fit for the data as generated in Excel.
Complete (350 or 250 bubble size) represents the complete set of Benchmarking Universal Single-Copy Orthologs (BUSCOs)
A 156,724 contig assembly was selected (word size 41, bubble size: 350) as the final assembly moving forward.
105,307 contigs of the primary transcriptome assembly were selected via mapping with a 95% coverage and identity to multiple reference genomic assemblies within the Andropogoneae.

## Slide 4
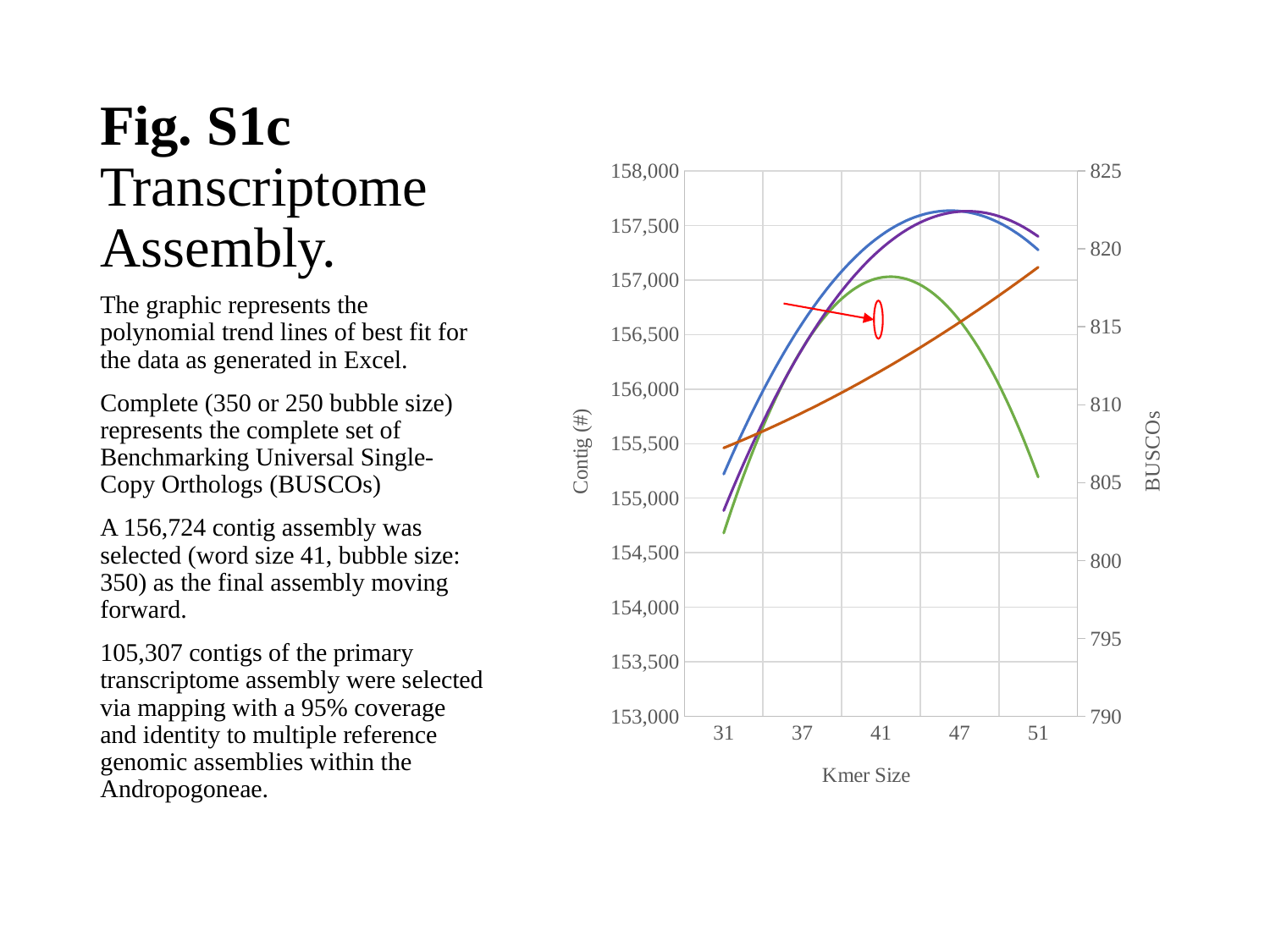

# Fig. S1c Transcriptome Assembly.
### Chart
| Category | | | | |
|---|---|---|---|---|
| 31 | 154679.0 | 155095.0 | 803.0 | 808.0 |
| 37 | 156475.0 | 156907.0 | 814.0 | 807.0 |
| 41 | 156724.0 | 157257.0 | None | 815.0 |
| 47 | 156927.0 | 157534.0 | 822.0 | 814.0 |
| 51 | 155095.0 | 157351.0 | 821.0 | 819.0 |The graphic represents the polynomial trend lines of best fit for the data as generated in Excel.
Complete (350 or 250 bubble size) represents the complete set of Benchmarking Universal Single-Copy Orthologs (BUSCOs)
A 156,724 contig assembly was selected (word size 41, bubble size: 350) as the final assembly moving forward.
105,307 contigs of the primary transcriptome assembly were selected via mapping with a 95% coverage and identity to multiple reference genomic assemblies within the Andropogoneae.

## Slide 5
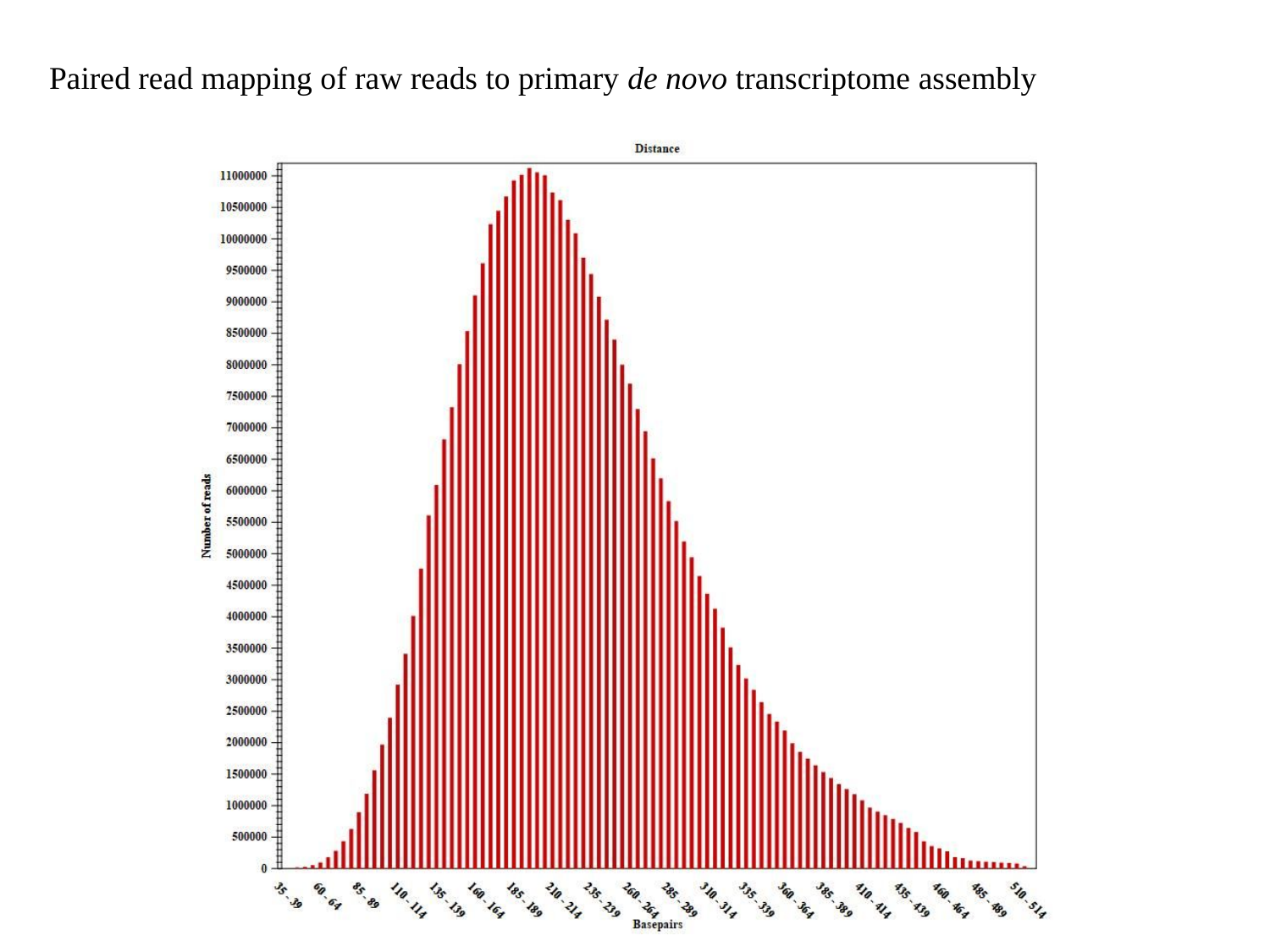

# Paired read mapping of raw reads to primary de novo transcriptome assembly

## Slide 6
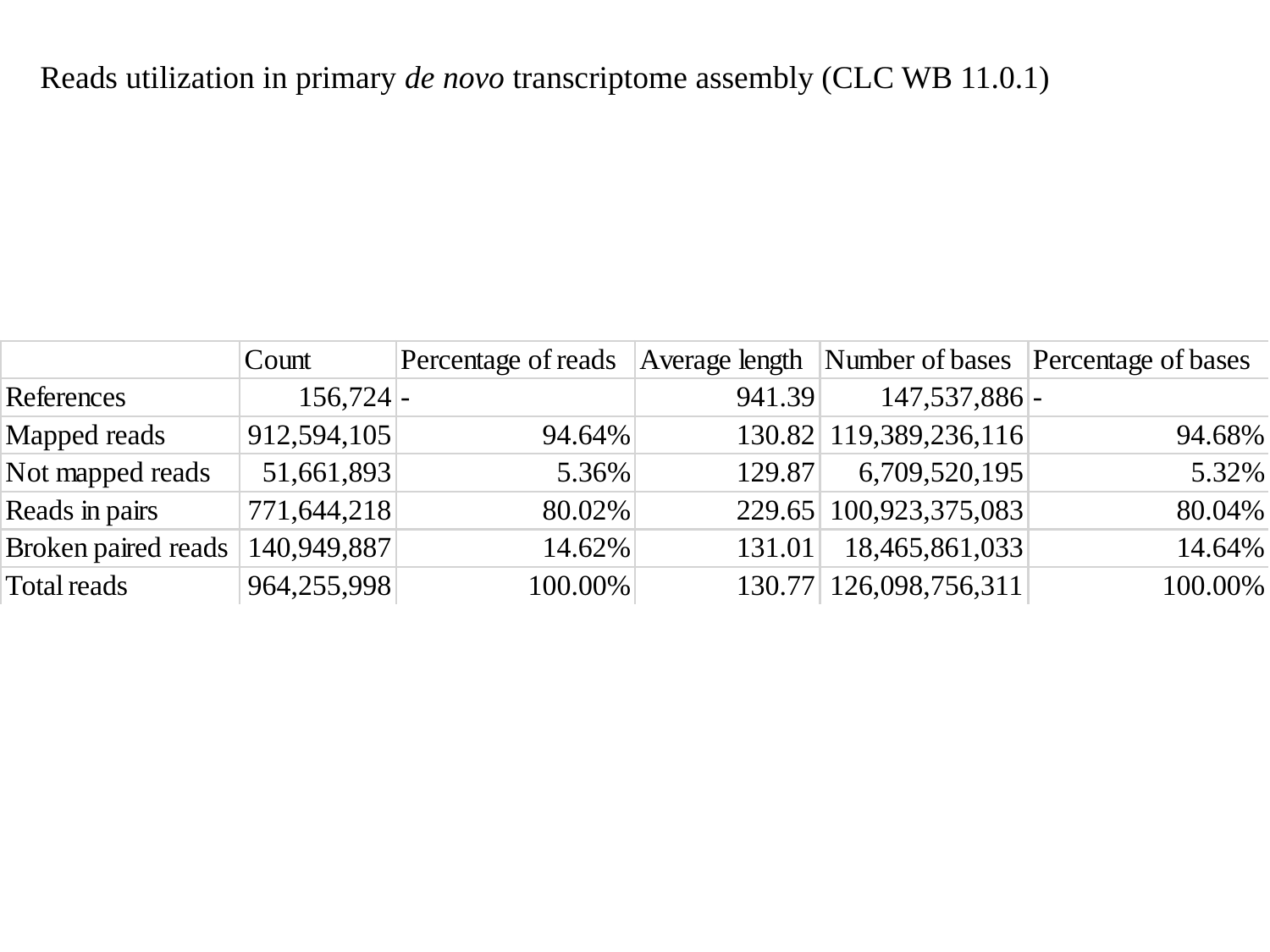

Reads utilization in primary de novo transcriptome assembly (CLC WB 11.0.1)

## Slide 7
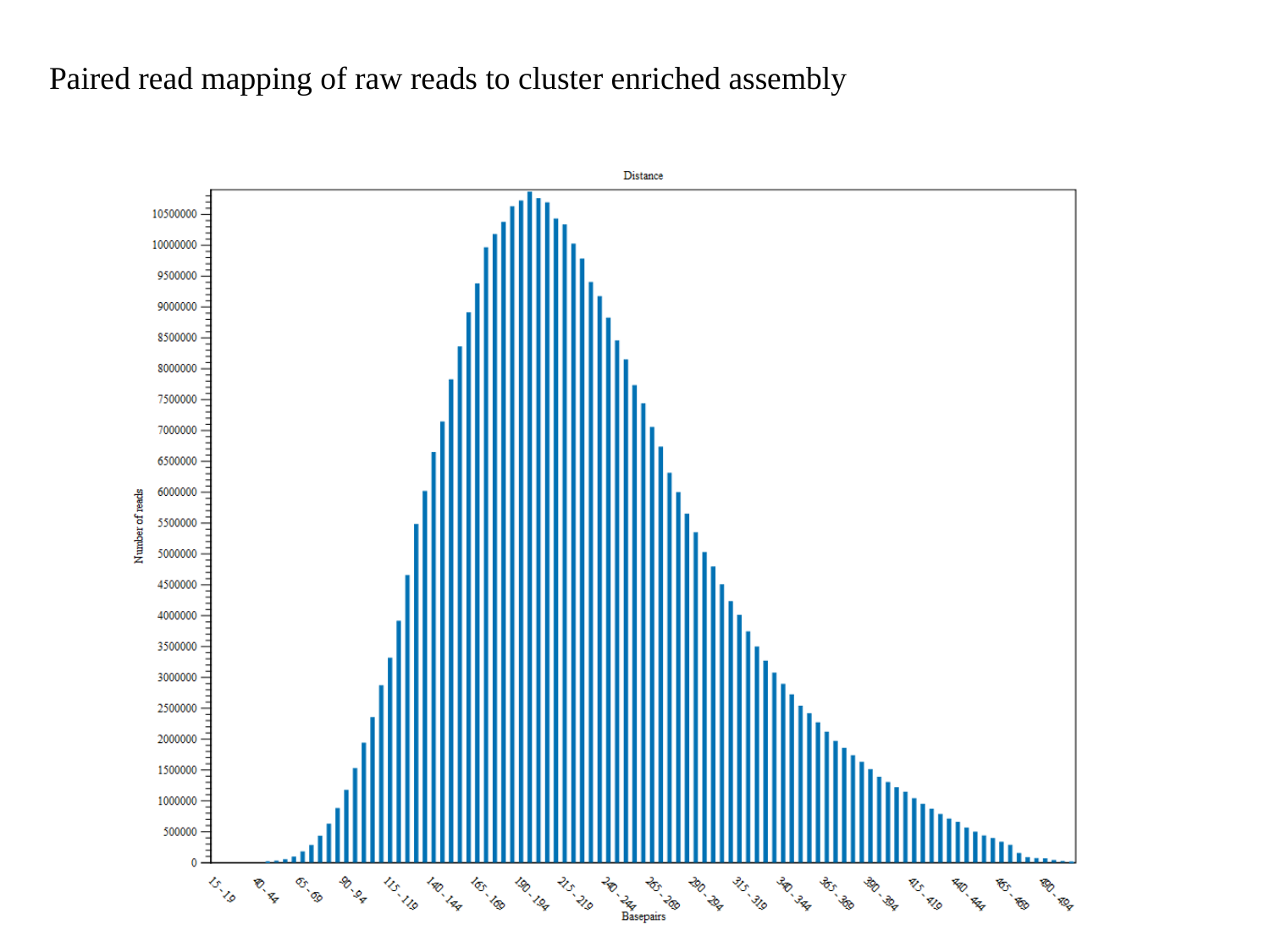

# Paired read mapping of raw reads to cluster enriched assembly

## Slide 8
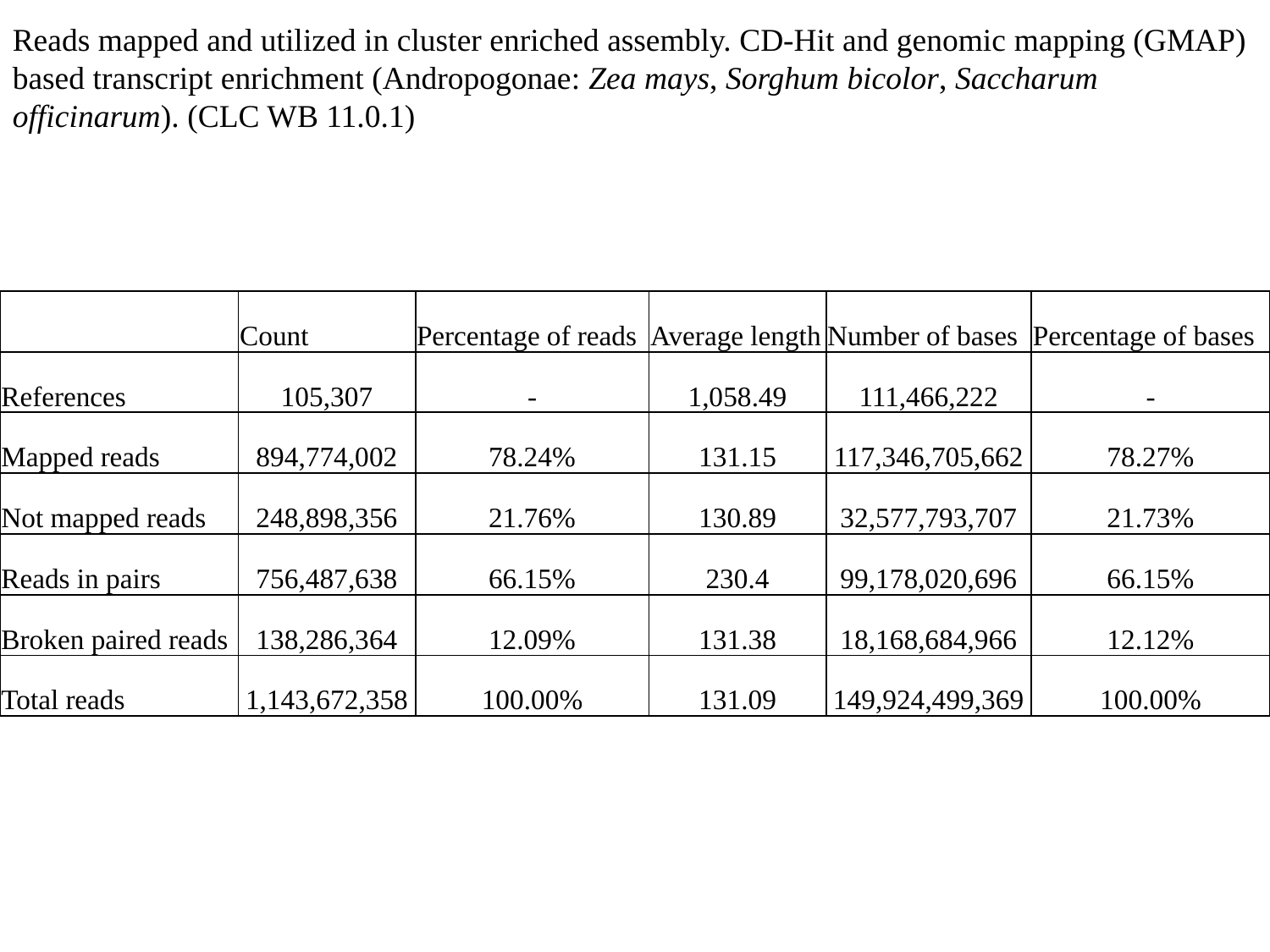

Reads mapped and utilized in cluster enriched assembly. CD-Hit and genomic mapping (GMAP) based transcript enrichment (Andropogonae: Zea mays, Sorghum bicolor, Saccharum officinarum). (CLC WB 11.0.1)
| | Count | Percentage of reads | Average length | Number of bases | Percentage of bases |
| --- | --- | --- | --- | --- | --- |
| References | 105,307 | - | 1,058.49 | 111,466,222 | - |
| Mapped reads | 894,774,002 | 78.24% | 131.15 | 117,346,705,662 | 78.27% |
| Not mapped reads | 248,898,356 | 21.76% | 130.89 | 32,577,793,707 | 21.73% |
| Reads in pairs | 756,487,638 | 66.15% | 230.4 | 99,178,020,696 | 66.15% |
| Broken paired reads | 138,286,364 | 12.09% | 131.38 | 18,168,684,966 | 12.12% |
| Total reads | 1,143,672,358 | 100.00% | 131.09 | 149,924,499,369 | 100.00% |

## Slide 9
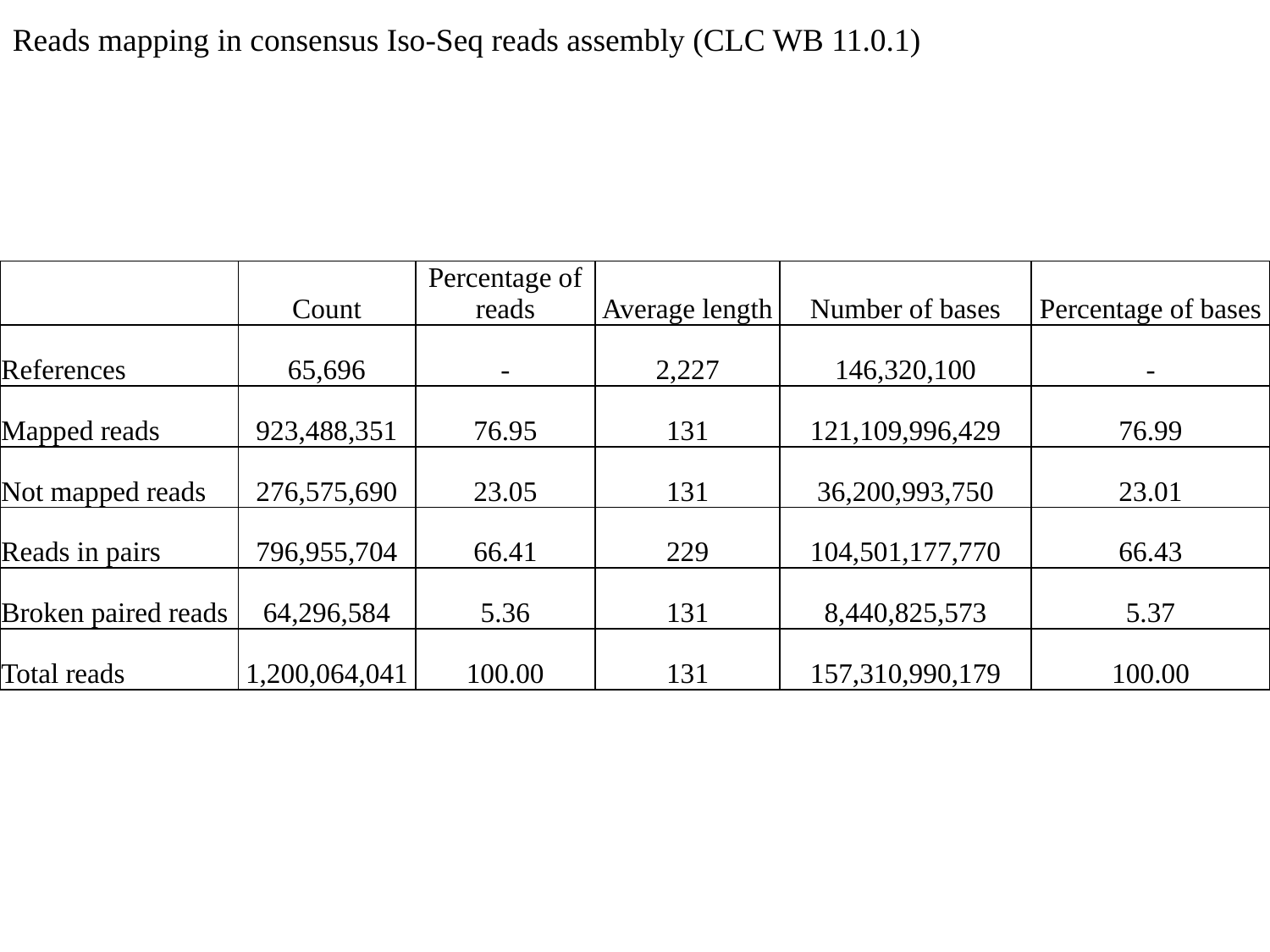

Reads mapping in consensus Iso-Seq reads assembly (CLC WB 11.0.1)
| | Count | Percentage of reads | Average length | Number of bases | Percentage of bases |
| --- | --- | --- | --- | --- | --- |
| References | 65,696 | - | 2,227 | 146,320,100 | - |
| Mapped reads | 923,488,351 | 76.95 | 131 | 121,109,996,429 | 76.99 |
| Not mapped reads | 276,575,690 | 23.05 | 131 | 36,200,993,750 | 23.01 |
| Reads in pairs | 796,955,704 | 66.41 | 229 | 104,501,177,770 | 66.43 |
| Broken paired reads | 64,296,584 | 5.36 | 131 | 8,440,825,573 | 5.37 |
| Total reads | 1,200,064,041 | 100.00 | 131 | 157,310,990,179 | 100.00 |
