# Supplementary material for: Reproductive developmental transcriptome analysis of Tripidium ravennae (Poaceae)
Source: BMC Genomics. 2021 Jun 28;22:483. doi: 10.1186/s12864-021-07641-y (PMC8237498; doi:10.1186/s12864-021-07641-y)
Supplement: Supplementary file 1 — Additional file 1: Table S1. Sequencing statistics. Figure S1a-c. Transcriptome assembly. Figure S2. Annotation statistics for primary de novo assembly. Figure S3. Annotation statistics for cluster enriched assembly. Figure S4. Annotation statistics for PB Iso-Seq sequences. Table S2. GO-term enrichment for upregulated transcripts during inflorescence development. Table S3. GO-term enrichment for upregulated transcripts during flower development. Table S4. GO-term enrichment for upregulated transcripts during seed development. Table S5. Excel workbook including summaries of DEG’s in inflorescence development. Table S6. Excel workbook including summaries of DEG’s in floral development. Table S7. Excel workbook including summaries of DEG’s in seed development. Supplemental List 1. List of FASTA formatted sequences associated with Fig. 8 and Tables 2, 3, and 4. Table S8. Table export of annotations for the cluster enriched de novo transcriptome assembly. Table S9. Table export of annotations for the collapsed Iso-seq transcript set. [file 12864_2021_7641_MOESM1_ESM.zip › ST1-Sequencing_statistics.docx]

**Reproductive developmental transcriptome analysis of *Tripidium ravennae* (Poaceae)**

Nathan Maren^1^*, Fangzhou Zhao^1,2^, Rishi Aryal^1^, Darren Touchell^3^, Wusheng Liu^1^, Thomas Ranney^3^, and Hamid Ashrafi^1*^

^1^Department of Horticultural Science, North Carolina State University, Campus Box 7609, Raleigh, NC 27695-7609, USA

^2^College of Agriculture, Nanjing Agricultural University, Nanjing 210095, China

^3^Mountain Crop Improvement Lab, Department of Horticultural Science, Mountain Horticultural Crops Research and Extension Center, North Carolina State University, 455 Research Drive, Mills River, NC 28759-3423, USA

*Corresponding authors: hamidashrafi@ncsu.edu and namaren@ncsu.edu

**Table S1** Sequencing statistics.

| ID | Tissue | Total raw reads^x^ | Total length of raw reads (Gbp)^y^ | Quality filtered & trimmed reads^x^ | Total length of trimmed reads (Gbp)^y^ |
| --- | --- | --- | --- | --- | --- |
| VM | Vegetative Meristem | 29,812,237 | 8.9 | 25,112,505 | 6.6 |
| 20P | Inflorescence Meristem | 33,699,083 | 10.1 | 29,985,091 | 7.9 |
| 40P | Inflorescence Meristem | 42,503,231 | 12.8 | 36,995,921 | 9.7 |
| 80P | Inflorescence Meristem | 54,240,852 | 16.3 | 49,747,515 | 13.0 |
| 120P | Inflorescence Meristem | 74,347,939 | 22.3 | 67,451,361 | 17.7 |
| 160P | Inflorescence Meristem | 47,337,556 | 14.2 | 42,356,822 | 11.1 |
| 200P | Inflorescence Meristem | 34,575,379 | 10.4 | 32,802,909 | 8.6 |
| FT | Boot Stage Florets | 62,652,702 | 18.8 | 57,474,246 | 15.1 |
| PAF | Pre-Anthesis Florets | 52,425,079 | 15.7 | 48,474,879 | 13.0 |
| ANT | Anthesis Florets | 14,389,965 | 4.3 | 11,658,036 | 3.1 |
| ST | Anthers | 115,394,962 | 34.6 | 102,274,605 | 26.7 |
| IS | Immature Seeds | 44,399,802 | 13.3 | 37,029,960 | 9.7 |
| MS | Mature Seeds | 65,113,926 | 19.5 | 59,258,350 | 15.5 |
| TOTAL |  | 670,892,713 | 201.3 | 601,087,345 | 158.0 |

^x^ Reads are counted as the number of individual reads of data from the sequencer. Paired reads are counted individually.

^y^ Bases are calculated as reads times sequence length.
