# Supplementary material for: Reproductive developmental transcriptome analysis of Tripidium ravennae (Poaceae)
Source: BMC Genomics. 2021 Jun 28;22:483. doi: 10.1186/s12864-021-07641-y (PMC8237498; doi:10.1186/s12864-021-07641-y)
Supplement: Supplementary file 1 — Additional file 1: Table S1. Sequencing statistics. Figure S1a-c. Transcriptome assembly. Figure S2. Annotation statistics for primary de novo assembly. Figure S3. Annotation statistics for cluster enriched assembly. Figure S4. Annotation statistics for PB Iso-Seq sequences. Table S2. GO-term enrichment for upregulated transcripts during inflorescence development. Table S3. GO-term enrichment for upregulated transcripts during flower development. Table S4. GO-term enrichment for upregulated transcripts during seed development. Table S5. Excel workbook including summaries of DEG’s in inflorescence development. Table S6. Excel workbook including summaries of DEG’s in floral development. Table S7. Excel workbook including summaries of DEG’s in seed development. Supplemental List 1. List of FASTA formatted sequences associated with Fig. 8 and Tables 2, 3, and 4. Table S8. Table export of annotations for the cluster enriched de novo transcriptome assembly. Table S9. Table export of annotations for the collapsed Iso-seq transcript set. [file 12864_2021_7641_MOESM1_ESM.zip › ST2-GOenrichmentAnalysisInflorDvlpmt.docx]

**Reproductive developmental transcriptome analysis of *Tripidium ravennae* (Poaceae)**

Nathan Maren^1^*, Fangzhou Zhao^1,2^, Rishi Aryal^1^, Darren Touchell^3^, Wusheng Liu^1^, Thomas Ranney^3^, and Hamid Ashrafi^1*^

^1^Department of Horticultural Science, North Carolina State University, Campus Box 7609, Raleigh, NC 27695-7609, USA

^2^College of Agriculture, Nanjing Agricultural University, Nanjing 210095, China

^3^Mountain Crop Improvement Lab, Department of Horticultural Science, Mountain Horticultural Crops Research and Extension Center, North Carolina State University, 455 Research Drive, Mills River, NC 28759-3423, USA

*Corresponding authors: hamidashrafi@ncsu.edu and namaren@ncsu.edu

| **Category** | **Description** | **Full set** | **In subset** | **Expected in subset** | **Observed - expected** | **p-value** |
| --- | --- | --- | --- | --- | --- | --- |
| 0005975 | carbohydrate metabolic process | 1389 | 412 | 263 | 149 | 0.00 |
| 0044699 | single-organism process | 9115 | 2098 | 1723 | 375 | 0.00 |
| 0044710 | single-organism metabolic process | 5645 | 1325 | 1067 | 258 | 0.00 |
| 0045229 | external encapsulating structure organization | 349 | 145 | 66 | 79 | 0.00 |
| 0071555 | cell wall organization | 332 | 140 | 63 | 77 | 0.00 |
| 0071554 | cell wall organization or biogenesis | 374 | 159 | 71 | 88 | 0.00 |
| 0055114 | oxidation-reduction process | 2524 | 640 | 477 | 163 | 1.11E-16 |
| 0044763 | single-organism cellular process | 5600 | 1250 | 1058 | 192 | 1.53E-13 |
| 0016051 | carbohydrate biosynthetic process | 361 | 123 | 68 | 55 | 4.54E-12 |
| 0044723 | single-organism carbohydrate metabolic process | 755 | 219 | 143 | 76 | 5.81E-12 |
| 0044262 | cellular carbohydrate metabolic process | 525 | 161 | 99 | 62 | 4.09E-11 |
| 0007017 | microtubule-based process | 269 | 96 | 51 | 45 | 5.54E-11 |
| 0044711 | single-organism biosynthetic process | 1908 | 468 | 361 | 107 | 1.41E-10 |
| 0042546 | cell wall biogenesis | 133 | 57 | 25 | 32 | 1.71E-10 |
| 0005976 | polysaccharide metabolic process | 477 | 147 | 90 | 57 | 1.95E-10 |
| 0044703 | multi-organism reproductive process | 39 | 25 | 7 | 18 | 7.19E-10 |
| 0007018 | microtubule-based movement | 108 | 48 | 20 | 28 | 1.05E-9 |
| 0019953 | sexual reproduction | 37 | 23 | 7 | 16 | 8.31E-9 |
| 1901700 | response to oxygen-containing compound | 519 | 151 | 98 | 53 | 9.07E-9 |
| 0006928 | movement of cell or subcellular component | 121 | 50 | 23 | 27 | 9.67E-9 |
| 0042221 | response to chemical | 896 | 237 | 169 | 68 | 1.06E-8 |
| 0007165 | signal transduction | 1357 | 338 | 256 | 82 | 1.13E-8 |
| 0006091 | generation of precursor metabolites and energy | 483 | 142 | 91 | 51 | 1.21E-8 |
| 0009628 | response to abiotic stimulus | 885 | 234 | 167 | 67 | 1.36E-8 |
| 0044264 | cellular polysaccharide metabolic process | 326 | 102 | 62 | 40 | 4.80E-8 |
| 0034637 | cellular carbohydrate biosynthetic process | 266 | 87 | 50 | 37 | 5.01E-8 |
| 0000003 | reproduction | 40 | 23 | 8 | 15 | 6.67E-8 |
| 0001101 | response to acid chemical | 365 | 111 | 69 | 42 | 6.80E-8 |
| 0032787 | monocarboxylic acid metabolic process | 571 | 159 | 108 | 51 | 9.17E-8 |
| 0046942 | carboxylic acid transport | 184 | 65 | 35 | 30 | 1.02E-7 |
| 0015849 | organic acid transport | 184 | 65 | 35 | 30 | 1.02E-7 |
| 0044042 | glucan metabolic process | 286 | 91 | 54 | 37 | 1.06E-7 |
| 0006073 | cellular glucan metabolic process | 274 | 88 | 52 | 36 | 1.07E-7 |
| 0044712 | single-organism catabolic process | 706 | 189 | 133 | 56 | 1.32E-7 |
| 0006811 | ion transport | 1269 | 312 | 240 | 72 | 1.60E-7 |
| 0050789 | regulation of biological process | 5189 | 1110 | 981 | 129 | 2.00E-7 |
| 0042744 | hydrogen peroxide catabolic process | 154 | 56 | 29 | 27 | 2.55E-7 |
| 0051704 | multi-organism process | 473 | 134 | 89 | 45 | 3.25E-7 |
| 0055085 | transmembrane transport | 2005 | 465 | 379 | 86 | 3.33E-7 |
| 0009832 | plant-type cell wall biogenesis | 91 | 38 | 17 | 21 | 4.01E-7 |
| 0042743 | hydrogen peroxide metabolic process | 156 | 56 | 29 | 27 | 4.16E-7 |
| 0006820 | anion transport | 463 | 131 | 88 | 43 | 4.72E-7 |
| 0006629 | lipid metabolic process | 1355 | 327 | 256 | 71 | 5.28E-7 |
| 0000271 | polysaccharide biosynthetic process | 238 | 76 | 45 | 31 | 9.75E-7 |
| 0071669 | plant-type cell wall organization or biogenesis | 94 | 38 | 18 | 20 | 1.06E-6 |
| 1903825 | organic acid transmembrane transport | 134 | 49 | 25 | 24 | 1.15E-6 |
| 1905039 | carboxylic acid transmembrane transport | 134 | 49 | 25 | 24 | 1.15E-6 |
| 0098656 | anion transmembrane transport | 240 | 76 | 45 | 31 | 1.40E-6 |
| 0015711 | organic anion transport | 314 | 94 | 59 | 35 | 1.42E-6 |
| 0050794 | regulation of cellular process | 4745 | 1011 | 897 | 114 | 1.84E-6 |
| 0010383 | cell wall polysaccharide metabolic process | 159 | 55 | 30 | 25 | 2.00E-6 |
| 0009768 | photosynthesis, light harvesting in photosystem I | 21 | 14 | 4 | 10 | 2.20E-6 |
| 0022402 | cell cycle process | 399 | 112 | 75 | 37 | 4.63E-6 |
| 0009765 | photosynthesis, light harvesting | 22 | 14 | 4 | 10 | 5.00E-6 |
| 0003333 | amino acid transmembrane transport | 122 | 44 | 23 | 21 | 6.00E-6 |
| 0051301 | cell division | 141 | 49 | 27 | 22 | 6.09E-6 |
| 0044281 | small molecule metabolic process | 2086 | 471 | 394 | 77 | 6.34E-6 |
| 0006631 | fatty acid metabolic process | 258 | 78 | 49 | 29 | 7.19E-6 |
| 0051783 | regulation of nuclear division | 84 | 33 | 16 | 17 | 1.10E-5 |
| 0010410 | hemicellulose metabolic process | 140 | 48 | 26 | 22 | 1.14E-5 |
| 0010033 | response to organic substance | 526 | 139 | 99 | 40 | 1.19E-5 |
| 0007010 | cytoskeleton organization | 176 | 57 | 33 | 24 | 1.31E-5 |
| 0090627 | plant epidermal cell differentiation | 16 | 11 | 3 | 8 | 1.85E-5 |
| 0072593 | reactive oxygen species metabolic process | 186 | 59 | 35 | 24 | 1.88E-5 |
| 0008610 | lipid biosynthetic process | 703 | 177 | 133 | 44 | 1.88E-5 |
| 0015979 | photosynthesis | 97 | 36 | 18 | 18 | 1.98E-5 |
| 0044085 | cellular component biogenesis | 236 | 71 | 45 | 26 | 2.16E-5 |
| 0008284 | positive regulation of cell proliferation | 55 | 24 | 10 | 14 | 2.21E-5 |
| 0050896 | response to stimulus | 2995 | 649 | 566 | 83 | 2.65E-5 |
| 0071705 | nitrogen compound transport | 530 | 138 | 100 | 38 | 2.79E-5 |
| 0009734 | auxin-activated signaling pathway | 95 | 35 | 18 | 17 | 3.08E-5 |
| 1901615 | organic hydroxy compound metabolic process | 315 | 89 | 60 | 29 | 3.20E-5 |
| 0065007 | biological regulation | 5969 | 1235 | 1128 | 107 | 3.20E-5 |
| 0030243 | cellulose metabolic process | 103 | 37 | 19 | 18 | 3.52E-5 |
| 0006865 | amino acid transport | 130 | 44 | 25 | 19 | 3.70E-5 |
| 0009416 | response to light stimulus | 282 | 81 | 53 | 28 | 3.79E-5 |
| 0009753 | response to jasmonic acid | 37 | 18 | 7 | 11 | 3.98E-5 |
| 0009834 | plant-type secondary cell wall biogenesis | 50 | 22 | 9 | 13 | 4.12E-5 |
| 1901617 | organic hydroxy compound biosynthetic process | 195 | 60 | 37 | 23 | 4.40E-5 |
| 0000226 | microtubule cytoskeleton organization | 147 | 48 | 28 | 20 | 4.80E-5 |
| 0044036 | cell wall macromolecule metabolic process | 204 | 62 | 39 | 23 | 4.98E-5 |
| 0022900 | electron transport chain | 293 | 83 | 55 | 28 | 5.25E-5 |
| 0007167 | enzyme linked receptor protein signaling pathway | 276 | 79 | 52 | 27 | 5.35E-5 |
| 0007178 | transmembrane receptor protein serine/threonine kinase signaling pathway | 276 | 79 | 52 | 27 | 5.35E-5 |
| 0034220 | ion transmembrane transport | 871 | 210 | 165 | 45 | 6.07E-5 |
| 0045489 | pectin biosynthetic process | 36 | 17 | 7 | 10 | 1.04E-4 |
| 0009719 | response to endogenous stimulus | 422 | 111 | 80 | 31 | 1.05E-4 |
| 0045787 | positive regulation of cell cycle | 85 | 31 | 16 | 15 | 1.06E-4 |
| 0006270 | DNA replication initiation | 33 | 16 | 6 | 10 | 1.12E-4 |
| 0015718 | monocarboxylic acid transport | 33 | 16 | 6 | 10 | 1.12E-4 |
| 0051273 | beta-glucan metabolic process | 124 | 41 | 23 | 18 | 1.21E-4 |
| 0009620 | response to fungus | 132 | 43 | 25 | 18 | 1.23E-4 |
| 0033692 | cellular polysaccharide biosynthetic process | 210 | 62 | 40 | 22 | 1.25E-4 |
| 0009314 | response to radiation | 300 | 83 | 57 | 26 | 1.27E-4 |
| 0009605 | response to external stimulus | 565 | 142 | 107 | 35 | 1.31E-4 |
| 0007264 | small GTPase mediated signal transduction | 75 | 28 | 14 | 14 | 1.41E-4 |
| 0044282 | small molecule catabolic process | 288 | 80 | 54 | 26 | 1.44E-4 |
| 0010035 | response to inorganic substance | 376 | 100 | 71 | 29 | 1.44E-4 |
| 0009414 | response to water deprivation | 137 | 44 | 26 | 18 | 1.49E-4 |
| 0015781 | pyrimidine nucleotide-sugar transport | 47 | 20 | 9 | 11 | 1.60E-4 |
| 0098869 | cellular oxidant detoxification | 250 | 71 | 47 | 24 | 1.61E-4 |
| 0007049 | cell cycle | 61 | 24 | 12 | 12 | 1.63E-4 |
| 0048869 | cellular developmental process | 311 | 85 | 59 | 26 | 1.66E-4 |
| 0006979 | response to oxidative stress | 316 | 86 | 60 | 26 | 1.78E-4 |
| 0009725 | response to hormone | 392 | 103 | 74 | 29 | 1.89E-4 |
| 0051179 | localization | 3732 | 785 | 705 | 80 | 1.93E-4 |
| 0046351 | disaccharide biosynthetic process | 51 | 21 | 10 | 11 | 1.94E-4 |
| 0030244 | cellulose biosynthetic process | 69 | 26 | 13 | 13 | 2.03E-4 |
| 0009415 | response to water | 139 | 44 | 26 | 18 | 2.14E-4 |
| 0042631 | cellular response to water deprivation | 9 | 7 | 2 | 5 | 2.16E-4 |
| 0071462 | cellular response to water stimulus | 9 | 7 | 2 | 5 | 2.16E-4 |
| 0007088 | regulation of mitotic nuclear division | 73 | 27 | 14 | 13 | 2.21E-4 |
| 0010411 | xyloglucan metabolic process | 77 | 28 | 15 | 13 | 2.37E-4 |
| 0009312 | oligosaccharide biosynthetic process | 77 | 28 | 15 | 13 | 2.37E-4 |
| 0046717 | acid secretion | 5 | 5 | 1 | 4 | 2.41E-4 |
| 0071715 | icosanoid transport | 5 | 5 | 1 | 4 | 2.41E-4 |
| 1903963 | arachidonate transport | 5 | 5 | 1 | 4 | 2.41E-4 |
| 1901571 | fatty acid derivative transport | 5 | 5 | 1 | 4 | 2.41E-4 |
| 0015909 | long-chain fatty acid transport | 5 | 5 | 1 | 4 | 2.41E-4 |
| 0050482 | arachidonic acid secretion | 5 | 5 | 1 | 4 | 2.41E-4 |
| 0032309 | icosanoid secretion | 5 | 5 | 1 | 4 | 2.41E-4 |
| 0042127 | regulation of cell proliferation | 66 | 25 | 12 | 13 | 2.43E-4 |
| 1903047 | mitotic cell cycle process | 177 | 53 | 33 | 20 | 2.54E-4 |
| 0018298 | protein-chromophore linkage | 35 | 16 | 7 | 9 | 2.66E-4 |
| 0019319 | hexose biosynthetic process | 35 | 16 | 7 | 9 | 2.66E-4 |
| 0051262 | protein tetramerization | 17 | 10 | 3 | 7 | 3.03E-4 |
| 0098754 | detoxification | 260 | 72 | 49 | 23 | 3.30E-4 |
| 0006323 | DNA packaging | 26 | 13 | 5 | 8 | 3.36E-4 |
| 0071214 | cellular response to abiotic stimulus | 71 | 26 | 13 | 13 | 3.44E-4 |
| 0030261 | chromosome condensation | 23 | 12 | 4 | 8 | 3.44E-4 |
| 0006108 | malate metabolic process | 23 | 12 | 4 | 8 | 3.44E-4 |
| 1990748 | cellular detoxification | 256 | 71 | 48 | 23 | 3.45E-4 |
| 0009056 | catabolic process | 1969 | 430 | 372 | 58 | 3.54E-4 |
| 0007051 | spindle organization | 75 | 27 | 14 | 13 | 3.67E-4 |
| 1904666 | regulation of ubiquitin protein ligase activity | 12 | 8 | 2 | 6 | 3.86E-4 |
| 0010052 | guard cell differentiation | 12 | 8 | 2 | 6 | 3.86E-4 |
| 0009755 | hormone-mediated signaling pathway | 341 | 90 | 64 | 26 | 3.95E-4 |
| 0015780 | nucleotide-sugar transport | 57 | 22 | 11 | 11 | 4.13E-4 |
| 0016126 | sterol biosynthetic process | 57 | 22 | 11 | 11 | 4.13E-4 |
| 0006082 | organic acid metabolic process | 1340 | 301 | 253 | 48 | 4.44E-4 |
| 0051234 | establishment of localization | 3597 | 753 | 680 | 73 | 4.66E-4 |
| 0005996 | monosaccharide metabolic process | 233 | 65 | 44 | 21 | 5.04E-4 |
| 0051289 | protein homotetramerization | 15 | 9 | 3 | 6 | 5.04E-4 |
| 0009739 | response to gibberellin | 15 | 9 | 3 | 6 | 5.04E-4 |
| 0006090 | pyruvate metabolic process | 144 | 44 | 27 | 17 | 5.05E-4 |
| 0009694 | jasmonic acid metabolic process | 47 | 19 | 9 | 10 | 5.10E-4 |
| 0019318 | hexose metabolic process | 186 | 54 | 35 | 19 | 5.17E-4 |
| 0090698 | post-embryonic plant morphogenesis | 27 | 13 | 5 | 8 | 5.37E-4 |
| 1901658 | glycosyl compound catabolic process | 24 | 12 | 5 | 7 | 5.70E-4 |
| 0051707 | response to other organism | 381 | 98 | 72 | 26 | 5.88E-4 |
| 0006633 | fatty acid biosynthetic process | 170 | 50 | 32 | 18 | 5.89E-4 |
| 0019752 | carboxylic acid metabolic process | 1297 | 291 | 245 | 46 | 5.93E-4 |
| 1902578 | single-organism localization | 1632 | 359 | 308 | 51 | 6.43E-4 |
| 0009250 | glucan biosynthetic process | 158 | 47 | 30 | 17 | 6.44E-4 |
| 1901565 | organonitrogen compound catabolic process | 209 | 59 | 40 | 19 | 6.44E-4 |
| 0006094 | gluconeogenesis | 34 | 15 | 6 | 9 | 6.57E-4 |
| 0006810 | transport | 3568 | 745 | 674 | 71 | 6.70E-4 |
| 0030001 | metal ion transport | 360 | 93 | 68 | 25 | 6.87E-4 |
| 0009607 | response to biotic stimulus | 415 | 105 | 78 | 27 | 7.17E-4 |
| 0005992 | trehalose biosynthetic process | 31 | 14 | 6 | 8 | 7.44E-4 |
| 0016052 | carbohydrate catabolic process | 379 | 97 | 72 | 25 | 7.47E-4 |
| 0043436 | oxoacid metabolic process | 1337 | 298 | 253 | 45 | 7.91E-4 |
| 0051338 | regulation of transferase activity | 202 | 57 | 38 | 19 | 7.96E-4 |
| 0051274 | beta-glucan biosynthetic process | 90 | 30 | 17 | 13 | 7.99E-4 |
| 0046148 | pigment biosynthetic process | 114 | 36 | 22 | 14 | 8.05E-4 |
| 0007346 | regulation of mitotic cell cycle | 144 | 43 | 27 | 16 | 9.79E-4 |
| 0045488 | pectin metabolic process | 91 | 30 | 17 | 13 | 9.80E-4 |
| 0010393 | galacturonan metabolic process | 91 | 30 | 17 | 13 | 9.80E-4 |
| 0005984 | disaccharide metabolic process | 100 | 32 | 19 | 13 | 1.19E-3 |
| 0009944 | polarity specification of adaxial/abaxial axis | 6 | 5 | 1 | 4 | 1.22E-3 |
| 0007265 | Ras protein signal transduction | 69 | 24 | 13 | 11 | 1.32E-3 |
| 0006812 | cation transport | 752 | 175 | 142 | 33 | 1.39E-3 |
| 0000079 | regulation of cyclin-dependent protein serine/threonine kinase activity | 85 | 28 | 16 | 12 | 1.44E-3 |
| 1904029 | regulation of cyclin-dependent protein kinase activity | 85 | 28 | 16 | 12 | 1.44E-3 |
| 0043207 | response to external biotic stimulus | 391 | 98 | 74 | 24 | 1.46E-3 |
| 0071229 | cellular response to acid chemical | 58 | 21 | 11 | 10 | 1.46E-3 |
| 0072523 | purine-containing compound catabolic process | 23 | 11 | 4 | 7 | 1.54E-3 |
| 0044283 | small molecule biosynthetic process | 754 | 175 | 143 | 32 | 1.57E-3 |
| 0016125 | sterol metabolic process | 118 | 36 | 22 | 14 | 1.60E-3 |
| 0044255 | cellular lipid metabolic process | 981 | 222 | 185 | 37 | 1.62E-3 |
| 0015786 | UDP-glucose transport | 17 | 9 | 3 | 6 | 1.70E-3 |
| 0044765 | single-organism transport | 1561 | 340 | 295 | 45 | 1.73E-3 |
| 0019220 | regulation of phosphate metabolic process | 248 | 66 | 47 | 19 | 1.74E-3 |
| 0051174 | regulation of phosphorus metabolic process | 248 | 66 | 47 | 19 | 1.74E-3 |
| 0030154 | cell differentiation | 165 | 47 | 31 | 16 | 1.76E-3 |
| 0046364 | monosaccharide biosynthetic process | 44 | 17 | 8 | 9 | 1.79E-3 |
| 0042044 | fluid transport | 30 | 13 | 6 | 7 | 1.82E-3 |
| 0006833 | water transport | 30 | 13 | 6 | 7 | 1.82E-3 |
| 0042325 | regulation of phosphorylation | 200 | 55 | 38 | 17 | 1.84E-3 |
| 0006002 | fructose 6-phosphate metabolic process | 37 | 15 | 7 | 8 | 1.87E-3 |
| 0033993 | response to lipid | 192 | 53 | 36 | 17 | 2.02E-3 |
| 0051128 | regulation of cellular component organization | 354 | 89 | 67 | 22 | 2.12E-3 |
| 0071482 | cellular response to light stimulus | 41 | 16 | 8 | 8 | 2.15E-3 |
| 0031399 | regulation of protein modification process | 277 | 72 | 52 | 20 | 2.17E-3 |
| 0072521 | purine-containing compound metabolic process | 318 | 81 | 60 | 21 | 2.22E-3 |
| 0015976 | carbon utilization | 9 | 6 | 2 | 4 | 2.25E-3 |
| 0009409 | response to cold | 163 | 46 | 31 | 15 | 2.40E-3 |
| 0006165 | nucleoside diphosphate phosphorylation | 129 | 38 | 24 | 14 | 2.43E-3 |
| 0043094 | cellular metabolic compound salvage | 64 | 22 | 12 | 10 | 2.45E-3 |
| 0009698 | phenylpropanoid metabolic process | 96 | 30 | 18 | 12 | 2.51E-3 |
| 0009179 | purine ribonucleoside diphosphate metabolic process | 121 | 36 | 23 | 13 | 2.59E-3 |
| 0009135 | purine nucleoside diphosphate metabolic process | 121 | 36 | 23 | 13 | 2.59E-3 |
| 0019748 | secondary metabolic process | 155 | 44 | 29 | 15 | 2.60E-3 |
| 0010564 | regulation of cell cycle process | 168 | 47 | 32 | 15 | 2.62E-3 |
| 0030522 | intracellular receptor signaling pathway | 12 | 7 | 2 | 5 | 2.77E-3 |
| 0009785 | blue light signaling pathway | 12 | 7 | 2 | 5 | 2.77E-3 |
| 1901264 | carbohydrate derivative transport | 109 | 33 | 21 | 12 | 2.81E-3 |
| 0001932 | regulation of protein phosphorylation | 186 | 51 | 35 | 16 | 2.81E-3 |
| 0006694 | steroid biosynthetic process | 130 | 38 | 25 | 13 | 2.82E-3 |
| 0009691 | cytokinin biosynthetic process | 18 | 9 | 3 | 6 | 2.83E-3 |
| 0009164 | nucleoside catabolic process | 18 | 9 | 3 | 6 | 2.83E-3 |
| 0019685 | photosynthesis, dark reaction | 15 | 8 | 3 | 5 | 2.92E-3 |
| 0019253 | reductive pentose-phosphate cycle | 15 | 8 | 3 | 5 | 2.92E-3 |
| 0008643 | carbohydrate transport | 204 | 55 | 39 | 16 | 2.95E-3 |
| 0048285 | organelle fission | 89 | 28 | 17 | 11 | 3.08E-3 |
| 0010167 | response to nitrate | 46 | 17 | 9 | 8 | 3.12E-3 |
| 0006749 | glutathione metabolic process | 131 | 38 | 25 | 13 | 3.26E-3 |
| 0009116 | nucleoside metabolic process | 318 | 80 | 60 | 20 | 3.36E-3 |
| 0042278 | purine nucleoside metabolic process | 250 | 65 | 47 | 18 | 3.42E-3 |
| 0031023 | microtubule organizing center organization | 25 | 11 | 5 | 6 | 3.51E-3 |
| 0072330 | monocarboxylic acid biosynthetic process | 255 | 66 | 48 | 18 | 3.59E-3 |
| 0065001 | specification of axis polarity | 7 | 5 | 1 | 4 | 3.59E-3 |
| 1904668 | positive regulation of ubiquitin protein ligase activity | 7 | 5 | 1 | 4 | 3.59E-3 |
| 0015908 | fatty acid transport | 7 | 5 | 1 | 4 | 3.59E-3 |
| 0006107 | oxaloacetate metabolic process | 7 | 5 | 1 | 4 | 3.59E-3 |
| 0046031 | ADP metabolic process | 119 | 35 | 22 | 13 | 3.62E-3 |
| 0006096 | glycolytic process | 119 | 35 | 22 | 13 | 3.62E-3 |
| 0006757 | ATP generation from ADP | 119 | 35 | 22 | 13 | 3.62E-3 |
| 0042440 | pigment metabolic process | 136 | 39 | 26 | 13 | 3.62E-3 |
| 0009132 | nucleoside diphosphate metabolic process | 136 | 39 | 26 | 13 | 3.62E-3 |
| 1903046 | meiotic cell cycle process | 162 | 45 | 31 | 14 | 3.68E-3 |
| 0046939 | nucleotide phosphorylation | 132 | 38 | 25 | 13 | 3.76E-3 |
| 0071496 | cellular response to external stimulus | 74 | 24 | 14 | 10 | 3.81E-3 |
| 0031668 | cellular response to extracellular stimulus | 74 | 24 | 14 | 10 | 3.81E-3 |
| 0033043 | regulation of organelle organization | 211 | 56 | 40 | 16 | 3.92E-3 |
| 0051297 | centrosome organization | 22 | 10 | 4 | 6 | 4.00E-3 |
| 0010143 | cutin biosynthetic process | 22 | 10 | 4 | 6 | 4.00E-3 |
| 0051225 | spindle assembly | 47 | 17 | 9 | 8 | 4.04E-3 |
| 0009058 | biosynthetic process | 3478 | 715 | 657 | 58 | 4.07E-3 |
| 0005991 | trehalose metabolic process | 36 | 14 | 7 | 7 | 4.14E-3 |
| 0046395 | carboxylic acid catabolic process | 185 | 50 | 35 | 15 | 4.20E-3 |
| 0016054 | organic acid catabolic process | 185 | 50 | 35 | 15 | 4.20E-3 |
| 0009735 | response to cytokinin | 55 | 19 | 10 | 9 | 4.41E-3 |
| 0006995 | cellular response to nitrogen starvation | 19 | 9 | 4 | 5 | 4.48E-3 |
| 0007154 | cell communication | 83 | 26 | 16 | 10 | 4.53E-3 |
| 0070588 | calcium ion transmembrane transport | 40 | 15 | 8 | 7 | 4.56E-3 |
| 0006952 | defense response | 495 | 117 | 94 | 23 | 4.69E-3 |
| 0009199 | ribonucleoside triphosphate metabolic process | 186 | 50 | 35 | 15 | 4.71E-3 |
| 0035821 | modification of morphology or physiology of other organism | 16 | 8 | 3 | 5 | 4.87E-3 |
| 0006152 | purine nucleoside catabolic process | 16 | 8 | 3 | 5 | 4.87E-3 |
| 0008608 | attachment of spindle microtubules to kinetochore | 16 | 8 | 3 | 5 | 4.87E-3 |
| 0009833 | plant-type primary cell wall biogenesis | 44 | 16 | 8 | 8 | 4.91E-3 |
| 0071483 | cellular response to blue light | 13 | 7 | 2 | 5 | 5.04E-3 |
| 0035337 | fatty-acyl-CoA metabolic process | 13 | 7 | 2 | 5 | 5.04E-3 |
| 0009773 | photosynthetic electron transport in photosystem I | 13 | 7 | 2 | 5 | 5.04E-3 |
| 0009813 | flavonoid biosynthetic process | 26 | 11 | 5 | 6 | 5.05E-3 |
| 0009205 | purine ribonucleoside triphosphate metabolic process | 169 | 46 | 32 | 14 | 5.08E-3 |
| 0046034 | ATP metabolic process | 156 | 43 | 29 | 14 | 5.17E-3 |
| 0009123 | nucleoside monophosphate metabolic process | 268 | 68 | 51 | 17 | 5.22E-3 |
| 0009167 | purine ribonucleoside monophosphate metabolic process | 196 | 52 | 37 | 15 | 5.33E-3 |
| 0009126 | purine nucleoside monophosphate metabolic process | 196 | 52 | 37 | 15 | 5.33E-3 |
| 0070592 | cell wall polysaccharide biosynthetic process | 52 | 18 | 10 | 8 | 5.37E-3 |
| 1901657 | glycosyl compound metabolic process | 342 | 84 | 65 | 19 | 5.37E-3 |
| 0009185 | ribonucleoside diphosphate metabolic process | 126 | 36 | 24 | 12 | 5.38E-3 |
| 0010032 | meiotic chromosome condensation | 5 | 4 | 1 | 3 | 5.41E-3 |
| 0009311 | oligosaccharide metabolic process | 139 | 39 | 26 | 13 | 5.43E-3 |
| 0006006 | glucose metabolic process | 80 | 25 | 15 | 10 | 5.49E-3 |
| 0032482 | Rab protein signal transduction | 56 | 19 | 11 | 8 | 5.51E-3 |
| 0014070 | response to organic cyclic compound | 76 | 24 | 14 | 10 | 5.57E-3 |
| 0051726 | regulation of cell cycle | 306 | 76 | 58 | 18 | 5.85E-3 |
| 0019400 | alditol metabolic process | 23 | 10 | 4 | 6 | 5.90E-3 |
| 0006071 | glycerol metabolic process | 23 | 10 | 4 | 6 | 5.90E-3 |
| 0009141 | nucleoside triphosphate metabolic process | 197 | 52 | 37 | 15 | 5.94E-3 |
| 0000278 | mitotic cell cycle | 30 | 12 | 6 | 6 | 5.94E-3 |
| 0006790 | sulfur compound metabolic process | 367 | 89 | 69 | 20 | 6.15E-3 |
| 0044724 | single-organism carbohydrate catabolic process | 220 | 57 | 42 | 15 | 6.28E-3 |
| 0009144 | purine nucleoside triphosphate metabolic process | 171 | 46 | 32 | 14 | 6.41E-3 |
| 1901576 | organic substance biosynthetic process | 3220 | 661 | 609 | 52 | 6.50E-3 |
| 0009611 | response to wounding | 77 | 24 | 15 | 9 | 6.67E-3 |
| 0071478 | cellular response to radiation | 53 | 18 | 10 | 8 | 6.72E-3 |
| 0043266 | regulation of potassium ion transport | 3 | 3 | 1 | 2 | 6.75E-3 |
| 0006949 | syncytium formation | 3 | 3 | 1 | 2 | 6.75E-3 |
| 0048200 | Golgi transport vesicle coating | 3 | 3 | 1 | 2 | 6.75E-3 |
| 0048205 | COPI coating of Golgi vesicle | 3 | 3 | 1 | 2 | 6.75E-3 |
| 0006021 | inositol biosynthetic process | 3 | 3 | 1 | 2 | 6.75E-3 |
| 0009970 | cellular response to sulfate starvation | 3 | 3 | 1 | 2 | 6.75E-3 |
| 0051208 | sequestering of calcium ion | 3 | 3 | 1 | 2 | 6.75E-3 |
| 0009161 | ribonucleoside monophosphate metabolic process | 230 | 59 | 43 | 16 | 6.90E-3 |
| 0006355 | regulation of transcription, DNA-templated | 2110 | 442 | 399 | 43 | 7.02E-3 |
| 0016042 | lipid catabolic process | 253 | 64 | 48 | 16 | 7.05E-3 |
| 0031407 | oxylipin metabolic process | 27 | 11 | 5 | 6 | 7.09E-3 |
| 0031408 | oxylipin biosynthetic process | 27 | 11 | 5 | 6 | 7.09E-3 |
| 0009812 | flavonoid metabolic process | 27 | 11 | 5 | 6 | 7.09E-3 |
| 0009119 | ribonucleoside metabolic process | 281 | 70 | 53 | 17 | 7.34E-3 |
| 0046686 | response to cadmium ion | 90 | 27 | 17 | 10 | 7.36E-3 |
| 0030148 | sphingolipid biosynthetic process | 42 | 15 | 8 | 7 | 7.68E-3 |
| 0009751 | response to salicylic acid | 42 | 15 | 8 | 7 | 7.68E-3 |
| 0010200 | response to chitin | 17 | 8 | 3 | 5 | 7.69E-3 |
| 0009743 | response to carbohydrate | 46 | 16 | 9 | 7 | 8.00E-3 |
| 0009653 | anatomical structure morphogenesis | 173 | 46 | 33 | 13 | 8.03E-3 |
| 0090481 | pyrimidine nucleotide-sugar transmembrane transport | 31 | 12 | 6 | 6 | 8.06E-3 |
| 0071900 | regulation of protein serine/threonine kinase activity | 99 | 29 | 19 | 10 | 8.07E-3 |
| 0007076 | mitotic chromosome condensation | 8 | 5 | 2 | 3 | 8.09E-3 |
| 0009608 | response to symbiont | 8 | 5 | 2 | 3 | 8.09E-3 |
| 0009610 | response to symbiotic fungus | 8 | 5 | 2 | 3 | 8.09E-3 |
| 0000255 | allantoin metabolic process | 8 | 5 | 2 | 3 | 8.09E-3 |
| 0044380 | protein localization to cytoskeleton | 8 | 5 | 2 | 3 | 8.09E-3 |
| 0072698 | protein localization to microtubule cytoskeleton | 8 | 5 | 2 | 3 | 8.09E-3 |
| 0046165 | alcohol biosynthetic process | 70 | 22 | 13 | 9 | 8.23E-3 |
| 0046128 | purine ribonucleoside metabolic process | 241 | 61 | 46 | 15 | 8.24E-3 |
| 0008202 | steroid metabolic process | 160 | 43 | 30 | 13 | 8.32E-3 |
| 0009112 | nucleobase metabolic process | 66 | 21 | 12 | 9 | 8.32E-3 |
| 0051817 | modification of morphology or physiology of other organism involved in symbiotic interaction | 14 | 7 | 3 | 4 | 8.44E-3 |
| 0044003 | modification by symbiont of host morphology or physiology | 14 | 7 | 3 | 4 | 8.44E-3 |
| 0051315 | attachment of mitotic spindle microtubules to kinetochore | 11 | 6 | 2 | 4 | 8.76E-3 |
| 0009299 | mRNA transcription | 11 | 6 | 2 | 4 | 8.76E-3 |
| 0035336 | long-chain fatty-acyl-CoA metabolic process | 11 | 6 | 2 | 4 | 8.76E-3 |
| 0045859 | regulation of protein kinase activity | 165 | 44 | 31 | 13 | 8.87E-3 |
| 0043549 | regulation of kinase activity | 174 | 46 | 33 | 13 | 8.95E-3 |
| 0009617 | response to bacterium | 224 | 57 | 42 | 15 | 9.27E-3 |
| 0072511 | divalent inorganic cation transport | 100 | 29 | 19 | 10 | 9.35E-3 |
| 0070838 | divalent metal ion transport | 100 | 29 | 19 | 10 | 9.35E-3 |
| 0055086 | nucleobase-containing small molecule metabolic process | 511 | 118 | 97 | 21 | 9.64E-3 |
| 0061615 | glycolytic process through fructose-6-phosphate | 28 | 11 | 5 | 6 | 9.72E-3 |
| 0000280 | nuclear division | 28 | 11 | 5 | 6 | 9.72E-3 |
| 0007126 | meiotic nuclear division | 28 | 11 | 5 | 6 | 9.72E-3 |
| 0006334 | nucleosome assembly | 43 | 15 | 8 | 7 | 9.77E-3 |
| 0045491 | xylan metabolic process | 71 | 22 | 13 | 9 | 9.84E-3 |
| 0007098 | centrosome cycle | 21 | 9 | 4 | 5 | 9.93E-3 |
| 0043562 | cellular response to nitrogen levels | 21 | 9 | 4 | 5 | 9.93E-3 |
| 0009749 | response to glucose | 21 | 9 | 4 | 5 | 9.93E-3 |
| 0010817 | regulation of hormone levels | 184 | 48 | 35 | 13 | 9.99E-3 |
| 0042446 | hormone biosynthetic process | 92 | 27 | 17 | 10 | 0.01 |
| 0009699 | phenylpropanoid biosynthetic process | 55 | 18 | 10 | 8 | 0.01 |
| 0006066 | alcohol metabolic process | 118 | 33 | 22 | 11 | 0.01 |
| 0010109 | regulation of photosynthesis | 36 | 13 | 7 | 6 | 0.01 |
| 0071702 | organic substance transport | 1692 | 356 | 320 | 36 | 0.01 |
| 0006656 | phosphatidylcholine biosynthetic process | 18 | 8 | 3 | 5 | 0.01 |
| 0051438 | regulation of ubiquitin-protein transferase activity | 18 | 8 | 3 | 5 | 0.01 |
| 0032886 | regulation of microtubule-based process | 25 | 10 | 5 | 5 | 0.01 |
| 0006816 | calcium ion transport | 48 | 16 | 9 | 7 | 0.01 |
| 0003002 | regionalization | 52 | 17 | 10 | 7 | 0.01 |
| 0006665 | sphingolipid metabolic process | 81 | 24 | 15 | 9 | 0.01 |
| 0009767 | photosynthetic electron transport chain | 29 | 11 | 5 | 6 | 0.01 |
| 0010345 | suberin biosynthetic process | 15 | 7 | 3 | 4 | 0.01 |
| 0019563 | glycerol catabolic process | 15 | 7 | 3 | 4 | 0.01 |
| 0019405 | alditol catabolic process | 15 | 7 | 3 | 4 | 0.01 |
| 0008295 | spermidine biosynthetic process | 6 | 4 | 1 | 3 | 0.01 |
| 0042873 | aldonate transport | 6 | 4 | 1 | 3 | 0.01 |
| 0007187 | G-protein coupled receptor signaling pathway, coupled to cyclic nucleotide second messenger | 6 | 4 | 1 | 3 | 0.01 |
| 0007188 | adenylate cyclase-modulating G-protein coupled receptor signaling pathway | 6 | 4 | 1 | 3 | 0.01 |
| 0098542 | defense response to other organism | 331 | 79 | 63 | 16 | 0.01 |
| 0006144 | purine nucleobase metabolic process | 33 | 12 | 6 | 6 | 0.01 |
| 0034284 | response to monosaccharide | 22 | 9 | 4 | 5 | 0.01 |
| 0007140 | male meiosis | 22 | 9 | 4 | 5 | 0.01 |
| 0009746 | response to hexose | 22 | 9 | 4 | 5 | 0.01 |
| 0043101 | purine-containing compound salvage | 12 | 6 | 2 | 4 | 0.01 |
| 0009808 | lignin metabolic process | 61 | 19 | 12 | 7 | 0.01 |
| 0072531 | pyrimidine-containing compound transmembrane transport | 45 | 15 | 9 | 6 | 0.02 |
| 0009718 | anthocyanin-containing compound biosynthetic process | 9 | 5 | 2 | 3 | 0.02 |
| 0008356 | asymmetric cell division | 9 | 5 | 2 | 3 | 0.02 |
| 0042454 | ribonucleoside catabolic process | 9 | 5 | 2 | 3 | 0.02 |
| 0000727 | double-strand break repair via break-induced replication | 9 | 5 | 2 | 3 | 0.02 |
| 0010037 | response to carbon dioxide | 9 | 5 | 2 | 3 | 0.02 |
| 0051260 | protein homooligomerization | 26 | 10 | 5 | 5 | 0.02 |
| 1903506 | regulation of nucleic acid-templated transcription | 2153 | 445 | 407 | 38 | 0.02 |
| 2001141 | regulation of RNA biosynthetic process | 2153 | 445 | 407 | 38 | 0.02 |
| 0099402 | plant organ development | 117 | 32 | 22 | 10 | 0.02 |
| 0072524 | pyridine-containing compound metabolic process | 189 | 48 | 36 | 12 | 0.02 |
| 0009266 | response to temperature stimulus | 329 | 78 | 62 | 16 | 0.02 |
| 0050790 | regulation of catalytic activity | 618 | 138 | 117 | 21 | 0.02 |
| 0045893 | positive regulation of transcription, DNA-templated | 420 | 97 | 79 | 18 | 0.02 |
| 0009267 | cellular response to starvation | 62 | 19 | 12 | 7 | 0.02 |
| 0009063 | cellular amino acid catabolic process | 96 | 27 | 18 | 9 | 0.02 |
| 0043620 | regulation of DNA-templated transcription in response to stress | 34 | 12 | 6 | 6 | 0.02 |
| 0019932 | second-messenger-mediated signaling | 34 | 12 | 6 | 6 | 0.02 |
| 0006012 | galactose metabolic process | 34 | 12 | 6 | 6 | 0.02 |
| 0070589 | cellular component macromolecule biosynthetic process | 58 | 18 | 11 | 7 | 0.02 |
| 0019751 | polyol metabolic process | 58 | 18 | 11 | 7 | 0.02 |
| 0044038 | cell wall macromolecule biosynthetic process | 58 | 18 | 11 | 7 | 0.02 |
| 0009991 | response to extracellular stimulus | 105 | 29 | 20 | 9 | 0.02 |
| 1901701 | cellular response to oxygen-containing compound | 88 | 25 | 17 | 8 | 0.02 |
| 0071265 | L-methionine biosynthetic process | 16 | 7 | 3 | 4 | 0.02 |
| 0019362 | pyridine nucleotide metabolic process | 182 | 46 | 34 | 12 | 0.02 |
| 0070887 | cellular response to chemical stimulus | 173 | 44 | 33 | 11 | 0.02 |
| 0071249 | cellular response to nitrate | 31 | 11 | 6 | 5 | 0.02 |
| 0036003 | positive regulation of transcription from RNA polymerase II promoter in response to stress | 31 | 11 | 6 | 5 | 0.02 |
| 0006835 | dicarboxylic acid transport | 31 | 11 | 6 | 5 | 0.02 |
| 0061408 | positive regulation of transcription from RNA polymerase II promoter in response to heat stress | 31 | 11 | 6 | 5 | 0.02 |
| 0050832 | defense response to fungus | 111 | 30 | 21 | 9 | 0.02 |
| 0071840 | cellular component organization or biogenesis | 2980 | 604 | 563 | 41 | 0.02 |
| 0072488 | ammonium transmembrane transport | 13 | 6 | 2 | 4 | 0.02 |
| 0015813 | L-glutamate transport | 13 | 6 | 2 | 4 | 0.02 |
| 0015696 | ammonium transport | 13 | 6 | 2 | 4 | 0.02 |
| 0009623 | response to parasitic fungus | 4 | 3 | 1 | 2 | 0.02 |
| 0051339 | regulation of lyase activity | 4 | 3 | 1 | 2 | 0.02 |
| 0045761 | regulation of adenylate cyclase activity | 4 | 3 | 1 | 2 | 0.02 |
| 0030817 | regulation of cAMP biosynthetic process | 4 | 3 | 1 | 2 | 0.02 |
| 0030814 | regulation of cAMP metabolic process | 4 | 3 | 1 | 2 | 0.02 |
| 0030802 | regulation of cyclic nucleotide biosynthetic process | 4 | 3 | 1 | 2 | 0.02 |
| 0030799 | regulation of cyclic nucleotide metabolic process | 4 | 3 | 1 | 2 | 0.02 |
| 0019500 | cyanide catabolic process | 4 | 3 | 1 | 2 | 0.02 |
| 0010236 | plastoquinone biosynthetic process | 4 | 3 | 1 | 2 | 0.02 |
| 0032469 | endoplasmic reticulum calcium ion homeostasis | 4 | 3 | 1 | 2 | 0.02 |
| 0009956 | radial pattern formation | 4 | 3 | 1 | 2 | 0.02 |
| 0070507 | regulation of microtubule cytoskeleton organization | 20 | 8 | 4 | 4 | 0.02 |
| 0046173 | polyol biosynthetic process | 20 | 8 | 4 | 4 | 0.02 |
| 0046184 | aldehyde biosynthetic process | 20 | 8 | 4 | 4 | 0.02 |
| 0072350 | tricarboxylic acid metabolic process | 68 | 20 | 13 | 7 | 0.02 |
| 0007389 | pattern specification process | 81 | 23 | 15 | 8 | 0.02 |
| 0046496 | nicotinamide nucleotide metabolic process | 180 | 45 | 34 | 11 | 0.03 |
| 0043648 | dicarboxylic acid metabolic process | 121 | 32 | 23 | 9 | 0.03 |
| 0006869 | lipid transport | 171 | 43 | 32 | 11 | 0.03 |
| 0033356 | UDP-L-arabinose metabolic process | 10 | 5 | 2 | 3 | 0.03 |
| 0001709 | cell fate determination | 10 | 5 | 2 | 3 | 0.03 |
| 0016584 | nucleosome positioning | 10 | 5 | 2 | 3 | 0.03 |
| 0046283 | anthocyanin-containing compound metabolic process | 10 | 5 | 2 | 3 | 0.03 |
| 0032544 | plastid translation | 10 | 5 | 2 | 3 | 0.03 |
| 0006268 | DNA unwinding involved in DNA replication | 10 | 5 | 2 | 3 | 0.03 |
| 0019509 | L-methionine biosynthetic process from methylthioadenosine | 10 | 5 | 2 | 3 | 0.03 |
| 0010016 | shoot system morphogenesis | 10 | 5 | 2 | 3 | 0.03 |
| 0051252 | regulation of RNA metabolic process | 2233 | 457 | 422 | 35 | 0.03 |
| 0034440 | lipid oxidation | 56 | 17 | 11 | 6 | 0.03 |
| 0019219 | regulation of nucleobase-containing compound metabolic process | 2325 | 475 | 439 | 36 | 0.03 |
| 0031323 | regulation of cellular metabolic process | 2930 | 593 | 554 | 39 | 0.03 |
| 1901136 | carbohydrate derivative catabolic process | 73 | 21 | 14 | 7 | 0.03 |
| 0065009 | regulation of molecular function | 647 | 142 | 122 | 20 | 0.03 |
| 0042445 | hormone metabolic process | 126 | 33 | 24 | 9 | 0.03 |
| 0009652 | thigmotropism | 7 | 4 | 1 | 3 | 0.03 |
| 0090116 | C-5 methylation of cytosine | 7 | 4 | 1 | 3 | 0.03 |
| 0008216 | spermidine metabolic process | 7 | 4 | 1 | 3 | 0.03 |
| 0036294 | cellular response to decreased oxygen levels | 7 | 4 | 1 | 3 | 0.03 |
| 0046130 | purine ribonucleoside catabolic process | 7 | 4 | 1 | 3 | 0.03 |
| 0010424 | DNA methylation on cytosine within a CG sequence | 7 | 4 | 1 | 3 | 0.03 |
| 2000034 | regulation of seed maturation | 7 | 4 | 1 | 3 | 0.03 |
| 1904825 | protein localization to microtubule plus-end | 7 | 4 | 1 | 3 | 0.03 |
| 0015808 | L-alanine transport | 7 | 4 | 1 | 3 | 0.03 |
| 0015812 | gamma-aminobutyric acid transport | 7 | 4 | 1 | 3 | 0.03 |
| 0071453 | cellular response to oxygen levels | 7 | 4 | 1 | 3 | 0.03 |
| 0032328 | alanine transport | 7 | 4 | 1 | 3 | 0.03 |
| 0035372 | protein localization to microtubule | 7 | 4 | 1 | 3 | 0.03 |
| 0009685 | gibberellin metabolic process | 32 | 11 | 6 | 5 | 0.03 |
| 0010928 | regulation of auxin mediated signaling pathway | 32 | 11 | 6 | 5 | 0.03 |
| 0043618 | regulation of transcription from RNA polymerase II promoter in response to stress | 32 | 11 | 6 | 5 | 0.03 |
| 0009637 | response to blue light | 44 | 14 | 8 | 6 | 0.03 |
| 0008154 | actin polymerization or depolymerization | 17 | 7 | 3 | 4 | 0.03 |
| 0009867 | jasmonic acid mediated signaling pathway | 17 | 7 | 3 | 4 | 0.03 |
| 1902680 | positive regulation of RNA biosynthetic process | 438 | 99 | 83 | 16 | 0.03 |
| 1903508 | positive regulation of nucleic acid-templated transcription | 438 | 99 | 83 | 16 | 0.03 |
| 0000272 | polysaccharide catabolic process | 186 | 46 | 35 | 11 | 0.03 |
| 0031669 | cellular response to nutrient levels | 65 | 19 | 12 | 7 | 0.03 |
| 2000112 | regulation of cellular macromolecule biosynthetic process | 2334 | 476 | 441 | 35 | 0.03 |
| 0006101 | citrate metabolic process | 61 | 18 | 12 | 6 | 0.03 |
| 0006099 | tricarboxylic acid cycle | 61 | 18 | 12 | 6 | 0.03 |
| 0007052 | mitotic spindle organization | 21 | 8 | 4 | 4 | 0.03 |
| 0007143 | female meiotic division | 21 | 8 | 4 | 4 | 0.03 |
| 0019222 | regulation of metabolic process | 3149 | 634 | 595 | 39 | 0.03 |
| 1901698 | response to nitrogen compound | 114 | 30 | 22 | 8 | 0.03 |
| 0009742 | brassinosteroid mediated signaling pathway | 53 | 16 | 10 | 6 | 0.03 |
| 1901575 | organic substance catabolic process | 1813 | 373 | 343 | 30 | 0.03 |
| 0046394 | carboxylic acid biosynthetic process | 563 | 124 | 106 | 18 | 0.03 |
| 0016053 | organic acid biosynthetic process | 563 | 124 | 106 | 18 | 0.03 |
| 0007166 | cell surface receptor signaling pathway | 475 | 106 | 90 | 16 | 0.03 |
| 0009695 | jasmonic acid biosynthetic process | 14 | 6 | 3 | 3 | 0.03 |
| 0015800 | acidic amino acid transport | 14 | 6 | 3 | 3 | 0.03 |
| 0009150 | purine ribonucleotide metabolic process | 254 | 60 | 48 | 12 | 0.03 |
| 0000266 | mitochondrial fission | 29 | 10 | 5 | 5 | 0.03 |
| 0016049 | cell growth | 106 | 28 | 20 | 8 | 0.04 |
| 1900866 | glycolate transport | 2 | 2 | 0 | 2 | 0.04 |
| 0033396 | beta-alanine biosynthetic process via 3-ureidopropionate | 2 | 2 | 0 | 2 | 0.04 |
| 0048657 | anther wall tapetum cell differentiation | 2 | 2 | 0 | 2 | 0.04 |
| 0001560 | regulation of cell growth by extracellular stimulus | 2 | 2 | 0 | 2 | 0.04 |
| 0001505 | regulation of neurotransmitter levels | 2 | 2 | 0 | 2 | 0.04 |
| 0019628 | urate catabolic process | 2 | 2 | 0 | 2 | 0.04 |
| 0080153 | negative regulation of reductive pentose-phosphate cycle | 2 | 2 | 0 | 2 | 0.04 |
| 0080152 | regulation of reductive pentose-phosphate cycle | 2 | 2 | 0 | 2 | 0.04 |
| 0006272 | leading strand elongation | 2 | 2 | 0 | 2 | 0.04 |
| 0090410 | malonate catabolic process | 2 | 2 | 0 | 2 | 0.04 |
| 2000603 | regulation of secondary growth | 2 | 2 | 0 | 2 | 0.04 |
| 2000605 | positive regulation of secondary growth | 2 | 2 | 0 | 2 | 0.04 |
| 0019482 | beta-alanine metabolic process | 2 | 2 | 0 | 2 | 0.04 |
| 0019483 | beta-alanine biosynthetic process | 2 | 2 | 0 | 2 | 0.04 |
| 0006148 | inosine catabolic process | 2 | 2 | 0 | 2 | 0.04 |
| 0019428 | allantoin biosynthetic process | 2 | 2 | 0 | 2 | 0.04 |
| 0046415 | urate metabolic process | 2 | 2 | 0 | 2 | 0.04 |
| 0010110 | regulation of photosynthesis, dark reaction | 2 | 2 | 0 | 2 | 0.04 |
| 0010053 | root epidermal cell differentiation | 2 | 2 | 0 | 2 | 0.04 |
| 1901975 | glycerate transmembrane transport | 2 | 2 | 0 | 2 | 0.04 |
| 0050691 | regulation of defense response to virus by host | 2 | 2 | 0 | 2 | 0.04 |
| 0097339 | glycolate transmembrane transport | 2 | 2 | 0 | 2 | 0.04 |
| 0044248 | cellular catabolic process | 1432 | 297 | 271 | 26 | 0.04 |
| 0040008 | regulation of growth | 120 | 31 | 23 | 8 | 0.04 |
| 0006635 | fatty acid beta-oxidation | 54 | 16 | 10 | 6 | 0.04 |
| 0009826 | unidimensional cell growth | 80 | 22 | 15 | 7 | 0.04 |
| 1901564 | organonitrogen compound metabolic process | 2034 | 415 | 384 | 31 | 0.04 |
| 0042742 | defense response to bacterium | 213 | 51 | 40 | 11 | 0.04 |
| 1902475 | L-alpha-amino acid transmembrane transport | 18 | 7 | 3 | 4 | 0.04 |
| 0006163 | purine nucleotide metabolic process | 270 | 63 | 51 | 12 | 0.04 |
| 0098655 | cation transmembrane transport | 616 | 134 | 116 | 18 | 0.04 |
| 0015698 | inorganic anion transport | 148 | 37 | 28 | 9 | 0.04 |
| 0016043 | cellular component organization | 2877 | 579 | 544 | 35 | 0.04 |
| 0061640 | cytoskeleton-dependent cytokinesis | 11 | 5 | 2 | 3 | 0.04 |
| 0017157 | regulation of exocytosis | 11 | 5 | 2 | 3 | 0.04 |
| 0000910 | cytokinesis | 11 | 5 | 2 | 3 | 0.04 |
| 1903530 | regulation of secretion by cell | 11 | 5 | 2 | 3 | 0.04 |
| 0006140 | regulation of nucleotide metabolic process | 11 | 5 | 2 | 3 | 0.04 |
| 0010093 | specification of floral organ identity | 11 | 5 | 2 | 3 | 0.04 |
| 0090701 | specification of plant organ identity | 11 | 5 | 2 | 3 | 0.04 |
| 1900542 | regulation of purine nucleotide metabolic process | 11 | 5 | 2 | 3 | 0.04 |
| 0060255 | regulation of macromolecule metabolic process | 3012 | 605 | 569 | 36 | 0.04 |
| 1901606 | alpha-amino acid catabolic process | 85 | 23 | 16 | 7 | 0.04 |
| 0080090 | regulation of primary metabolic process | 2889 | 581 | 546 | 35 | 0.04 |
| 0031396 | regulation of protein ubiquitination | 22 | 8 | 4 | 4 | 0.04 |
| 0045815 | positive regulation of gene expression, epigenetic | 22 | 8 | 4 | 4 | 0.04 |
| 0009083 | branched-chain amino acid catabolic process | 22 | 8 | 4 | 4 | 0.04 |
| 0009259 | ribonucleotide metabolic process | 295 | 68 | 56 | 12 | 0.04 |
| 0048511 | rhythmic process | 38 | 12 | 7 | 5 | 0.04 |
| 0007623 | circadian rhythm | 38 | 12 | 7 | 5 | 0.04 |
| 0015850 | organic hydroxy compound transport | 38 | 12 | 7 | 5 | 0.04 |
| 0030198 | extracellular matrix organization | 26 | 9 | 5 | 4 | 0.04 |
| 0043062 | extracellular structure organization | 26 | 9 | 5 | 4 | 0.04 |
| 0016106 | sesquiterpenoid biosynthetic process | 26 | 9 | 5 | 4 | 0.04 |
| 0051275 | beta-glucan catabolic process | 34 | 11 | 6 | 5 | 0.04 |
| 0030245 | cellulose catabolic process | 34 | 11 | 6 | 5 | 0.04 |
| 0019722 | calcium-mediated signaling | 30 | 10 | 6 | 4 | 0.04 |
| 0015995 | chlorophyll biosynthetic process | 30 | 10 | 6 | 4 | 0.04 |
| 0019388 | galactose catabolic process | 30 | 10 | 6 | 4 | 0.04 |
| 0019395 | fatty acid oxidation | 55 | 16 | 10 | 6 | 0.04 |
| 0043401 | steroid hormone mediated signaling pathway | 55 | 16 | 10 | 6 | 0.04 |
| 0010557 | positive regulation of macromolecule biosynthetic process | 476 | 105 | 90 | 15 | 0.04 |
| 0009891 | positive regulation of biosynthetic process | 486 | 107 | 92 | 15 | 0.05 |
| 0048518 | positive regulation of biological process | 964 | 203 | 182 | 21 | 0.05 |
| 0006657 | CDP-choline pathway | 8 | 4 | 2 | 2 | 0.05 |
| 0002252 | immune effector process | 8 | 4 | 2 | 2 | 0.05 |
| 0000281 | mitotic cytokinesis | 8 | 4 | 2 | 2 | 0.05 |
| 0046166 | glyceraldehyde-3-phosphate biosynthetic process | 8 | 4 | 2 | 2 | 0.05 |
| 0043903 | regulation of symbiosis, encompassing mutualism through parasitism | 8 | 4 | 2 | 2 | 0.05 |
| 0071163 | DNA replication preinitiation complex assembly | 8 | 4 | 2 | 2 | 0.05 |
| 0031110 | regulation of microtubule polymerization or depolymerization | 8 | 4 | 2 | 2 | 0.05 |
| 1902074 | response to salt | 8 | 4 | 2 | 2 | 0.05 |
| 0031146 | SCF-dependent proteasomal ubiquitin-dependent protein catabolic process | 95 | 25 | 18 | 7 | 0.05 |
| 0043102 | amino acid salvage | 15 | 6 | 3 | 3 | 0.05 |
| 0071267 | L-methionine salvage | 15 | 6 | 3 | 3 | 0.05 |
| 1902022 | L-lysine transport | 15 | 6 | 3 | 3 | 0.05 |
| 0031952 | regulation of protein autophosphorylation | 15 | 6 | 3 | 3 | 0.05 |
| 0015819 | lysine transport | 15 | 6 | 3 | 3 | 0.05 |
| 1903401 | L-lysine transmembrane transport | 15 | 6 | 3 | 3 | 0.05 |
| 0015706 | nitrate transport | 15 | 6 | 3 | 3 | 0.05 |
| 0010468 | regulation of gene expression | 2531 | 510 | 478 | 32 | 0.05 |
| 0010628 | positive regulation of gene expression | 473 | 104 | 89 | 15 | 0.05 |
| 0009062 | fatty acid catabolic process | 60 | 17 | 11 | 6 | 0.05 |
| 0051444 | negative regulation of ubiquitin-protein transferase activity | 5 | 3 | 1 | 2 | 0.05 |
| 0055069 | zinc ion homeostasis | 5 | 3 | 1 | 2 | 0.05 |
| 0051553 | flavone biosynthetic process | 5 | 3 | 1 | 2 | 0.05 |
| 0051552 | flavone metabolic process | 5 | 3 | 1 | 2 | 0.05 |
| 1904667 | negative regulation of ubiquitin protein ligase activity | 5 | 3 | 1 | 2 | 0.05 |
| 0048830 | adventitious root development | 5 | 3 | 1 | 2 | 0.05 |
| 0080148 | negative regulation of response to water deprivation | 5 | 3 | 1 | 2 | 0.05 |
| 0031279 | regulation of cyclase activity | 5 | 3 | 1 | 2 | 0.05 |
| 0072529 | pyrimidine-containing compound catabolic process | 5 | 3 | 1 | 2 | 0.05 |
| 0019499 | cyanide metabolic process | 5 | 3 | 1 | 2 | 0.05 |
| 0071456 | cellular response to hypoxia | 5 | 3 | 1 | 2 | 0.05 |
| 0015790 | UDP-xylose transport | 5 | 3 | 1 | 2 | 0.05 |
| 0032264 | IMP salvage | 5 | 3 | 1 | 2 | 0.05 |
| 0009081 | branched-chain amino acid metabolic process | 43 | 13 | 8 | 5 | 0.05 |
