# Supplementary material for: Reproductive developmental transcriptome analysis of Tripidium ravennae (Poaceae)
Source: BMC Genomics. 2021 Jun 28;22:483. doi: 10.1186/s12864-021-07641-y (PMC8237498; doi:10.1186/s12864-021-07641-y)
Supplement: Supplementary file 1 — Additional file 1: Table S1. Sequencing statistics. Figure S1a-c. Transcriptome assembly. Figure S2. Annotation statistics for primary de novo assembly. Figure S3. Annotation statistics for cluster enriched assembly. Figure S4. Annotation statistics for PB Iso-Seq sequences. Table S2. GO-term enrichment for upregulated transcripts during inflorescence development. Table S3. GO-term enrichment for upregulated transcripts during flower development. Table S4. GO-term enrichment for upregulated transcripts during seed development. Table S5. Excel workbook including summaries of DEG’s in inflorescence development. Table S6. Excel workbook including summaries of DEG’s in floral development. Table S7. Excel workbook including summaries of DEG’s in seed development. Supplemental List 1. List of FASTA formatted sequences associated with Fig. 8 and Tables 2, 3, and 4. Table S8. Table export of annotations for the cluster enriched de novo transcriptome assembly. Table S9. Table export of annotations for the collapsed Iso-seq transcript set. [file 12864_2021_7641_MOESM1_ESM.zip › ST3-GOEnrichAnalysisDuringFloralDvlpmt.docx]

**Reproductive developmental transcriptome analysis of *Tripidium ravennae* (Poaceae)**

Nathan Maren^1^*, Fangzhou Zhao^1,2^, Rishi Aryal^1^, Darren Touchell^3^, Wusheng Liu^1^, Thomas Ranney^3^, and Hamid Ashrafi^1*^

^1^Department of Horticultural Science, North Carolina State University, Campus Box 7609, Raleigh, NC 27695-7609, USA

^2^College of Agriculture, Nanjing Agricultural University, Nanjing 210095, China

^3^Mountain Crop Improvement Lab, Department of Horticultural Science, Mountain Horticultural Crops Research and Extension Center, North Carolina State University, 455 Research Drive, Mills River, NC 28759-3423, USA

*Corresponding authors: hamidashrafi@ncsu.edu and namaren@ncsu.edu

| **Category** | **Description** | **Full set** | **In subset** | **Expected in subset** | **Observed - expected** | **p-value** |
| --- | --- | --- | --- | --- | --- | --- |
| 0044710 | single-organism metabolic process | 5645 | 1420 | 1121 | 299 | 3.33E-16 |
| 0055085 | transmembrane transport | 2005 | 551 | 398 | 153 | 7.77E-16 |
| 0055114 | oxidation-reduction process | 2524 | 683 | 501 | 182 | 7.77E-16 |
| 0044699 | single-organism process | 9115 | 2107 | 1811 | 296 | 8.88E-16 |
| 0006796 | phosphate-containing compound metabolic process | 4294 | 1034 | 853 | 181 | 5.01E-14 |
| 0006793 | phosphorus metabolic process | 4353 | 1044 | 865 | 179 | 1.12E-13 |
| 0006811 | ion transport | 1269 | 353 | 252 | 101 | 1.61E-12 |
| 0006952 | defense response | 495 | 164 | 98 | 66 | 1.66E-12 |
| 0009605 | response to external stimulus | 565 | 182 | 112 | 70 | 1.67E-12 |
| 0042221 | response to chemical | 896 | 263 | 178 | 85 | 3.09E-12 |
| 0051707 | response to other organism | 381 | 133 | 76 | 57 | 3.50E-12 |
| 0043207 | response to external biotic stimulus | 391 | 135 | 78 | 57 | 5.92E-12 |
| 0009607 | response to biotic stimulus | 415 | 141 | 82 | 59 | 7.75E-12 |
| 0006468 | protein phosphorylation | 2390 | 602 | 475 | 127 | 1.43E-11 |
| 0005984 | disaccharide metabolic process | 100 | 50 | 20 | 30 | 1.48E-11 |
| 0008299 | isoprenoid biosynthetic process | 141 | 63 | 28 | 35 | 1.96E-11 |
| 0009617 | response to bacterium | 224 | 87 | 44 | 43 | 3.91E-11 |
| 0034220 | ion transmembrane transport | 871 | 252 | 173 | 79 | 4.46E-11 |
| 0016311 | dephosphorylation | 518 | 165 | 103 | 62 | 4.88E-11 |
| 0044712 | single-organism catabolic process | 706 | 209 | 140 | 69 | 2.42E-10 |
| 0044711 | single-organism biosynthetic process | 1908 | 486 | 379 | 107 | 3.22E-10 |
| 0009611 | response to wounding | 77 | 40 | 15 | 25 | 3.57E-10 |
| 0016114 | terpenoid biosynthetic process | 98 | 47 | 19 | 28 | 3.68E-10 |
| 0006720 | isoprenoid metabolic process | 187 | 74 | 37 | 37 | 4.03E-10 |
| 0003333 | amino acid transmembrane transport | 122 | 54 | 24 | 30 | 7.98E-10 |
| 0042440 | pigment metabolic process | 136 | 58 | 27 | 31 | 1.10E-9 |
| 0006865 | amino acid transport | 130 | 56 | 26 | 30 | 1.35E-9 |
| 1903825 | organic acid transmembrane transport | 134 | 57 | 27 | 30 | 1.72E-9 |
| 1905039 | carboxylic acid transmembrane transport | 134 | 57 | 27 | 30 | 1.72E-9 |
| 0051704 | multi-organism process | 473 | 148 | 94 | 54 | 1.88E-9 |
| 0006749 | glutathione metabolic process | 131 | 56 | 26 | 30 | 1.91E-9 |
| 0001101 | response to acid chemical | 365 | 120 | 73 | 47 | 2.61E-9 |
| 0098542 | defense response to other organism | 331 | 111 | 66 | 45 | 2.93E-9 |
| 0006812 | cation transport | 752 | 215 | 149 | 66 | 3.63E-9 |
| 0009628 | response to abiotic stimulus | 885 | 246 | 176 | 70 | 4.62E-9 |
| 0046351 | disaccharide biosynthetic process | 51 | 29 | 10 | 19 | 6.15E-9 |
| 0006790 | sulfur compound metabolic process | 367 | 119 | 73 | 46 | 7.51E-9 |
| 0044283 | small molecule biosynthetic process | 754 | 214 | 150 | 64 | 7.62E-9 |
| 0009311 | oligosaccharide metabolic process | 139 | 57 | 28 | 29 | 8.71E-9 |
| 0042742 | defense response to bacterium | 213 | 78 | 42 | 36 | 8.96E-9 |
| 0015802 | basic amino acid transport | 16 | 14 | 3 | 11 | 1.17E-8 |
| 0007165 | signal transduction | 1357 | 352 | 270 | 82 | 1.37E-8 |
| 0006721 | terpenoid metabolic process | 134 | 55 | 27 | 28 | 1.52E-8 |
| 0044281 | small molecule metabolic process | 2086 | 513 | 414 | 99 | 2.00E-8 |
| 0044255 | cellular lipid metabolic process | 981 | 265 | 195 | 70 | 2.11E-8 |
| 0006082 | organic acid metabolic process | 1340 | 347 | 266 | 81 | 2.12E-8 |
| 0009620 | response to fungus | 132 | 54 | 26 | 28 | 2.34E-8 |
| 0009719 | response to endogenous stimulus | 422 | 131 | 84 | 47 | 2.66E-8 |
| 1901700 | response to oxygen-containing compound | 519 | 155 | 103 | 52 | 2.66E-8 |
| 0009416 | response to light stimulus | 282 | 95 | 56 | 39 | 3.04E-8 |
| 0015994 | chlorophyll metabolic process | 54 | 29 | 11 | 18 | 3.53E-8 |
| 0006629 | lipid metabolic process | 1355 | 349 | 269 | 80 | 3.53E-8 |
| 1901565 | organonitrogen compound catabolic process | 209 | 75 | 42 | 33 | 4.58E-8 |
| 0046148 | pigment biosynthetic process | 114 | 48 | 23 | 25 | 4.73E-8 |
| 0043436 | oxoacid metabolic process | 1337 | 344 | 266 | 78 | 5.00E-8 |
| 1902022 | L-lysine transport | 15 | 13 | 3 | 10 | 5.18E-8 |
| 0015819 | lysine transport | 15 | 13 | 3 | 10 | 5.18E-8 |
| 1903401 | L-lysine transmembrane transport | 15 | 13 | 3 | 10 | 5.18E-8 |
| 0005985 | sucrose metabolic process | 58 | 30 | 12 | 18 | 6.35E-8 |
| 0009725 | response to hormone | 392 | 122 | 78 | 44 | 6.72E-8 |
| 0010033 | response to organic substance | 526 | 155 | 104 | 51 | 6.90E-8 |
| 0019752 | carboxylic acid metabolic process | 1297 | 334 | 258 | 76 | 7.18E-8 |
| 0009312 | oligosaccharide biosynthetic process | 77 | 36 | 15 | 21 | 9.30E-8 |
| 0050896 | response to stimulus | 2995 | 704 | 595 | 109 | 9.73E-8 |
| 0098655 | cation transmembrane transport | 616 | 176 | 122 | 54 | 9.86E-8 |
| 0016310 | phosphorylation | 3316 | 772 | 659 | 113 | 1.09E-7 |
| 0098656 | anion transmembrane transport | 240 | 82 | 48 | 34 | 1.31E-7 |
| 0006820 | anion transport | 463 | 138 | 92 | 46 | 1.72E-7 |
| 1902475 | L-alpha-amino acid transmembrane transport | 18 | 14 | 4 | 10 | 1.99E-7 |
| 0035556 | intracellular signal transduction | 460 | 137 | 91 | 46 | 2.00E-7 |
| 0009414 | response to water deprivation | 137 | 53 | 27 | 26 | 2.75E-7 |
| 0006778 | porphyrin-containing compound metabolic process | 80 | 36 | 16 | 20 | 3.06E-7 |
| 0071705 | nitrogen compound transport | 530 | 153 | 105 | 48 | 3.37E-7 |
| 0015979 | photosynthesis | 97 | 41 | 19 | 22 | 3.87E-7 |
| 0009314 | response to radiation | 300 | 96 | 60 | 36 | 4.01E-7 |
| 0015807 | L-amino acid transport | 21 | 15 | 4 | 11 | 4.62E-7 |
| 0009415 | response to water | 139 | 53 | 28 | 25 | 4.71E-7 |
| 0005992 | trehalose biosynthetic process | 31 | 19 | 6 | 13 | 5.24E-7 |
| 0033013 | tetrapyrrole metabolic process | 82 | 36 | 16 | 20 | 6.43E-7 |
| 0046942 | carboxylic acid transport | 184 | 65 | 37 | 28 | 6.66E-7 |
| 0015849 | organic acid transport | 184 | 65 | 37 | 28 | 6.66E-7 |
| 0002376 | immune system process | 123 | 48 | 24 | 24 | 7.30E-7 |
| 0006575 | cellular modified amino acid metabolic process | 204 | 70 | 41 | 29 | 8.77E-7 |
| 0006464 | cellular protein modification process | 4360 | 982 | 866 | 116 | 9.62E-7 |
| 0036211 | protein modification process | 4360 | 982 | 866 | 116 | 9.62E-7 |
| 0015813 | L-glutamate transport | 13 | 11 | 3 | 8 | 9.84E-7 |
| 0009816 | defense response to bacterium, incompatible interaction | 22 | 15 | 4 | 11 | 1.18E-6 |
| 0006955 | immune response | 105 | 42 | 21 | 21 | 1.65E-6 |
| 0005975 | carbohydrate metabolic process | 1389 | 345 | 276 | 69 | 1.86E-6 |
| 1901605 | alpha-amino acid metabolic process | 374 | 112 | 74 | 38 | 1.86E-6 |
| 0051234 | establishment of localization | 3597 | 818 | 715 | 103 | 2.26E-6 |
| 0045087 | innate immune response | 103 | 41 | 20 | 21 | 2.52E-6 |
| 0006810 | transport | 3568 | 811 | 709 | 102 | 2.73E-6 |
| 0046434 | organophosphate catabolic process | 42 | 22 | 8 | 14 | 2.74E-6 |
| 0015800 | acidic amino acid transport | 14 | 11 | 3 | 8 | 3.76E-6 |
| 0008643 | carbohydrate transport | 204 | 68 | 41 | 27 | 3.95E-6 |
| 0009738 | abscisic acid-activated signaling pathway | 126 | 47 | 25 | 22 | 4.15E-6 |
| 0009686 | gibberellin biosynthetic process | 12 | 10 | 2 | 8 | 4.22E-6 |
| 0010035 | response to inorganic substance | 376 | 111 | 75 | 36 | 4.31E-6 |
| 0051179 | localization | 3732 | 843 | 741 | 102 | 4.48E-6 |
| 0030001 | metal ion transport | 360 | 107 | 72 | 35 | 4.55E-6 |
| 0008610 | lipid biosynthetic process | 703 | 188 | 140 | 48 | 4.68E-6 |
| 0044765 | single-organism transport | 1561 | 379 | 310 | 69 | 5.89E-6 |
| 0005987 | sucrose catabolic process | 24 | 15 | 5 | 10 | 6.03E-6 |
| 1902578 | single-organism localization | 1632 | 394 | 324 | 70 | 6.77E-6 |
| 0015711 | organic anion transport | 314 | 95 | 62 | 33 | 6.81E-6 |
| 0009056 | catabolic process | 1969 | 467 | 391 | 76 | 6.84E-6 |
| 0051262 | protein tetramerization | 17 | 12 | 3 | 9 | 8.44E-6 |
| 0070413 | trehalose metabolism in response to stress | 17 | 12 | 3 | 9 | 8.44E-6 |
| 0050832 | defense response to fungus | 111 | 42 | 22 | 20 | 8.74E-6 |
| 1901419 | regulation of response to alcohol | 33 | 18 | 7 | 11 | 1.06E-5 |
| 0009787 | regulation of abscisic acid-activated signaling pathway | 33 | 18 | 7 | 11 | 1.06E-5 |
| 0046777 | protein autophosphorylation | 194 | 64 | 39 | 25 | 1.09E-5 |
| 0005991 | trehalose metabolic process | 36 | 19 | 7 | 12 | 1.14E-5 |
| 0048585 | negative regulation of response to stimulus | 105 | 40 | 21 | 19 | 1.17E-5 |
| 0009636 | response to toxic substance | 39 | 20 | 8 | 12 | 1.18E-5 |
| 0044763 | single-organism cellular process | 5600 | 1225 | 1112 | 113 | 1.20E-5 |
| 0098660 | inorganic ion transmembrane transport | 622 | 167 | 124 | 43 | 1.23E-5 |
| 0048878 | chemical homeostasis | 306 | 92 | 61 | 31 | 1.24E-5 |
| 0051188 | cofactor biosynthetic process | 294 | 89 | 58 | 31 | 1.27E-5 |
| 1901615 | organic hydroxy compound metabolic process | 315 | 94 | 63 | 31 | 1.40E-5 |
| 1901606 | alpha-amino acid catabolic process | 85 | 34 | 17 | 17 | 1.56E-5 |
| 0042537 | benzene-containing compound metabolic process | 46 | 22 | 9 | 13 | 1.84E-5 |
| 0009753 | response to jasmonic acid | 37 | 19 | 7 | 12 | 1.90E-5 |
| 0016102 | diterpenoid biosynthetic process | 23 | 14 | 5 | 9 | 1.92E-5 |
| 1901575 | organic substance catabolic process | 1813 | 429 | 360 | 69 | 2.09E-5 |
| 0014070 | response to organic cyclic compound | 76 | 31 | 15 | 16 | 2.30E-5 |
| 0006470 | protein dephosphorylation | 262 | 80 | 52 | 28 | 2.43E-5 |
| 0009308 | amine metabolic process | 108 | 40 | 21 | 19 | 2.53E-5 |
| 0009635 | response to herbicide | 29 | 16 | 6 | 10 | 2.72E-5 |
| 0034219 | carbohydrate transmembrane transport | 131 | 46 | 26 | 20 | 3.18E-5 |
| 0046394 | carboxylic acid biosynthetic process | 563 | 151 | 112 | 39 | 3.32E-5 |
| 0016053 | organic acid biosynthetic process | 563 | 151 | 112 | 39 | 3.32E-5 |
| 0065007 | biological regulation | 5969 | 1294 | 1186 | 108 | 3.50E-5 |
| 2000022 | regulation of jasmonic acid mediated signaling pathway | 24 | 14 | 5 | 9 | 3.76E-5 |
| 0009063 | cellular amino acid catabolic process | 96 | 36 | 19 | 17 | 4.64E-5 |
| 0015995 | chlorophyll biosynthetic process | 30 | 16 | 6 | 10 | 4.75E-5 |
| 1901564 | organonitrogen compound metabolic process | 2034 | 473 | 404 | 69 | 4.78E-5 |
| 0010109 | regulation of photosynthesis | 36 | 18 | 7 | 11 | 5.00E-5 |
| 0009735 | response to cytokinin | 55 | 24 | 11 | 13 | 5.17E-5 |
| 0009410 | response to xenobiotic stimulus | 12 | 9 | 2 | 7 | 5.85E-5 |
| 0043090 | amino acid import | 6 | 6 | 1 | 5 | 6.13E-5 |
| 0043092 | L-amino acid import | 6 | 6 | 1 | 5 | 6.13E-5 |
| 0051938 | L-glutamate import | 6 | 6 | 1 | 5 | 6.13E-5 |
| 0006779 | porphyrin-containing compound biosynthetic process | 49 | 22 | 10 | 12 | 6.22E-5 |
| 0009814 | defense response, incompatible interaction | 49 | 22 | 10 | 12 | 6.22E-5 |
| 0032787 | monocarboxylic acid metabolic process | 571 | 151 | 113 | 38 | 7.06E-5 |
| 0009991 | response to extracellular stimulus | 105 | 38 | 21 | 17 | 7.14E-5 |
| 0050801 | ion homeostasis | 197 | 62 | 39 | 23 | 7.23E-5 |
| 0070588 | calcium ion transmembrane transport | 40 | 19 | 8 | 11 | 7.63E-5 |
| 0090693 | plant organ senescence | 40 | 19 | 8 | 11 | 7.63E-5 |
| 0016117 | carotenoid biosynthetic process | 28 | 15 | 6 | 9 | 7.66E-5 |
| 0016109 | tetraterpenoid biosynthetic process | 28 | 15 | 6 | 9 | 7.66E-5 |
| 0006094 | gluconeogenesis | 34 | 17 | 7 | 10 | 8.07E-5 |
| 0009694 | jasmonic acid metabolic process | 47 | 21 | 9 | 12 | 9.91E-5 |
| 0051289 | protein homotetramerization | 15 | 10 | 3 | 7 | 1.06E-4 |
| 0006066 | alcohol metabolic process | 118 | 41 | 23 | 18 | 1.09E-4 |
| 0010119 | regulation of stomatal movement | 38 | 18 | 8 | 10 | 1.23E-4 |
| 0019319 | hexose biosynthetic process | 35 | 17 | 7 | 10 | 1.28E-4 |
| 0009313 | oligosaccharide catabolic process | 32 | 16 | 6 | 10 | 1.31E-4 |
| 0046352 | disaccharide catabolic process | 32 | 16 | 6 | 10 | 1.31E-4 |
| 0051186 | cofactor metabolic process | 556 | 146 | 110 | 36 | 1.31E-4 |
| 0006816 | calcium ion transport | 48 | 21 | 10 | 11 | 1.43E-4 |
| 0009773 | photosynthetic electron transport in photosystem I | 13 | 9 | 3 | 6 | 1.56E-4 |
| 0044282 | small molecule catabolic process | 288 | 83 | 57 | 26 | 1.60E-4 |
| 0045490 | pectin catabolic process | 55 | 23 | 11 | 12 | 1.62E-4 |
| 0010150 | leaf senescence | 39 | 18 | 8 | 10 | 1.86E-4 |
| 1901000 | regulation of response to salt stress | 39 | 18 | 8 | 10 | 1.86E-4 |
| 0098662 | inorganic cation transmembrane transport | 558 | 145 | 111 | 34 | 2.30E-4 |
| 0046513 | ceramide biosynthetic process | 16 | 10 | 3 | 7 | 2.32E-4 |
| 0033014 | tetrapyrrole biosynthetic process | 53 | 22 | 11 | 11 | 2.54E-4 |
| 1903826 | arginine transmembrane transport | 9 | 7 | 2 | 5 | 2.99E-4 |
| 0036092 | phosphatidylinositol-3-phosphate biosynthetic process | 9 | 7 | 2 | 5 | 2.99E-4 |
| 0015976 | carbon utilization | 9 | 7 | 2 | 5 | 2.99E-4 |
| 0015809 | arginine transport | 9 | 7 | 2 | 5 | 2.99E-4 |
| 0019288 | isopentenyl diphosphate biosynthetic process, methylerythritol 4-phosphate pathway | 9 | 7 | 2 | 5 | 2.99E-4 |
| 1902644 | tertiary alcohol metabolic process | 22 | 12 | 4 | 8 | 3.25E-4 |
| 0009687 | abscisic acid metabolic process | 22 | 12 | 4 | 8 | 3.25E-4 |
| 0046246 | terpene biosynthetic process | 22 | 12 | 4 | 8 | 3.25E-4 |
| 1901420 | negative regulation of response to alcohol | 22 | 12 | 4 | 8 | 3.25E-4 |
| 0043288 | apocarotenoid metabolic process | 22 | 12 | 4 | 8 | 3.25E-4 |
| 0009937 | regulation of gibberellic acid mediated signaling pathway | 22 | 12 | 4 | 8 | 3.25E-4 |
| 0009788 | negative regulation of abscisic acid-activated signaling pathway | 22 | 12 | 4 | 8 | 3.25E-4 |
| 0009637 | response to blue light | 44 | 19 | 9 | 10 | 3.61E-4 |
| 0046364 | monosaccharide biosynthetic process | 44 | 19 | 9 | 10 | 3.61E-4 |
| 0033993 | response to lipid | 192 | 58 | 38 | 20 | 4.02E-4 |
| 0010648 | negative regulation of cell communication | 58 | 23 | 12 | 11 | 4.11E-4 |
| 0023057 | negative regulation of signaling | 58 | 23 | 12 | 11 | 4.11E-4 |
| 0009968 | negative regulation of signal transduction | 58 | 23 | 12 | 11 | 4.11E-4 |
| 0007568 | aging | 48 | 20 | 10 | 10 | 4.51E-4 |
| 0044092 | negative regulation of molecular function | 193 | 58 | 38 | 20 | 4.65E-4 |
| 0018298 | protein-chromophore linkage | 35 | 16 | 7 | 9 | 4.80E-4 |
| 0031667 | response to nutrient levels | 95 | 33 | 19 | 14 | 4.91E-4 |
| 0009064 | glutamine family amino acid metabolic process | 122 | 40 | 24 | 16 | 5.18E-4 |
| 0006559 | L-phenylalanine catabolic process | 20 | 11 | 4 | 7 | 5.27E-4 |
| 1902222 | erythrose 4-phosphate/phosphoenolpyruvate family amino acid catabolic process | 20 | 11 | 4 | 7 | 5.27E-4 |
| 0043647 | inositol phosphate metabolic process | 29 | 14 | 6 | 8 | 5.44E-4 |
| 0009767 | photosynthetic electron transport chain | 29 | 14 | 6 | 8 | 5.44E-4 |
| 0000272 | polysaccharide catabolic process | 186 | 56 | 37 | 19 | 5.48E-4 |
| 0009074 | aromatic amino acid family catabolic process | 26 | 13 | 5 | 8 | 5.59E-4 |
| 0009751 | response to salicylic acid | 42 | 18 | 8 | 10 | 5.72E-4 |
| 0044267 | cellular protein metabolic process | 4807 | 1036 | 955 | 81 | 6.47E-4 |
| 0006950 | response to stress | 2245 | 505 | 446 | 59 | 6.79E-4 |
| 0016116 | carotenoid metabolic process | 36 | 16 | 7 | 9 | 7.06E-4 |
| 0016108 | tetraterpenoid metabolic process | 36 | 16 | 7 | 9 | 7.06E-4 |
| 0098771 | inorganic ion homeostasis | 176 | 53 | 35 | 18 | 7.54E-4 |
| 0016052 | carbohydrate catabolic process | 379 | 101 | 75 | 26 | 7.75E-4 |
| 0016101 | diterpenoid metabolic process | 43 | 18 | 9 | 9 | 8.03E-4 |
| 0042214 | terpene metabolic process | 43 | 18 | 9 | 9 | 8.03E-4 |
| 0048583 | regulation of response to stimulus | 445 | 116 | 88 | 28 | 8.10E-4 |
| 0046395 | carboxylic acid catabolic process | 185 | 55 | 37 | 18 | 8.50E-4 |
| 0016054 | organic acid catabolic process | 185 | 55 | 37 | 18 | 8.50E-4 |
| 1902584 | positive regulation of response to water deprivation | 18 | 10 | 4 | 6 | 8.57E-4 |
| 0050789 | regulation of biological process | 5189 | 1112 | 1031 | 81 | 8.66E-4 |
| 0009768 | photosynthesis, light harvesting in photosystem I | 21 | 11 | 4 | 7 | 9.09E-4 |
| 2000070 | regulation of response to water deprivation | 24 | 12 | 5 | 7 | 9.13E-4 |
| 0018958 | phenol-containing compound metabolic process | 37 | 16 | 7 | 9 | 1.02E-3 |
| 0055080 | cation homeostasis | 170 | 51 | 34 | 17 | 1.03E-3 |
| 0042594 | response to starvation | 80 | 28 | 16 | 12 | 1.11E-3 |
| 0006006 | glucose metabolic process | 80 | 28 | 16 | 12 | 1.11E-3 |
| 0047484 | regulation of response to osmotic stress | 44 | 18 | 9 | 9 | 1.11E-3 |
| 0043086 | negative regulation of catalytic activity | 187 | 55 | 37 | 18 | 1.12E-3 |
| 0045493 | xylan catabolic process | 34 | 15 | 7 | 8 | 1.13E-3 |
| 0051291 | protein heterooligomerization | 8 | 6 | 2 | 4 | 1.18E-3 |
| 0072488 | ammonium transmembrane transport | 13 | 8 | 3 | 5 | 1.18E-3 |
| 0015696 | ammonium transport | 13 | 8 | 3 | 5 | 1.18E-3 |
| 0009409 | response to cold | 163 | 49 | 32 | 17 | 1.21E-3 |
| 0006714 | sesquiterpenoid metabolic process | 31 | 14 | 6 | 8 | 1.25E-3 |
| 0080134 | regulation of response to stress | 238 | 67 | 47 | 20 | 1.27E-3 |
| 0006520 | cellular amino acid metabolic process | 630 | 156 | 125 | 31 | 1.36E-3 |
| 0006665 | sphingolipid metabolic process | 81 | 28 | 16 | 12 | 1.38E-3 |
| 0009938 | negative regulation of gibberellic acid mediated signaling pathway | 16 | 9 | 3 | 6 | 1.40E-3 |
| 0043467 | regulation of generation of precursor metabolites and energy | 16 | 9 | 3 | 6 | 1.40E-3 |
| 0051187 | cofactor catabolic process | 16 | 9 | 3 | 6 | 1.40E-3 |
| 0097305 | response to alcohol | 160 | 48 | 32 | 16 | 1.42E-3 |
| 0009765 | photosynthesis, light harvesting | 22 | 11 | 4 | 7 | 1.49E-3 |
| 0051290 | protein heterotetramerization | 6 | 5 | 1 | 4 | 1.55E-3 |
| 0080022 | primary root development | 6 | 5 | 1 | 4 | 1.55E-3 |
| 1905156 | negative regulation of photosynthesis | 6 | 5 | 1 | 4 | 1.55E-3 |
| 0010438 | cellular response to sulfur starvation | 4 | 4 | 1 | 3 | 1.56E-3 |
| 0043155 | negative regulation of photosynthesis, light reaction | 4 | 4 | 1 | 3 | 1.56E-3 |
| 0010205 | photoinhibition | 4 | 4 | 1 | 3 | 1.56E-3 |
| 0015669 | gas transport | 4 | 4 | 1 | 3 | 1.56E-3 |
| 0015671 | oxygen transport | 4 | 4 | 1 | 3 | 1.56E-3 |
| 0071702 | organic substance transport | 1692 | 384 | 336 | 48 | 1.59E-3 |
| 0034754 | cellular hormone metabolic process | 42 | 17 | 8 | 9 | 1.74E-3 |
| 0009723 | response to ethylene | 42 | 17 | 8 | 9 | 1.74E-3 |
| 0009685 | gibberellin metabolic process | 32 | 14 | 6 | 8 | 1.81E-3 |
| 1901071 | glucosamine-containing compound metabolic process | 32 | 14 | 6 | 8 | 1.81E-3 |
| 0042548 | regulation of photosynthesis, light reaction | 11 | 7 | 2 | 5 | 1.88E-3 |
| 0046473 | phosphatidic acid metabolic process | 11 | 7 | 2 | 5 | 1.88E-3 |
| 0009737 | response to abscisic acid | 158 | 47 | 31 | 16 | 1.91E-3 |
| 0009072 | aromatic amino acid family metabolic process | 98 | 32 | 19 | 13 | 1.92E-3 |
| 0007167 | enzyme linked receptor protein signaling pathway | 276 | 75 | 55 | 20 | 1.96E-3 |
| 0007178 | transmembrane receptor protein serine/threonine kinase signaling pathway | 276 | 75 | 55 | 20 | 1.96E-3 |
| 0006558 | L-phenylalanine metabolic process | 29 | 13 | 6 | 7 | 2.01E-3 |
| 1902221 | erythrose 4-phosphate/phosphoenolpyruvate family amino acid metabolic process | 29 | 13 | 6 | 7 | 2.01E-3 |
| 0007154 | cell communication | 83 | 28 | 16 | 12 | 2.09E-3 |
| 0097164 | ammonium ion metabolic process | 68 | 24 | 14 | 10 | 2.14E-3 |
| 0051260 | protein homooligomerization | 26 | 12 | 5 | 7 | 2.19E-3 |
| 0009690 | cytokinin metabolic process | 36 | 15 | 7 | 8 | 2.27E-3 |
| 0030242 | pexophagy | 14 | 8 | 3 | 5 | 2.28E-3 |
| 0006560 | proline metabolic process | 14 | 8 | 3 | 5 | 2.28E-3 |
| 0046149 | pigment catabolic process | 14 | 8 | 3 | 5 | 2.28E-3 |
| 0033015 | tetrapyrrole catabolic process | 14 | 8 | 3 | 5 | 2.28E-3 |
| 0015996 | chlorophyll catabolic process | 14 | 8 | 3 | 5 | 2.28E-3 |
| 0006787 | porphyrin-containing compound catabolic process | 14 | 8 | 3 | 5 | 2.28E-3 |
| 0006641 | triglyceride metabolic process | 23 | 11 | 5 | 6 | 2.35E-3 |
| 0009065 | glutamine family amino acid catabolic process | 23 | 11 | 5 | 6 | 2.35E-3 |
| 1902645 | tertiary alcohol biosynthetic process | 17 | 9 | 3 | 6 | 2.45E-3 |
| 0009688 | abscisic acid biosynthetic process | 17 | 9 | 3 | 6 | 2.45E-3 |
| 0008272 | sulfate transport | 17 | 9 | 3 | 6 | 2.45E-3 |
| 0043289 | apocarotenoid biosynthetic process | 17 | 9 | 3 | 6 | 2.45E-3 |
| 0010200 | response to chitin | 17 | 9 | 3 | 6 | 2.45E-3 |
| 1902358 | sulfate transmembrane transport | 17 | 9 | 3 | 6 | 2.45E-3 |
| 0005986 | sucrose biosynthetic process | 20 | 10 | 4 | 6 | 2.45E-3 |
| 0050794 | regulation of cellular process | 4745 | 1013 | 943 | 70 | 2.50E-3 |
| 0009698 | phenylpropanoid metabolic process | 96 | 31 | 19 | 12 | 2.72E-3 |
| 0072511 | divalent inorganic cation transport | 100 | 32 | 20 | 12 | 2.75E-3 |
| 0070838 | divalent metal ion transport | 100 | 32 | 20 | 12 | 2.75E-3 |
| 0009733 | response to auxin | 108 | 34 | 21 | 13 | 2.80E-3 |
| 0018105 | peptidyl-serine phosphorylation | 120 | 37 | 24 | 13 | 2.80E-3 |
| 0019725 | cellular homeostasis | 323 | 85 | 64 | 21 | 2.81E-3 |
| 0046855 | inositol phosphate dephosphorylation | 9 | 6 | 2 | 4 | 2.95E-3 |
| 0015917 | aminophospholipid transport | 9 | 6 | 2 | 4 | 2.95E-3 |
| 0015804 | neutral amino acid transport | 9 | 6 | 2 | 4 | 2.95E-3 |
| 0015749 | monosaccharide transport | 9 | 6 | 2 | 4 | 2.95E-3 |
| 0071545 | inositol phosphate catabolic process | 9 | 6 | 2 | 4 | 2.95E-3 |
| 0030388 | fructose 1,6-bisphosphate metabolic process | 9 | 6 | 2 | 4 | 2.95E-3 |
| 0044106 | cellular amine metabolic process | 62 | 22 | 12 | 10 | 2.97E-3 |
| 0006576 | cellular biogenic amine metabolic process | 62 | 22 | 12 | 10 | 2.97E-3 |
| 0044272 | sulfur compound biosynthetic process | 145 | 43 | 29 | 14 | 3.08E-3 |
| 0055065 | metal ion homeostasis | 93 | 30 | 18 | 12 | 3.21E-3 |
| 0018209 | peptidyl-serine modification | 121 | 37 | 24 | 13 | 3.28E-3 |
| 0071496 | cellular response to external stimulus | 74 | 25 | 15 | 10 | 3.44E-3 |
| 0031668 | cellular response to extracellular stimulus | 74 | 25 | 15 | 10 | 3.44E-3 |
| 0042545 | cell wall modification | 48 | 18 | 10 | 8 | 3.50E-3 |
| 0009696 | salicylic acid metabolic process | 24 | 11 | 5 | 6 | 3.57E-3 |
| 0043620 | regulation of DNA-templated transcription in response to stress | 34 | 14 | 7 | 7 | 3.59E-3 |
| 0006536 | glutamate metabolic process | 34 | 14 | 7 | 7 | 3.59E-3 |
| 0042330 | taxis | 12 | 7 | 2 | 5 | 3.74E-3 |
| 0050918 | positive chemotaxis | 12 | 7 | 2 | 5 | 3.74E-3 |
| 0006935 | chemotaxis | 12 | 7 | 2 | 5 | 3.74E-3 |
| 0010183 | pollen tube guidance | 12 | 7 | 2 | 5 | 3.74E-3 |
| 0030522 | intracellular receptor signaling pathway | 12 | 7 | 2 | 5 | 3.74E-3 |
| 0009785 | blue light signaling pathway | 12 | 7 | 2 | 5 | 3.74E-3 |
| 0042445 | hormone metabolic process | 126 | 38 | 25 | 13 | 3.79E-3 |
| 0042430 | indole-containing compound metabolic process | 52 | 19 | 10 | 9 | 3.84E-3 |
| 0010229 | inflorescence development | 21 | 10 | 4 | 6 | 3.85E-3 |
| 0090567 | reproductive shoot system development | 21 | 10 | 4 | 6 | 3.85E-3 |
| 1901607 | alpha-amino acid biosynthetic process | 231 | 63 | 46 | 17 | 3.89E-3 |
| 0046834 | lipid phosphorylation | 67 | 23 | 13 | 10 | 3.91E-3 |
| 0006970 | response to osmotic stress | 214 | 59 | 43 | 16 | 3.94E-3 |
| 0043455 | regulation of secondary metabolic process | 18 | 9 | 4 | 5 | 4.04E-3 |
| 0031163 | metallo-sulfur cluster assembly | 45 | 17 | 9 | 8 | 4.10E-3 |
| 0016226 | iron-sulfur cluster assembly | 45 | 17 | 9 | 8 | 4.10E-3 |
| 0006835 | dicarboxylic acid transport | 31 | 13 | 6 | 7 | 4.10E-3 |
| 1901002 | positive regulation of response to salt stress | 31 | 13 | 6 | 7 | 4.10E-3 |
| 0008652 | cellular amino acid biosynthetic process | 275 | 73 | 55 | 18 | 4.22E-3 |
| 0048511 | rhythmic process | 38 | 15 | 8 | 7 | 4.23E-3 |
| 0042435 | indole-containing compound biosynthetic process | 38 | 15 | 8 | 7 | 4.23E-3 |
| 1905037 | autophagosome organization | 38 | 15 | 8 | 7 | 4.23E-3 |
| 0007623 | circadian rhythm | 38 | 15 | 8 | 7 | 4.23E-3 |
| 0000045 | autophagosome assembly | 38 | 15 | 8 | 7 | 4.23E-3 |
| 0007166 | cell surface receptor signaling pathway | 475 | 118 | 94 | 24 | 4.34E-3 |
| 0045488 | pectin metabolic process | 91 | 29 | 18 | 11 | 4.52E-3 |
| 0010393 | galacturonan metabolic process | 91 | 29 | 18 | 11 | 4.52E-3 |
| 0006527 | arginine catabolic process | 7 | 5 | 1 | 4 | 4.52E-3 |
| 0015808 | L-alanine transport | 7 | 5 | 1 | 4 | 4.52E-3 |
| 0015812 | gamma-aminobutyric acid transport | 7 | 5 | 1 | 4 | 4.52E-3 |
| 0032328 | alanine transport | 7 | 5 | 1 | 4 | 4.52E-3 |
| 1901001 | negative regulation of response to salt stress | 7 | 5 | 1 | 4 | 4.52E-3 |
| 0006586 | indolalkylamine metabolic process | 35 | 14 | 7 | 7 | 4.91E-3 |
| 0006568 | tryptophan metabolic process | 35 | 14 | 7 | 7 | 4.91E-3 |
| 0055082 | cellular chemical homeostasis | 136 | 40 | 27 | 13 | 4.91E-3 |
| 1901617 | organic hydroxy compound biosynthetic process | 195 | 54 | 39 | 15 | 5.10E-3 |
| 0015672 | monovalent inorganic cation transport | 456 | 113 | 91 | 22 | 5.59E-3 |
| 0072348 | sulfur compound transport | 39 | 15 | 8 | 7 | 5.63E-3 |
| 0043618 | regulation of transcription from RNA polymerase II promoter in response to stress | 32 | 13 | 6 | 7 | 5.67E-3 |
| 0046486 | glycerolipid metabolic process | 327 | 84 | 65 | 19 | 5.89E-3 |
| 0044262 | cellular carbohydrate metabolic process | 525 | 128 | 104 | 24 | 6.00E-3 |
| 0007033 | vacuole organization | 73 | 24 | 15 | 9 | 6.04E-3 |
| 1901136 | carbohydrate derivative catabolic process | 73 | 24 | 15 | 9 | 6.04E-3 |
| 0032544 | plastid translation | 10 | 6 | 2 | 4 | 6.14E-3 |
| 0006875 | cellular metal ion homeostasis | 54 | 19 | 11 | 8 | 6.16E-3 |
| 0006672 | ceramide metabolic process | 43 | 16 | 9 | 7 | 6.27E-3 |
| 0006750 | glutathione biosynthetic process | 19 | 9 | 4 | 5 | 6.34E-3 |
| 0010555 | response to mannitol | 5 | 4 | 1 | 3 | 6.54E-3 |
| 0045117 | azole transport | 5 | 4 | 1 | 3 | 6.54E-3 |
| 0031221 | arabinan metabolic process | 16 | 8 | 3 | 5 | 6.69E-3 |
| 0031222 | arabinan catabolic process | 16 | 8 | 3 | 5 | 6.69E-3 |
| 0046838 | phosphorylated carbohydrate dephosphorylation | 13 | 7 | 3 | 4 | 6.72E-3 |
| 0010325 | raffinose family oligosaccharide biosynthetic process | 13 | 7 | 3 | 4 | 6.72E-3 |
| 0071483 | cellular response to blue light | 13 | 7 | 3 | 4 | 6.72E-3 |
| 0016567 | protein ubiquitination | 706 | 167 | 140 | 27 | 6.80E-3 |
| 0046165 | alcohol biosynthetic process | 70 | 23 | 14 | 9 | 7.16E-3 |
| 0042886 | amide transport | 160 | 45 | 32 | 13 | 7.36E-3 |
| 0006081 | cellular aldehyde metabolic process | 102 | 31 | 20 | 11 | 7.39E-3 |
| 0016106 | sesquiterpenoid biosynthetic process | 26 | 11 | 5 | 6 | 7.48E-3 |
| 0065008 | regulation of biological quality | 993 | 228 | 197 | 31 | 7.78E-3 |
| 0006593 | ornithine catabolic process | 3 | 3 | 1 | 2 | 7.84E-3 |
| 0031540 | regulation of anthocyanin biosynthetic process | 3 | 3 | 1 | 2 | 7.84E-3 |
| 0043048 | dolichyl monophosphate biosynthetic process | 3 | 3 | 1 | 2 | 7.84E-3 |
| 0009413 | response to flooding | 3 | 3 | 1 | 2 | 7.84E-3 |
| 0051560 | mitochondrial calcium ion homeostasis | 3 | 3 | 1 | 2 | 7.84E-3 |
| 0010304 | PSII associated light-harvesting complex II catabolic process | 3 | 3 | 1 | 2 | 7.84E-3 |
| 0019544 | arginine catabolic process to glutamate | 3 | 3 | 1 | 2 | 7.84E-3 |
| 0019493 | arginine catabolic process to proline | 3 | 3 | 1 | 2 | 7.84E-3 |
| 0010121 | arginine catabolic process to proline via ornithine | 3 | 3 | 1 | 2 | 7.84E-3 |
| 0009962 | regulation of flavonoid biosynthetic process | 3 | 3 | 1 | 2 | 7.84E-3 |
| 0009970 | cellular response to sulfate starvation | 3 | 3 | 1 | 2 | 7.84E-3 |
| 0010047 | fruit dehiscence | 3 | 3 | 1 | 2 | 7.84E-3 |
| 0030258 | lipid modification | 199 | 54 | 40 | 14 | 7.89E-3 |
| 0048580 | regulation of post-embryonic development | 165 | 46 | 33 | 13 | 8.06E-3 |
| 0008219 | cell death | 48 | 17 | 10 | 7 | 8.65E-3 |
| 0006638 | neutral lipid metabolic process | 37 | 14 | 7 | 7 | 8.69E-3 |
| 0006639 | acylglycerol metabolic process | 37 | 14 | 7 | 7 | 8.69E-3 |
| 0009755 | hormone-mediated signaling pathway | 341 | 86 | 68 | 18 | 8.91E-3 |
| 0006873 | cellular ion homeostasis | 120 | 35 | 24 | 11 | 9.32E-3 |
| 0035672 | oligopeptide transmembrane transport | 116 | 34 | 23 | 11 | 9.49E-3 |
| 0042398 | cellular modified amino acid biosynthetic process | 56 | 19 | 11 | 8 | 9.52E-3 |
| 0009642 | response to light intensity | 60 | 20 | 12 | 8 | 9.82E-3 |
| 0031537 | regulation of anthocyanin metabolic process | 8 | 5 | 2 | 3 | 0.01 |
| 0051762 | sesquiterpene biosynthetic process | 8 | 5 | 2 | 3 | 0.01 |
| 0051761 | sesquiterpene metabolic process | 8 | 5 | 2 | 3 | 0.01 |
| 1900376 | regulation of secondary metabolite biosynthetic process | 8 | 5 | 2 | 3 | 0.01 |
| 0016123 | xanthophyll biosynthetic process | 8 | 5 | 2 | 3 | 0.01 |
| 0048582 | positive regulation of post-embryonic development | 27 | 11 | 5 | 6 | 0.01 |
| 0031407 | oxylipin metabolic process | 27 | 11 | 5 | 6 | 0.01 |
| 0031408 | oxylipin biosynthetic process | 27 | 11 | 5 | 6 | 0.01 |
| 0048364 | root development | 27 | 11 | 5 | 6 | 0.01 |
| 0048017 | inositol lipid-mediated signaling | 17 | 8 | 3 | 5 | 0.01 |
| 0015851 | nucleobase transport | 17 | 8 | 3 | 5 | 0.01 |
| 0031347 | regulation of defense response | 121 | 35 | 24 | 11 | 0.01 |
| 0042592 | homeostatic process | 659 | 155 | 131 | 24 | 0.01 |
| 0009646 | response to absence of light | 14 | 7 | 3 | 4 | 0.01 |
| 0016120 | carotene biosynthetic process | 14 | 7 | 3 | 4 | 0.01 |
| 1901292 | nucleoside phosphate catabolic process | 11 | 6 | 2 | 4 | 0.01 |
| 0072330 | monocarboxylic acid biosynthetic process | 255 | 66 | 51 | 15 | 0.01 |
| 0044724 | single-organism carbohydrate catabolic process | 220 | 58 | 44 | 14 | 0.01 |
| 0009808 | lignin metabolic process | 61 | 20 | 12 | 8 | 0.01 |
| 0051240 | positive regulation of multicellular organismal process | 31 | 12 | 6 | 6 | 0.01 |
| 0036003 | positive regulation of transcription from RNA polymerase II promoter in response to stress | 31 | 12 | 6 | 6 | 0.01 |
| 0061408 | positive regulation of transcription from RNA polymerase II promoter in response to heat stress | 31 | 12 | 6 | 6 | 0.01 |
| 0030148 | sphingolipid biosynthetic process | 42 | 15 | 8 | 7 | 0.01 |
| 0006644 | phospholipid metabolic process | 368 | 91 | 73 | 18 | 0.01 |
| 0042401 | cellular biogenic amine biosynthetic process | 46 | 16 | 9 | 7 | 0.01 |
| 0009309 | amine biosynthetic process | 46 | 16 | 9 | 7 | 0.01 |
| 0044036 | cell wall macromolecule metabolic process | 204 | 54 | 41 | 13 | 0.01 |
| 0015698 | inorganic anion transport | 148 | 41 | 29 | 12 | 0.01 |
| 0016119 | carotene metabolic process | 35 | 13 | 7 | 6 | 0.01 |
| 0046854 | phosphatidylinositol phosphorylation | 50 | 17 | 10 | 7 | 0.01 |
| 0023051 | regulation of signaling | 200 | 53 | 40 | 13 | 0.01 |
| 0009966 | regulation of signal transduction | 200 | 53 | 40 | 13 | 0.01 |
| 0022900 | electron transport chain | 293 | 74 | 58 | 16 | 0.01 |
| 0009267 | cellular response to starvation | 62 | 20 | 12 | 8 | 0.01 |
| 0042180 | cellular ketone metabolic process | 78 | 24 | 15 | 9 | 0.01 |
| 0044248 | cellular catabolic process | 1432 | 317 | 284 | 33 | 0.02 |
| 0009240 | isopentenyl diphosphate biosynthetic process | 18 | 8 | 4 | 4 | 0.02 |
| 0046490 | isopentenyl diphosphate metabolic process | 18 | 8 | 4 | 4 | 0.02 |
| 0016998 | cell wall macromolecule catabolic process | 32 | 12 | 6 | 6 | 0.02 |
| 0000162 | tryptophan biosynthetic process | 25 | 10 | 5 | 5 | 0.02 |
| 0046219 | indolalkylamine biosynthetic process | 25 | 10 | 5 | 5 | 0.02 |
| 0009741 | response to brassinosteroid | 25 | 10 | 5 | 5 | 0.02 |
| 0002215 | defense response to nematode | 6 | 4 | 1 | 3 | 0.02 |
| 0010230 | alternative respiration | 6 | 4 | 1 | 3 | 0.02 |
| 0006044 | N-acetylglucosamine metabolic process | 6 | 4 | 1 | 3 | 0.02 |
| 0070189 | kynurenine metabolic process | 6 | 4 | 1 | 3 | 0.02 |
| 0019685 | photosynthesis, dark reaction | 15 | 7 | 3 | 4 | 0.02 |
| 0019253 | reductive pentose-phosphate cycle | 15 | 7 | 3 | 4 | 0.02 |
| 0019748 | secondary metabolic process | 155 | 42 | 31 | 11 | 0.02 |
| 0006091 | generation of precursor metabolites and energy | 483 | 115 | 96 | 19 | 0.02 |
| 0009668 | plastid membrane organization | 40 | 14 | 8 | 6 | 0.02 |
| 0010027 | thylakoid membrane organization | 40 | 14 | 8 | 6 | 0.02 |
| 0000041 | transition metal ion transport | 96 | 28 | 19 | 9 | 0.02 |
| 0099402 | plant organ development | 117 | 33 | 23 | 10 | 0.02 |
| 0006552 | leucine catabolic process | 12 | 6 | 2 | 4 | 0.02 |
| 0097428 | protein maturation by iron-sulfur cluster transfer | 12 | 6 | 2 | 4 | 0.02 |
| 0006654 | phosphatidic acid biosynthetic process | 9 | 5 | 2 | 3 | 0.02 |
| 0019048 | modulation by virus of host morphology or physiology | 9 | 5 | 2 | 3 | 0.02 |
| 0097577 | sequestering of iron ion | 9 | 5 | 2 | 3 | 0.02 |
| 0042631 | cellular response to water deprivation | 9 | 5 | 2 | 3 | 0.02 |
| 0071462 | cellular response to water stimulus | 9 | 5 | 2 | 3 | 0.02 |
| 0006880 | intracellular sequestering of iron ion | 9 | 5 | 2 | 3 | 0.02 |
| 0010037 | response to carbon dioxide | 9 | 5 | 2 | 3 | 0.02 |
| 0009862 | systemic acquired resistance, salicylic acid mediated signaling pathway | 9 | 5 | 2 | 3 | 0.02 |
| 0009854 | oxidative photosynthetic carbon pathway | 9 | 5 | 2 | 3 | 0.02 |
| 0019682 | glyceraldehyde-3-phosphate metabolic process | 44 | 15 | 9 | 6 | 0.02 |
| 0019184 | nonribosomal peptide biosynthetic process | 22 | 9 | 4 | 5 | 0.02 |
| 0044723 | single-organism carbohydrate metabolic process | 755 | 173 | 150 | 23 | 0.02 |
| 0010646 | regulation of cell communication | 204 | 53 | 41 | 12 | 0.02 |
| 0016042 | lipid catabolic process | 253 | 64 | 50 | 14 | 0.02 |
| 0046164 | alcohol catabolic process | 33 | 12 | 7 | 5 | 0.02 |
| 0006144 | purine nucleobase metabolic process | 33 | 12 | 7 | 5 | 0.02 |
| 0006744 | ubiquinone biosynthetic process | 33 | 12 | 7 | 5 | 0.02 |
| 0006743 | ubiquinone metabolic process | 33 | 12 | 7 | 5 | 0.02 |
| 0006541 | glutamine metabolic process | 60 | 19 | 12 | 7 | 0.02 |
| 0006766 | vitamin metabolic process | 97 | 28 | 19 | 9 | 0.02 |
| 0032446 | protein modification by small protein conjugation | 757 | 173 | 150 | 23 | 0.02 |
| 0055067 | monovalent inorganic cation homeostasis | 93 | 27 | 18 | 9 | 0.02 |
| 0006002 | fructose 6-phosphate metabolic process | 37 | 13 | 7 | 6 | 0.02 |
| 0006090 | pyruvate metabolic process | 144 | 39 | 29 | 10 | 0.02 |
| 0046348 | amino sugar catabolic process | 26 | 10 | 5 | 5 | 0.02 |
| 0006030 | chitin metabolic process | 26 | 10 | 5 | 5 | 0.02 |
| 0006032 | chitin catabolic process | 26 | 10 | 5 | 5 | 0.02 |
| 0006026 | aminoglycan catabolic process | 26 | 10 | 5 | 5 | 0.02 |
| 1901072 | glucosamine-containing compound catabolic process | 26 | 10 | 5 | 5 | 0.02 |
| 1901259 | chloroplast rRNA processing | 19 | 8 | 4 | 4 | 0.02 |
| 0006995 | cellular response to nitrogen starvation | 19 | 8 | 4 | 4 | 0.02 |
| 0006000 | fructose metabolic process | 19 | 8 | 4 | 4 | 0.02 |
| 0009651 | response to salt stress | 188 | 49 | 37 | 12 | 0.02 |
| 0055088 | lipid homeostasis | 65 | 20 | 13 | 7 | 0.02 |
| 0031669 | cellular response to nutrient levels | 65 | 20 | 13 | 7 | 0.02 |
| 0042044 | fluid transport | 30 | 11 | 6 | 5 | 0.02 |
| 0006833 | water transport | 30 | 11 | 6 | 5 | 0.02 |
| 0010951 | negative regulation of endopeptidase activity | 57 | 18 | 11 | 7 | 0.02 |
| 0048506 | regulation of timing of meristematic phase transition | 16 | 7 | 3 | 4 | 0.03 |
| 0048510 | regulation of timing of transition from vegetative to reproductive phase | 16 | 7 | 3 | 4 | 0.03 |
| 0052646 | alditol phosphate metabolic process | 16 | 7 | 3 | 4 | 0.03 |
| 0019740 | nitrogen utilization | 16 | 7 | 3 | 4 | 0.03 |
| 0006072 | glycerol-3-phosphate metabolic process | 16 | 7 | 3 | 4 | 0.03 |
| 0055072 | iron ion homeostasis | 34 | 12 | 7 | 5 | 0.03 |
| 0051094 | positive regulation of developmental process | 34 | 12 | 7 | 5 | 0.03 |
| 0010207 | photosystem II assembly | 23 | 9 | 5 | 4 | 0.03 |
| 0006108 | malate metabolic process | 23 | 9 | 5 | 4 | 0.03 |
| 0006826 | iron ion transport | 23 | 9 | 5 | 4 | 0.03 |
| 0009108 | coenzyme biosynthetic process | 194 | 50 | 39 | 11 | 0.03 |
| 0019637 | organophosphate metabolic process | 894 | 201 | 178 | 23 | 0.03 |
| 0006562 | proline catabolic process | 4 | 3 | 1 | 2 | 0.03 |
| 0062034 | --- | 4 | 3 | 1 | 2 | 0.03 |
| 0030970 | retrograde protein transport, ER to cytosol | 4 | 3 | 1 | 2 | 0.03 |
| 0046951 | ketone body biosynthetic process | 4 | 3 | 1 | 2 | 0.03 |
| 0046950 | cellular ketone body metabolic process | 4 | 3 | 1 | 2 | 0.03 |
| 0043953 | protein transport by the Tat complex | 4 | 3 | 1 | 2 | 0.03 |
| 0019500 | cyanide catabolic process | 4 | 3 | 1 | 2 | 0.03 |
| 1903513 | endoplasmic reticulum to cytosol transport | 4 | 3 | 1 | 2 | 0.03 |
| 1902224 | ketone body metabolic process | 4 | 3 | 1 | 2 | 0.03 |
| 0010133 | proline catabolic process to glutamate | 4 | 3 | 1 | 2 | 0.03 |
| 0009875 | pollen-pistil interaction | 4 | 3 | 1 | 2 | 0.03 |
| 0097352 | autophagosome maturation | 4 | 3 | 1 | 2 | 0.03 |
| 0051228 | mitotic spindle disassembly | 4 | 3 | 1 | 2 | 0.03 |
| 0051230 | spindle disassembly | 4 | 3 | 1 | 2 | 0.03 |
| 0015833 | peptide transport | 129 | 35 | 26 | 9 | 0.03 |
| 0006857 | oligopeptide transport | 129 | 35 | 26 | 9 | 0.03 |
| 0009112 | nucleobase metabolic process | 66 | 20 | 13 | 7 | 0.03 |
| 0071229 | cellular response to acid chemical | 58 | 18 | 12 | 6 | 0.03 |
| 0052548 | regulation of endopeptidase activity | 58 | 18 | 12 | 6 | 0.03 |
| 0031098 | stress-activated protein kinase signaling cascade | 54 | 17 | 11 | 6 | 0.03 |
| 0009812 | flavonoid metabolic process | 27 | 10 | 5 | 5 | 0.03 |
| 0010204 | defense response signaling pathway, resistance gene-independent | 13 | 6 | 3 | 3 | 0.03 |
| 0043648 | dicarboxylic acid metabolic process | 121 | 33 | 24 | 9 | 0.03 |
| 0009110 | vitamin biosynthetic process | 83 | 24 | 16 | 8 | 0.03 |
| 0040034 | regulation of development, heterochronic | 20 | 8 | 4 | 4 | 0.03 |
| 0051239 | regulation of multicellular organismal process | 245 | 61 | 49 | 12 | 0.03 |
| 0006561 | proline biosynthetic process | 10 | 5 | 2 | 3 | 0.03 |
| 0019755 | one-carbon compound transport | 10 | 5 | 2 | 3 | 0.03 |
| 0046283 | anthocyanin-containing compound metabolic process | 10 | 5 | 2 | 3 | 0.03 |
| 0007584 | response to nutrient | 10 | 5 | 2 | 3 | 0.03 |
| 0015840 | urea transport | 10 | 5 | 2 | 3 | 0.03 |
| 0010016 | shoot system morphogenesis | 10 | 5 | 2 | 3 | 0.03 |
| 0010038 | response to metal ion | 152 | 40 | 30 | 10 | 0.03 |
| 0000302 | response to reactive oxygen species | 75 | 22 | 15 | 7 | 0.03 |
| 0006658 | phosphatidylserine metabolic process | 7 | 4 | 1 | 3 | 0.03 |
| 0006659 | phosphatidylserine biosynthetic process | 7 | 4 | 1 | 3 | 0.03 |
| 0008216 | spermidine metabolic process | 7 | 4 | 1 | 3 | 0.03 |
| 0009166 | nucleotide catabolic process | 7 | 4 | 1 | 3 | 0.03 |
| 0008645 | hexose transport | 7 | 4 | 1 | 3 | 0.03 |
| 0001174 | transcriptional start site selection at RNA polymerase II promoter | 7 | 4 | 1 | 3 | 0.03 |
| 0001173 | DNA-templated transcriptional start site selection | 7 | 4 | 1 | 3 | 0.03 |
| 0046503 | glycerolipid catabolic process | 7 | 4 | 1 | 3 | 0.03 |
| 0010030 | positive regulation of seed germination | 7 | 4 | 1 | 3 | 0.03 |
| 0071805 | potassium ion transmembrane transport | 109 | 30 | 22 | 8 | 0.03 |
| 0071804 | cellular potassium ion transport | 109 | 30 | 22 | 8 | 0.03 |
| 0044242 | cellular lipid catabolic process | 135 | 36 | 27 | 9 | 0.03 |
| 0009070 | serine family amino acid biosynthetic process | 63 | 19 | 13 | 6 | 0.03 |
| 0009699 | phenylpropanoid biosynthetic process | 55 | 17 | 11 | 6 | 0.03 |
| 0006040 | amino sugar metabolic process | 47 | 15 | 9 | 6 | 0.03 |
| 0009084 | glutamine family amino acid biosynthetic process | 51 | 16 | 10 | 6 | 0.03 |
| 0043412 | macromolecule modification | 4929 | 1025 | 979 | 46 | 0.04 |
| 0006885 | regulation of pH | 80 | 23 | 16 | 7 | 0.04 |
| 0010311 | lateral root formation | 17 | 7 | 3 | 4 | 0.04 |
| 0019432 | triglyceride biosynthetic process | 17 | 7 | 3 | 4 | 0.04 |
| 0006022 | aminoglycan metabolic process | 28 | 10 | 6 | 4 | 0.04 |
| 0006979 | response to oxidative stress | 316 | 76 | 63 | 13 | 0.04 |
| 0098661 | inorganic anion transmembrane transport | 68 | 20 | 14 | 6 | 0.04 |
| 0048731 | system development | 32 | 11 | 6 | 5 | 0.04 |
| 2000241 | regulation of reproductive process | 141 | 37 | 28 | 9 | 0.04 |
| 0044126 | regulation of growth of symbiont in host | 2 | 2 | 0 | 2 | 0.04 |
| 0044130 | negative regulation of growth of symbiont in host | 2 | 2 | 0 | 2 | 0.04 |
| 0033396 | beta-alanine biosynthetic process via 3-ureidopropionate | 2 | 2 | 0 | 2 | 0.04 |
| 0044144 | modulation of growth of symbiont involved in interaction with host | 2 | 2 | 0 | 2 | 0.04 |
| 0044146 | negative regulation of growth of symbiont involved in interaction with host | 2 | 2 | 0 | 2 | 0.04 |
| 0033354 | chlorophyll cycle | 2 | 2 | 0 | 2 | 0.04 |
| 0050992 | dimethylallyl diphosphate biosynthetic process | 2 | 2 | 0 | 2 | 0.04 |
| 0050993 | dimethylallyl diphosphate metabolic process | 2 | 2 | 0 | 2 | 0.04 |
| 0000304 | response to singlet oxygen | 2 | 2 | 0 | 2 | 0.04 |
| 0034486 | vacuolar transmembrane transport | 2 | 2 | 0 | 2 | 0.04 |
| 0055079 | aluminum ion homeostasis | 2 | 2 | 0 | 2 | 0.04 |
| 0055089 | fatty acid homeostasis | 2 | 2 | 0 | 2 | 0.04 |
| 0055090 | acylglycerol homeostasis | 2 | 2 | 0 | 2 | 0.04 |
| 0055091 | phospholipid homeostasis | 2 | 2 | 0 | 2 | 0.04 |
| 0010439 | regulation of glucosinolate biosynthetic process | 2 | 2 | 0 | 2 | 0.04 |
| 0031341 | regulation of cell killing | 2 | 2 | 0 | 2 | 0.04 |
| 0052865 | 1-deoxy-D-xylulose 5-phosphate biosynthetic process | 2 | 2 | 0 | 2 | 0.04 |
| 0031343 | positive regulation of cell killing | 2 | 2 | 0 | 2 | 0.04 |
| 0052863 | 1-deoxy-D-xylulose 5-phosphate metabolic process | 2 | 2 | 0 | 2 | 0.04 |
| 0019628 | urate catabolic process | 2 | 2 | 0 | 2 | 0.04 |
| 0080177 | plastoglobule organization | 2 | 2 | 0 | 2 | 0.04 |
| 0090414 | molybdate ion export from vacuole | 2 | 2 | 0 | 2 | 0.04 |
| 0051712 | positive regulation of killing of cells of other organism | 2 | 2 | 0 | 2 | 0.04 |
| 0051714 | positive regulation of cytolysis in other organism | 2 | 2 | 0 | 2 | 0.04 |
| 0051710 | regulation of cytolysis in other organism | 2 | 2 | 0 | 2 | 0.04 |
| 0051709 | regulation of killing of cells of other organism | 2 | 2 | 0 | 2 | 0.04 |
| 0010217 | cellular aluminum ion homeostasis | 2 | 2 | 0 | 2 | 0.04 |
| 0019482 | beta-alanine metabolic process | 2 | 2 | 0 | 2 | 0.04 |
| 0019483 | beta-alanine biosynthetic process | 2 | 2 | 0 | 2 | 0.04 |
| 0019424 | sulfide oxidation, using siroheme sulfite reductase | 2 | 2 | 0 | 2 | 0.04 |
| 0019418 | sulfide oxidation | 2 | 2 | 0 | 2 | 0.04 |
| 0045919 | positive regulation of cytolysis | 2 | 2 | 0 | 2 | 0.04 |
| 1902025 | nitrate import | 2 | 2 | 0 | 2 | 0.04 |
| 1902024 | L-histidine transport | 2 | 2 | 0 | 2 | 0.04 |
| 0090548 | response to nitrate starvation | 2 | 2 | 0 | 2 | 0.04 |
| 0046415 | urate metabolic process | 2 | 2 | 0 | 2 | 0.04 |
| 0051802 | regulation of cytolysis in other organism involved in symbiotic interaction | 2 | 2 | 0 | 2 | 0.04 |
| 0051804 | positive regulation of cytolysis in other organism involved in symbiotic interaction | 2 | 2 | 0 | 2 | 0.04 |
| 0015817 | histidine transport | 2 | 2 | 0 | 2 | 0.04 |
| 0051841 | positive regulation by host of cytolysis of symbiont cells | 2 | 2 | 0 | 2 | 0.04 |
| 0051839 | regulation by host of cytolysis of symbiont cells | 2 | 2 | 0 | 2 | 0.04 |
| 0006844 | acyl carnitine transport | 2 | 2 | 0 | 2 | 0.04 |
| 0015757 | galactose transport | 2 | 2 | 0 | 2 | 0.04 |
| 0033587 | shikimate biosynthetic process | 2 | 2 | 0 | 2 | 0.04 |
| 0089709 | L-histidine transmembrane transport | 2 | 2 | 0 | 2 | 0.04 |
| 0015697 | quaternary ammonium group transport | 2 | 2 | 0 | 2 | 0.04 |
| 0042268 | regulation of cytolysis | 2 | 2 | 0 | 2 | 0.04 |
| 0070328 | triglyceride homeostasis | 2 | 2 | 0 | 2 | 0.04 |
| 0006767 | water-soluble vitamin metabolic process | 85 | 24 | 17 | 7 | 0.04 |
| 0006813 | potassium ion transport | 115 | 31 | 23 | 8 | 0.04 |
| 1901661 | quinone metabolic process | 56 | 17 | 11 | 6 | 0.04 |
| 1901663 | quinone biosynthetic process | 56 | 17 | 11 | 6 | 0.04 |
| 0042181 | ketone biosynthetic process | 56 | 17 | 11 | 6 | 0.04 |
| 1901616 | organic hydroxy compound catabolic process | 40 | 13 | 8 | 5 | 0.04 |
| 0051259 | protein oligomerization | 52 | 16 | 10 | 6 | 0.04 |
| 0010337 | regulation of salicylic acid metabolic process | 21 | 8 | 4 | 4 | 0.04 |
| 0043562 | cellular response to nitrogen levels | 21 | 8 | 4 | 4 | 0.04 |
| 0009695 | jasmonic acid biosynthetic process | 14 | 6 | 3 | 3 | 0.04 |
| 0010256 | endomembrane system organization | 14 | 6 | 3 | 3 | 0.04 |
| 0010114 | response to red light | 14 | 6 | 3 | 3 | 0.04 |
| 0009863 | salicylic acid mediated signaling pathway | 14 | 6 | 3 | 3 | 0.04 |
| 0048831 | regulation of shoot system development | 133 | 35 | 26 | 9 | 0.04 |
| 0030003 | cellular cation homeostasis | 107 | 29 | 21 | 8 | 0.04 |
| 0023014 | signal transduction by protein phosphorylation | 69 | 20 | 14 | 6 | 0.04 |
| 0016051 | carbohydrate biosynthetic process | 361 | 85 | 72 | 13 | 0.05 |
| 0048367 | shoot system development | 29 | 10 | 6 | 4 | 0.05 |
| 0055076 | transition metal ion homeostasis | 57 | 17 | 11 | 6 | 0.05 |
| 0009266 | response to temperature stimulus | 329 | 78 | 65 | 13 | 0.05 |
| 0006650 | glycerophospholipid metabolic process | 283 | 68 | 56 | 12 | 0.05 |
| 1900140 | regulation of seedling development | 33 | 11 | 7 | 4 | 0.05 |
| 0010029 | regulation of seed germination | 33 | 11 | 7 | 4 | 0.05 |
| 0006591 | ornithine metabolic process | 11 | 5 | 2 | 3 | 0.05 |
| 0046168 | glycerol-3-phosphate catabolic process | 11 | 5 | 2 | 3 | 0.05 |
| 0009395 | phospholipid catabolic process | 11 | 5 | 2 | 3 | 0.05 |
| 0042724 | thiamine-containing compound biosynthetic process | 11 | 5 | 2 | 3 | 0.05 |
| 0010218 | response to far red light | 11 | 5 | 2 | 3 | 0.05 |
| 0009228 | thiamine biosynthetic process | 11 | 5 | 2 | 3 | 0.05 |
| 0009803 | cinnamic acid metabolic process | 11 | 5 | 2 | 3 | 0.05 |
| 0009800 | cinnamic acid biosynthetic process | 11 | 5 | 2 | 3 | 0.05 |
| 0002239 | response to oomycetes | 117 | 31 | 23 | 8 | 0.05 |
| 0002229 | defense response to oomycetes | 117 | 31 | 23 | 8 | 0.05 |
| 0046470 | phosphatidylcholine metabolic process | 41 | 13 | 8 | 5 | 0.05 |
| 0071482 | cellular response to light stimulus | 41 | 13 | 8 | 5 | 0.05 |
| 0080142 | regulation of salicylic acid biosynthetic process | 18 | 7 | 4 | 3 | 0.05 |
| 0046460 | neutral lipid biosynthetic process | 18 | 7 | 4 | 3 | 0.05 |
| 0046463 | acylglycerol biosynthetic process | 18 | 7 | 4 | 3 | 0.05 |
