# Supplementary material for: Reproductive developmental transcriptome analysis of Tripidium ravennae (Poaceae)
Source: BMC Genomics. 2021 Jun 28;22:483. doi: 10.1186/s12864-021-07641-y (PMC8237498; doi:10.1186/s12864-021-07641-y)
Supplement: Supplementary file 1 — Additional file 1: Table S1. Sequencing statistics. Figure S1a-c. Transcriptome assembly. Figure S2. Annotation statistics for primary de novo assembly. Figure S3. Annotation statistics for cluster enriched assembly. Figure S4. Annotation statistics for PB Iso-Seq sequences. Table S2. GO-term enrichment for upregulated transcripts during inflorescence development. Table S3. GO-term enrichment for upregulated transcripts during flower development. Table S4. GO-term enrichment for upregulated transcripts during seed development. Table S5. Excel workbook including summaries of DEG’s in inflorescence development. Table S6. Excel workbook including summaries of DEG’s in floral development. Table S7. Excel workbook including summaries of DEG’s in seed development. Supplemental List 1. List of FASTA formatted sequences associated with Fig. 8 and Tables 2, 3, and 4. Table S8. Table export of annotations for the cluster enriched de novo transcriptome assembly. Table S9. Table export of annotations for the collapsed Iso-seq transcript set. [file 12864_2021_7641_MOESM1_ESM.zip › ST4-GOEnrichAnalysisDuringSeedDvlpmt.docx]

**Reproductive developmental transcriptome analysis of *Tripidium ravennae* (Poaceae)**

Nathan Maren^1^*, Fangzhou Zhao^1,2^, Rishi Aryal^1^, Darren Touchell^3^, Wusheng Liu^1^, Thomas Ranney^3^, and Hamid Ashrafi^1*^

^1^Department of Horticultural Science, North Carolina State University, Campus Box 7609, Raleigh, NC 27695-7609, USA

^2^College of Agriculture, Nanjing Agricultural University, Nanjing 210095, China

^3^Mountain Crop Improvement Lab, Department of Horticultural Science, Mountain Horticultural Crops Research and Extension Center, North Carolina State University, 455 Research Drive, Mills River, NC 28759-3423, USA

*Corresponding authors: hamidashrafi@ncsu.edu and namaren@ncsu.edu

| **Category** | **Description** | **Full set** | **In subset** | **Expected in subset** | **Observed - expected** | **p-value** |
| --- | --- | --- | --- | --- | --- | --- |
| 0044699 | single-organism process | 9115 | 2079 | 1618 | 461 | 0.00 |
| 0044710 | single-organism metabolic process | 5645 | 1351 | 1002 | 349 | 0.00 |
| 0044711 | single-organism biosynthetic process | 1908 | 501 | 339 | 162 | 0.00 |
| 0071555 | cell wall organization | 332 | 129 | 59 | 70 | 0.00 |
| 0005975 | carbohydrate metabolic process | 1389 | 412 | 247 | 165 | 1.11E-16 |
| 0055114 | oxidation-reduction process | 2524 | 660 | 448 | 212 | 2.22E-16 |
| 0045229 | external encapsulating structure organization | 349 | 135 | 62 | 73 | 5.55E-16 |
| 0071554 | cell wall organization or biogenesis | 374 | 148 | 66 | 82 | 5.55E-16 |
| 0055085 | transmembrane transport | 2005 | 496 | 356 | 140 | 6.66E-16 |
| 0006091 | generation of precursor metabolites and energy | 483 | 165 | 86 | 79 | 7.77E-16 |
| 0006811 | ion transport | 1269 | 338 | 225 | 113 | 9.99E-16 |
| 0044763 | single-organism cellular process | 5600 | 1225 | 994 | 231 | 1.11E-15 |
| 0009768 | photosynthesis, light harvesting in photosystem I | 21 | 20 | 4 | 16 | 1.67E-14 |
| 0044723 | single-organism carbohydrate metabolic process | 755 | 218 | 134 | 84 | 1.74E-14 |
| 0009765 | photosynthesis, light harvesting | 22 | 20 | 4 | 16 | 1.50E-13 |
| 0005976 | polysaccharide metabolic process | 477 | 149 | 85 | 64 | 3.70E-13 |
| 0044281 | small molecule metabolic process | 2086 | 493 | 370 | 123 | 8.14E-13 |
| 0044036 | cell wall macromolecule metabolic process | 204 | 79 | 36 | 43 | 1.12E-12 |
| 0044283 | small molecule biosynthetic process | 754 | 211 | 134 | 77 | 1.32E-12 |
| 1903825 | organic acid transmembrane transport | 134 | 59 | 24 | 35 | 1.48E-12 |
| 1905039 | carboxylic acid transmembrane transport | 134 | 59 | 24 | 35 | 1.48E-12 |
| 0042546 | cell wall biogenesis | 133 | 58 | 24 | 34 | 3.71E-12 |
| 0044262 | cellular carbohydrate metabolic process | 525 | 157 | 93 | 64 | 4.30E-12 |
| 0006629 | lipid metabolic process | 1355 | 338 | 241 | 97 | 5.52E-12 |
| 0003333 | amino acid transmembrane transport | 122 | 54 | 22 | 32 | 1.00E-11 |
| 0032787 | monocarboxylic acid metabolic process | 571 | 165 | 101 | 64 | 2.56E-11 |
| 0016051 | carbohydrate biosynthetic process | 361 | 115 | 64 | 51 | 4.57E-11 |
| 0046942 | carboxylic acid transport | 184 | 70 | 33 | 37 | 5.50E-11 |
| 0015849 | organic acid transport | 184 | 70 | 33 | 37 | 5.50E-11 |
| 1902578 | single-organism localization | 1632 | 390 | 290 | 100 | 5.79E-11 |
| 0008610 | lipid biosynthetic process | 703 | 193 | 125 | 68 | 7.31E-11 |
| 0098656 | anion transmembrane transport | 240 | 84 | 43 | 41 | 1.07E-10 |
| 0006865 | amino acid transport | 130 | 54 | 23 | 31 | 1.91E-10 |
| 1901700 | response to oxygen-containing compound | 519 | 150 | 92 | 58 | 2.02E-10 |
| 0009834 | plant-type secondary cell wall biogenesis | 50 | 29 | 9 | 20 | 2.11E-10 |
| 0044765 | single-organism transport | 1561 | 372 | 277 | 95 | 2.40E-10 |
| 0034220 | ion transmembrane transport | 871 | 227 | 155 | 72 | 3.24E-10 |
| 0009832 | plant-type cell wall biogenesis | 91 | 42 | 16 | 26 | 3.92E-10 |
| 0042221 | response to chemical | 896 | 232 | 159 | 73 | 3.93E-10 |
| 0006820 | anion transport | 463 | 136 | 82 | 54 | 4.36E-10 |
| 0022900 | electron transport chain | 293 | 95 | 52 | 43 | 7.68E-10 |
| 0044264 | cellular polysaccharide metabolic process | 326 | 103 | 58 | 45 | 7.69E-10 |
| 0009698 | phenylpropanoid metabolic process | 96 | 43 | 17 | 26 | 7.88E-10 |
| 0015711 | organic anion transport | 314 | 100 | 56 | 44 | 8.36E-10 |
| 0071669 | plant-type cell wall organization or biogenesis | 94 | 42 | 17 | 25 | 1.36E-9 |
| 0009628 | response to abiotic stimulus | 885 | 227 | 157 | 70 | 1.50E-9 |
| 0006633 | fatty acid biosynthetic process | 170 | 63 | 30 | 33 | 1.69E-9 |
| 0010383 | cell wall polysaccharide metabolic process | 159 | 60 | 28 | 32 | 1.82E-9 |
| 0007017 | microtubule-based process | 269 | 88 | 48 | 40 | 1.95E-9 |
| 0051179 | localization | 3732 | 792 | 663 | 129 | 2.17E-9 |
| 0006082 | organic acid metabolic process | 1340 | 321 | 238 | 83 | 2.47E-9 |
| 0006631 | fatty acid metabolic process | 258 | 85 | 46 | 39 | 2.49E-9 |
| 0043436 | oxoacid metabolic process | 1337 | 320 | 237 | 83 | 2.89E-9 |
| 0018298 | protein-chromophore linkage | 35 | 22 | 6 | 16 | 3.92E-9 |
| 0019752 | carboxylic acid metabolic process | 1297 | 311 | 230 | 81 | 3.99E-9 |
| 0046394 | carboxylic acid biosynthetic process | 563 | 155 | 100 | 55 | 4.39E-9 |
| 0016053 | organic acid biosynthetic process | 563 | 155 | 100 | 55 | 4.39E-9 |
| 0006812 | cation transport | 752 | 196 | 134 | 62 | 5.27E-9 |
| 0044712 | single-organism catabolic process | 706 | 186 | 125 | 61 | 5.43E-9 |
| 0072330 | monocarboxylic acid biosynthetic process | 255 | 83 | 45 | 38 | 7.20E-9 |
| 0071705 | nitrogen compound transport | 530 | 146 | 94 | 52 | 1.18E-8 |
| 0034637 | cellular carbohydrate biosynthetic process | 266 | 85 | 47 | 38 | 1.26E-8 |
| 0010410 | hemicellulose metabolic process | 140 | 53 | 25 | 28 | 1.38E-8 |
| 0051234 | establishment of localization | 3597 | 759 | 639 | 120 | 1.46E-8 |
| 0016052 | carbohydrate catabolic process | 379 | 111 | 67 | 44 | 2.02E-8 |
| 0006810 | transport | 3568 | 748 | 634 | 114 | 5.90E-8 |
| 0044703 | multi-organism reproductive process | 39 | 22 | 7 | 15 | 6.48E-8 |
| 0001101 | response to acid chemical | 365 | 106 | 65 | 41 | 6.72E-8 |
| 0000271 | polysaccharide biosynthetic process | 238 | 76 | 42 | 34 | 7.46E-8 |
| 0006928 | movement of cell or subcellular component | 121 | 46 | 21 | 25 | 1.05E-7 |
| 0044042 | glucan metabolic process | 286 | 87 | 51 | 36 | 1.06E-7 |
| 0006073 | cellular glucan metabolic process | 274 | 84 | 49 | 35 | 1.20E-7 |
| 0044085 | cellular component biogenesis | 236 | 74 | 42 | 32 | 2.48E-7 |
| 0009651 | response to salt stress | 188 | 62 | 33 | 29 | 3.25E-7 |
| 0006790 | sulfur compound metabolic process | 367 | 104 | 65 | 39 | 3.32E-7 |
| 0045491 | xylan metabolic process | 71 | 31 | 13 | 18 | 3.56E-7 |
| 0015979 | photosynthesis | 97 | 38 | 17 | 21 | 5.39E-7 |
| 0044724 | single-organism carbohydrate catabolic process | 220 | 69 | 39 | 30 | 6.13E-7 |
| 0019953 | sexual reproduction | 37 | 20 | 7 | 13 | 6.56E-7 |
| 0008643 | carbohydrate transport | 204 | 65 | 36 | 29 | 6.89E-7 |
| 0044255 | cellular lipid metabolic process | 981 | 232 | 174 | 58 | 1.19E-6 |
| 0009808 | lignin metabolic process | 61 | 27 | 11 | 16 | 1.44E-6 |
| 0007018 | microtubule-based movement | 108 | 40 | 19 | 21 | 1.51E-6 |
| 0019748 | secondary metabolic process | 155 | 52 | 28 | 24 | 1.57E-6 |
| 0010033 | response to organic substance | 526 | 136 | 93 | 43 | 1.80E-6 |
| 0007010 | cytoskeleton organization | 176 | 57 | 31 | 26 | 1.82E-6 |
| 0009735 | response to cytokinin | 55 | 25 | 10 | 15 | 1.92E-6 |
| 0009734 | auxin-activated signaling pathway | 95 | 36 | 17 | 19 | 2.67E-6 |
| 0000003 | reproduction | 40 | 20 | 7 | 13 | 3.29E-6 |
| 1901606 | alpha-amino acid catabolic process | 85 | 33 | 15 | 18 | 3.71E-6 |
| 0006970 | response to osmotic stress | 214 | 65 | 38 | 27 | 4.31E-6 |
| 0033692 | cellular polysaccharide biosynthetic process | 210 | 64 | 37 | 27 | 4.49E-6 |
| 0015672 | monovalent inorganic cation transport | 456 | 119 | 81 | 38 | 4.82E-6 |
| 0016998 | cell wall macromolecule catabolic process | 32 | 17 | 6 | 11 | 6.24E-6 |
| 0042744 | hydrogen peroxide catabolic process | 154 | 50 | 27 | 23 | 7.03E-6 |
| 1902600 | hydrogen ion transmembrane transport | 350 | 95 | 62 | 33 | 7.70E-6 |
| 0015992 | proton transport | 350 | 95 | 62 | 33 | 7.70E-6 |
| 0006818 | hydrogen transport | 350 | 95 | 62 | 33 | 7.70E-6 |
| 0009409 | response to cold | 163 | 52 | 29 | 23 | 8.21E-6 |
| 0046034 | ATP metabolic process | 156 | 50 | 28 | 22 | 1.05E-5 |
| 0042743 | hydrogen peroxide metabolic process | 156 | 50 | 28 | 22 | 1.05E-5 |
| 0009812 | flavonoid metabolic process | 27 | 15 | 5 | 10 | 1.07E-5 |
| 0000278 | mitotic cell cycle | 30 | 16 | 5 | 11 | 1.10E-5 |
| 0006022 | aminoglycan metabolic process | 28 | 15 | 5 | 10 | 1.93E-5 |
| 0000226 | microtubule cytoskeleton organization | 147 | 47 | 26 | 21 | 2.04E-5 |
| 0030243 | cellulose metabolic process | 103 | 36 | 18 | 18 | 2.20E-5 |
| 0034219 | carbohydrate transmembrane transport | 131 | 43 | 23 | 20 | 2.21E-5 |
| 0006749 | glutathione metabolic process | 131 | 43 | 23 | 20 | 2.21E-5 |
| 0010215 | cellulose microfibril organization | 12 | 9 | 2 | 7 | 2.28E-5 |
| 0010087 | phloem or xylem histogenesis | 12 | 9 | 2 | 7 | 2.28E-5 |
| 0010089 | xylem development | 12 | 9 | 2 | 7 | 2.28E-5 |
| 0009699 | phenylpropanoid biosynthetic process | 55 | 23 | 10 | 13 | 2.64E-5 |
| 0009063 | cellular amino acid catabolic process | 96 | 34 | 17 | 17 | 2.68E-5 |
| 1901617 | organic hydroxy compound biosynthetic process | 195 | 58 | 35 | 23 | 2.72E-5 |
| 0005984 | disaccharide metabolic process | 100 | 35 | 18 | 17 | 2.75E-5 |
| 0044282 | small molecule catabolic process | 288 | 79 | 51 | 28 | 2.89E-5 |
| 0070592 | cell wall polysaccharide biosynthetic process | 52 | 22 | 9 | 13 | 3.17E-5 |
| 0009167 | purine ribonucleoside monophosphate metabolic process | 196 | 58 | 35 | 23 | 3.20E-5 |
| 0009126 | purine nucleoside monophosphate metabolic process | 196 | 58 | 35 | 23 | 3.20E-5 |
| 0046348 | amino sugar catabolic process | 26 | 14 | 5 | 9 | 3.40E-5 |
| 0006030 | chitin metabolic process | 26 | 14 | 5 | 9 | 3.40E-5 |
| 0006032 | chitin catabolic process | 26 | 14 | 5 | 9 | 3.40E-5 |
| 0006026 | aminoglycan catabolic process | 26 | 14 | 5 | 9 | 3.40E-5 |
| 1901072 | glucosamine-containing compound catabolic process | 26 | 14 | 5 | 9 | 3.40E-5 |
| 0009813 | flavonoid biosynthetic process | 26 | 14 | 5 | 9 | 3.40E-5 |
| 0098869 | cellular oxidant detoxification | 250 | 70 | 44 | 26 | 3.94E-5 |
| 0005996 | monosaccharide metabolic process | 233 | 66 | 41 | 25 | 4.37E-5 |
| 0009205 | purine ribonucleoside triphosphate metabolic process | 169 | 51 | 30 | 21 | 5.30E-5 |
| 0010035 | response to inorganic substance | 376 | 97 | 67 | 30 | 5.59E-5 |
| 0007049 | cell cycle | 61 | 24 | 11 | 13 | 5.88E-5 |
| 0019915 | lipid storage | 13 | 9 | 2 | 7 | 6.23E-5 |
| 0009773 | photosynthetic electron transport in photosystem I | 13 | 9 | 2 | 7 | 6.23E-5 |
| 0030244 | cellulose biosynthetic process | 69 | 26 | 12 | 14 | 7.00E-5 |
| 0009755 | hormone-mediated signaling pathway | 341 | 89 | 61 | 28 | 7.09E-5 |
| 0070589 | cellular component macromolecule biosynthetic process | 58 | 23 | 10 | 13 | 7.20E-5 |
| 0044038 | cell wall macromolecule biosynthetic process | 58 | 23 | 10 | 13 | 7.20E-5 |
| 0009414 | response to water deprivation | 137 | 43 | 24 | 19 | 7.30E-5 |
| 0051704 | multi-organism process | 473 | 117 | 84 | 33 | 7.41E-5 |
| 0009144 | purine nucleoside triphosphate metabolic process | 171 | 51 | 30 | 21 | 7.43E-5 |
| 0045493 | xylan catabolic process | 34 | 16 | 6 | 10 | 8.09E-5 |
| 0009266 | response to temperature stimulus | 329 | 86 | 58 | 28 | 8.76E-5 |
| 1990748 | cellular detoxification | 256 | 70 | 45 | 25 | 8.86E-5 |
| 0009064 | glutamine family amino acid metabolic process | 122 | 39 | 22 | 17 | 9.96E-5 |
| 0008202 | steroid metabolic process | 160 | 48 | 28 | 20 | 1.02E-4 |
| 0042886 | amide transport | 160 | 48 | 28 | 20 | 1.02E-4 |
| 0016125 | sterol metabolic process | 118 | 38 | 21 | 17 | 1.02E-4 |
| 0000272 | polysaccharide catabolic process | 186 | 54 | 33 | 21 | 1.03E-4 |
| 0009199 | ribonucleoside triphosphate metabolic process | 186 | 54 | 33 | 21 | 1.03E-4 |
| 0009415 | response to water | 139 | 43 | 25 | 18 | 1.06E-4 |
| 0007165 | signal transduction | 1357 | 293 | 241 | 52 | 1.16E-4 |
| 0009123 | nucleoside monophosphate metabolic process | 268 | 72 | 48 | 24 | 1.29E-4 |
| 1901071 | glucosamine-containing compound metabolic process | 32 | 15 | 6 | 9 | 1.43E-4 |
| 0098655 | cation transmembrane transport | 616 | 145 | 109 | 36 | 1.44E-4 |
| 1901615 | organic hydroxy compound metabolic process | 315 | 82 | 56 | 26 | 1.46E-4 |
| 0098754 | detoxification | 260 | 70 | 46 | 24 | 1.48E-4 |
| 0009056 | catabolic process | 1969 | 410 | 350 | 60 | 1.51E-4 |
| 0035672 | oligopeptide transmembrane transport | 116 | 37 | 21 | 16 | 1.56E-4 |
| 0006163 | purine nucleotide metabolic process | 270 | 72 | 48 | 24 | 1.65E-4 |
| 0006006 | glucose metabolic process | 80 | 28 | 14 | 14 | 1.68E-4 |
| 0015833 | peptide transport | 129 | 40 | 23 | 17 | 1.71E-4 |
| 0006857 | oligopeptide transport | 129 | 40 | 23 | 17 | 1.71E-4 |
| 0030198 | extracellular matrix organization | 26 | 13 | 5 | 8 | 1.76E-4 |
| 0043062 | extracellular structure organization | 26 | 13 | 5 | 8 | 1.76E-4 |
| 0043648 | dicarboxylic acid metabolic process | 121 | 38 | 21 | 17 | 1.83E-4 |
| 0072593 | reactive oxygen species metabolic process | 186 | 53 | 33 | 20 | 1.99E-4 |
| 0019318 | hexose metabolic process | 186 | 53 | 33 | 20 | 1.99E-4 |
| 0006694 | steroid biosynthetic process | 130 | 40 | 23 | 17 | 2.05E-4 |
| 0009719 | response to endogenous stimulus | 422 | 104 | 75 | 29 | 2.08E-4 |
| 0015718 | monocarboxylic acid transport | 33 | 15 | 6 | 9 | 2.19E-4 |
| 1901565 | organonitrogen compound catabolic process | 209 | 58 | 37 | 21 | 2.23E-4 |
| 0006040 | amino sugar metabolic process | 47 | 19 | 8 | 11 | 2.23E-4 |
| 0098660 | inorganic ion transmembrane transport | 622 | 145 | 110 | 35 | 2.29E-4 |
| 0019722 | calcium-mediated signaling | 30 | 14 | 5 | 9 | 2.51E-4 |
| 0042044 | fluid transport | 30 | 14 | 5 | 9 | 2.51E-4 |
| 0006833 | water transport | 30 | 14 | 5 | 9 | 2.51E-4 |
| 0009141 | nucleoside triphosphate metabolic process | 197 | 55 | 35 | 20 | 2.70E-4 |
| 0006979 | response to oxidative stress | 316 | 81 | 56 | 25 | 2.72E-4 |
| 0042542 | response to hydrogen peroxide | 37 | 16 | 7 | 9 | 2.76E-4 |
| 0006002 | fructose 6-phosphate metabolic process | 37 | 16 | 7 | 9 | 2.76E-4 |
| 0009725 | response to hormone | 392 | 97 | 70 | 27 | 2.87E-4 |
| 0009250 | glucan biosynthetic process | 158 | 46 | 28 | 18 | 2.99E-4 |
| 0019685 | photosynthesis, dark reaction | 15 | 9 | 3 | 6 | 3.09E-4 |
| 0019253 | reductive pentose-phosphate cycle | 15 | 9 | 3 | 6 | 3.09E-4 |
| 1901605 | alpha-amino acid metabolic process | 374 | 93 | 66 | 27 | 3.16E-4 |
| 0051273 | beta-glucan metabolic process | 124 | 38 | 22 | 16 | 3.19E-4 |
| 0006541 | glutamine metabolic process | 60 | 22 | 11 | 11 | 3.86E-4 |
| 0009416 | response to light stimulus | 282 | 73 | 50 | 23 | 3.89E-4 |
| 0014070 | response to organic cyclic compound | 76 | 26 | 13 | 13 | 4.25E-4 |
| 0016126 | sterol biosynthetic process | 57 | 21 | 10 | 11 | 4.83E-4 |
| 0051493 | regulation of cytoskeleton organization | 69 | 24 | 12 | 12 | 5.29E-4 |
| 0044092 | negative regulation of molecular function | 193 | 53 | 34 | 19 | 5.32E-4 |
| 0046292 | formaldehyde metabolic process | 13 | 8 | 2 | 6 | 5.39E-4 |
| 0046294 | formaldehyde catabolic process | 13 | 8 | 2 | 6 | 5.39E-4 |
| 0015813 | L-glutamate transport | 13 | 8 | 2 | 6 | 5.39E-4 |
| 0072521 | purine-containing compound metabolic process | 318 | 80 | 56 | 24 | 5.48E-4 |
| 0009937 | regulation of gibberellic acid mediated signaling pathway | 22 | 11 | 4 | 7 | 5.58E-4 |
| 0009161 | ribonucleoside monophosphate metabolic process | 230 | 61 | 41 | 20 | 5.77E-4 |
| 0046031 | ADP metabolic process | 119 | 36 | 21 | 15 | 5.91E-4 |
| 0006096 | glycolytic process | 119 | 36 | 21 | 15 | 5.91E-4 |
| 0006757 | ATP generation from ADP | 119 | 36 | 21 | 15 | 5.91E-4 |
| 0015802 | basic amino acid transport | 16 | 9 | 3 | 6 | 5.95E-4 |
| 0046395 | carboxylic acid catabolic process | 185 | 51 | 33 | 18 | 6.08E-4 |
| 0016054 | organic acid catabolic process | 185 | 51 | 33 | 18 | 6.08E-4 |
| 0015986 | ATP synthesis coupled proton transport | 29 | 13 | 5 | 8 | 6.75E-4 |
| 0015985 | energy coupled proton transport, down electrochemical gradient | 29 | 13 | 5 | 8 | 6.75E-4 |
| 0006754 | ATP biosynthetic process | 29 | 13 | 5 | 8 | 6.75E-4 |
| 0045492 | xylan biosynthetic process | 36 | 15 | 6 | 9 | 6.88E-4 |
| 0005991 | trehalose metabolic process | 36 | 15 | 6 | 9 | 6.88E-4 |
| 0098662 | inorganic cation transmembrane transport | 558 | 129 | 99 | 30 | 6.95E-4 |
| 0046128 | purine ribonucleoside metabolic process | 241 | 63 | 43 | 20 | 7.13E-4 |
| 0006165 | nucleoside diphosphate phosphorylation | 129 | 38 | 23 | 15 | 7.49E-4 |
| 0043086 | negative regulation of catalytic activity | 187 | 51 | 33 | 18 | 7.93E-4 |
| 0030001 | metal ion transport | 360 | 88 | 64 | 24 | 8.02E-4 |
| 0009179 | purine ribonucleoside diphosphate metabolic process | 121 | 36 | 21 | 15 | 8.32E-4 |
| 0009135 | purine nucleoside diphosphate metabolic process | 121 | 36 | 21 | 15 | 8.32E-4 |
| 0006733 | oxidoreduction coenzyme metabolic process | 215 | 57 | 38 | 19 | 8.64E-4 |
| 0009116 | nucleoside metabolic process | 318 | 79 | 56 | 23 | 8.77E-4 |
| 0016102 | diterpenoid biosynthetic process | 23 | 11 | 4 | 7 | 8.99E-4 |
| 0009185 | ribonucleoside diphosphate metabolic process | 126 | 37 | 22 | 15 | 9.29E-4 |
| 0010218 | response to far red light | 11 | 7 | 2 | 5 | 9.35E-4 |
| 0009314 | response to radiation | 300 | 75 | 53 | 22 | 9.65E-4 |
| 0051346 | negative regulation of hydrolase activity | 88 | 28 | 16 | 12 | 9.73E-4 |
| 0009623 | response to parasitic fungus | 4 | 4 | 1 | 3 | 9.93E-4 |
| 2000652 | regulation of secondary cell wall biogenesis | 4 | 4 | 1 | 3 | 9.93E-4 |
| 0015800 | acidic amino acid transport | 14 | 8 | 2 | 6 | 1.06E-3 |
| 0009150 | purine ribonucleotide metabolic process | 254 | 65 | 45 | 20 | 1.08E-3 |
| 0046148 | pigment biosynthetic process | 114 | 34 | 20 | 14 | 1.09E-3 |
| 0009132 | nucleoside diphosphate metabolic process | 136 | 39 | 24 | 15 | 1.13E-3 |
| 0042278 | purine nucleoside metabolic process | 250 | 64 | 44 | 20 | 1.16E-3 |
| 0030832 | regulation of actin filament length | 45 | 17 | 8 | 9 | 1.17E-3 |
| 0008064 | regulation of actin polymerization or depolymerization | 45 | 17 | 8 | 9 | 1.17E-3 |
| 0019932 | second-messenger-mediated signaling | 34 | 14 | 6 | 8 | 1.19E-3 |
| 0006012 | galactose metabolic process | 34 | 14 | 6 | 8 | 1.19E-3 |
| 0009620 | response to fungus | 132 | 38 | 23 | 15 | 1.20E-3 |
| 0046939 | nucleotide phosphorylation | 132 | 38 | 23 | 15 | 1.20E-3 |
| 0009058 | biosynthetic process | 3478 | 682 | 618 | 64 | 1.23E-3 |
| 0032970 | regulation of actin filament-based process | 49 | 18 | 9 | 9 | 1.24E-3 |
| 0010411 | xyloglucan metabolic process | 77 | 25 | 14 | 11 | 1.29E-3 |
| 0044550 | secondary metabolite biosynthetic process | 77 | 25 | 14 | 11 | 1.29E-3 |
| 0010951 | negative regulation of endopeptidase activity | 57 | 20 | 10 | 10 | 1.32E-3 |
| 0051274 | beta-glucan biosynthetic process | 90 | 28 | 16 | 12 | 1.43E-3 |
| 0005992 | trehalose biosynthetic process | 31 | 13 | 6 | 7 | 1.44E-3 |
| 0030148 | sphingolipid biosynthetic process | 42 | 16 | 7 | 9 | 1.46E-3 |
| 0033993 | response to lipid | 192 | 51 | 34 | 17 | 1.49E-3 |
| 0009119 | ribonucleoside metabolic process | 281 | 70 | 50 | 20 | 1.54E-3 |
| 0015976 | carbon utilization | 9 | 6 | 2 | 4 | 1.60E-3 |
| 0015804 | neutral amino acid transport | 9 | 6 | 2 | 4 | 1.60E-3 |
| 0052324 | plant-type cell wall cellulose biosynthetic process | 9 | 6 | 2 | 4 | 1.60E-3 |
| 0010037 | response to carbon dioxide | 9 | 6 | 2 | 4 | 1.60E-3 |
| 0052541 | plant-type cell wall cellulose metabolic process | 9 | 6 | 2 | 4 | 1.60E-3 |
| 0022904 | respiratory electron transport chain | 58 | 20 | 10 | 10 | 1.69E-3 |
| 0052548 | regulation of endopeptidase activity | 58 | 20 | 10 | 10 | 1.69E-3 |
| 0071702 | organic substance transport | 1692 | 346 | 300 | 46 | 1.73E-3 |
| 1901576 | organic substance biosynthetic process | 3220 | 632 | 572 | 60 | 1.74E-3 |
| 0061615 | glycolytic process through fructose-6-phosphate | 28 | 12 | 5 | 7 | 1.75E-3 |
| 0051494 | negative regulation of cytoskeleton organization | 18 | 9 | 3 | 6 | 1.80E-3 |
| 0031333 | negative regulation of protein complex assembly | 18 | 9 | 3 | 6 | 1.80E-3 |
| 0030837 | negative regulation of actin filament polymerization | 18 | 9 | 3 | 6 | 1.80E-3 |
| 0032272 | negative regulation of protein polymerization | 18 | 9 | 3 | 6 | 1.80E-3 |
| 1902475 | L-alpha-amino acid transmembrane transport | 18 | 9 | 3 | 6 | 1.80E-3 |
| 0030833 | regulation of actin filament polymerization | 39 | 15 | 7 | 8 | 1.82E-3 |
| 0046496 | nicotinamide nucleotide metabolic process | 180 | 48 | 32 | 16 | 1.85E-3 |
| 0043622 | cortical microtubule organization | 12 | 7 | 2 | 5 | 1.90E-3 |
| 0009686 | gibberellin biosynthetic process | 12 | 7 | 2 | 5 | 1.90E-3 |
| 0010417 | glucuronoxylan biosynthetic process | 12 | 7 | 2 | 5 | 1.90E-3 |
| 0010413 | glucuronoxylan metabolic process | 12 | 7 | 2 | 5 | 1.90E-3 |
| 0006090 | pyruvate metabolic process | 144 | 40 | 26 | 14 | 1.90E-3 |
| 1902022 | L-lysine transport | 15 | 8 | 3 | 5 | 1.92E-3 |
| 0015819 | lysine transport | 15 | 8 | 3 | 5 | 1.92E-3 |
| 1903401 | L-lysine transmembrane transport | 15 | 8 | 3 | 5 | 1.92E-3 |
| 0015706 | nitrate transport | 15 | 8 | 3 | 5 | 1.92E-3 |
| 0016101 | diterpenoid metabolic process | 43 | 16 | 8 | 8 | 1.95E-3 |
| 0046271 | phenylpropanoid catabolic process | 32 | 13 | 6 | 7 | 2.04E-3 |
| 0046274 | lignin catabolic process | 32 | 13 | 6 | 7 | 2.04E-3 |
| 0009809 | lignin biosynthetic process | 25 | 11 | 4 | 7 | 2.09E-3 |
| 0046351 | disaccharide biosynthetic process | 51 | 18 | 9 | 9 | 2.10E-3 |
| 1901657 | glycosyl compound metabolic process | 342 | 82 | 61 | 21 | 2.12E-3 |
| 0009206 | purine ribonucleoside triphosphate biosynthetic process | 36 | 14 | 6 | 8 | 2.27E-3 |
| 0009145 | purine nucleoside triphosphate biosynthetic process | 36 | 14 | 6 | 8 | 2.27E-3 |
| 0019362 | pyridine nucleotide metabolic process | 182 | 48 | 32 | 16 | 2.36E-3 |
| 0032271 | regulation of protein polymerization | 40 | 15 | 7 | 8 | 2.45E-3 |
| 0030029 | actin filament-based process | 22 | 10 | 4 | 6 | 2.48E-3 |
| 0009767 | photosynthetic electron transport chain | 29 | 12 | 5 | 7 | 2.50E-3 |
| 0009637 | response to blue light | 44 | 16 | 8 | 8 | 2.57E-3 |
| 0009833 | plant-type primary cell wall biogenesis | 44 | 16 | 8 | 8 | 2.57E-3 |
| 0072350 | tricarboxylic acid metabolic process | 68 | 22 | 12 | 10 | 2.57E-3 |
| 0032956 | regulation of actin cytoskeleton organization | 48 | 17 | 9 | 8 | 2.64E-3 |
| 0051235 | maintenance of location | 52 | 18 | 9 | 9 | 2.68E-3 |
| 0006107 | oxaloacetate metabolic process | 7 | 5 | 1 | 4 | 2.69E-3 |
| 0015808 | L-alanine transport | 7 | 5 | 1 | 4 | 2.69E-3 |
| 0015812 | gamma-aminobutyric acid transport | 7 | 5 | 1 | 4 | 2.69E-3 |
| 0032328 | alanine transport | 7 | 5 | 1 | 4 | 2.69E-3 |
| 0009072 | aromatic amino acid family metabolic process | 98 | 29 | 17 | 12 | 2.77E-3 |
| 0016114 | terpenoid biosynthetic process | 98 | 29 | 17 | 12 | 2.77E-3 |
| 1901135 | carbohydrate derivative metabolic process | 695 | 152 | 123 | 29 | 2.86E-3 |
| 1902589 | single-organism organelle organization | 298 | 72 | 53 | 19 | 3.09E-3 |
| 0072524 | pyridine-containing compound metabolic process | 189 | 49 | 34 | 15 | 3.11E-3 |
| 0035821 | modification of morphology or physiology of other organism | 16 | 8 | 3 | 5 | 3.25E-3 |
| 0009938 | negative regulation of gibberellic acid mediated signaling pathway | 16 | 8 | 3 | 5 | 3.25E-3 |
| 0043467 | regulation of generation of precursor metabolites and energy | 16 | 8 | 3 | 5 | 3.25E-3 |
| 0006869 | lipid transport | 171 | 45 | 30 | 15 | 3.30E-3 |
| 0006101 | citrate metabolic process | 61 | 20 | 11 | 9 | 3.32E-3 |
| 0006099 | tricarboxylic acid cycle | 61 | 20 | 11 | 9 | 3.32E-3 |
| 0033356 | UDP-L-arabinose metabolic process | 10 | 6 | 2 | 4 | 3.41E-3 |
| 0006122 | mitochondrial electron transport, ubiquinol to cytochrome c | 13 | 7 | 2 | 5 | 3.49E-3 |
| 0019388 | galactose catabolic process | 30 | 12 | 5 | 7 | 3.50E-3 |
| 0007015 | actin filament organization | 104 | 30 | 18 | 12 | 3.60E-3 |
| 0009259 | ribonucleotide metabolic process | 295 | 71 | 52 | 19 | 3.63E-3 |
| 0009065 | glutamine family amino acid catabolic process | 23 | 10 | 4 | 6 | 3.70E-3 |
| 0006094 | gluconeogenesis | 34 | 13 | 6 | 7 | 3.84E-3 |
| 0010466 | negative regulation of peptidase activity | 62 | 20 | 11 | 9 | 4.10E-3 |
| 0005985 | sucrose metabolic process | 58 | 19 | 10 | 9 | 4.19E-3 |
| 0050826 | response to freezing | 5 | 4 | 1 | 3 | 4.26E-3 |
| 0051553 | flavone biosynthetic process | 5 | 4 | 1 | 3 | 4.26E-3 |
| 0051552 | flavone metabolic process | 5 | 4 | 1 | 3 | 4.26E-3 |
| 0007264 | small GTPase mediated signal transduction | 75 | 23 | 13 | 10 | 4.45E-3 |
| 0019693 | ribose phosphate metabolic process | 332 | 78 | 59 | 19 | 4.65E-3 |
| 0009168 | purine ribonucleoside monophosphate biosynthetic process | 67 | 21 | 12 | 9 | 4.84E-3 |
| 0009127 | purine nucleoside monophosphate biosynthetic process | 67 | 21 | 12 | 9 | 4.84E-3 |
| 0030154 | cell differentiation | 165 | 43 | 29 | 14 | 4.84E-3 |
| 0045861 | negative regulation of proteolysis | 63 | 20 | 11 | 9 | 5.02E-3 |
| 0019319 | hexose biosynthetic process | 35 | 13 | 6 | 7 | 5.13E-3 |
| 0010315 | auxin efflux | 17 | 8 | 3 | 5 | 5.19E-3 |
| 0042549 | photosystem II stabilization | 3 | 3 | 1 | 2 | 5.60E-3 |
| 0015708 | silicate transport | 3 | 3 | 1 | 2 | 5.60E-3 |
| 0051208 | sequestering of calcium ion | 3 | 3 | 1 | 2 | 5.60E-3 |
| 0006721 | terpenoid metabolic process | 134 | 36 | 24 | 12 | 5.61E-3 |
| 0006575 | cellular modified amino acid metabolic process | 204 | 51 | 36 | 15 | 5.70E-3 |
| 0009117 | nucleotide metabolic process | 434 | 98 | 77 | 21 | 5.81E-3 |
| 0009646 | response to absence of light | 14 | 7 | 2 | 5 | 5.91E-3 |
| 0030865 | cortical cytoskeleton organization | 14 | 7 | 2 | 5 | 5.91E-3 |
| 0009311 | oligosaccharide metabolic process | 139 | 37 | 25 | 12 | 5.92E-3 |
| 0002252 | immune effector process | 8 | 5 | 1 | 4 | 6.12E-3 |
| 0009608 | response to symbiont | 8 | 5 | 1 | 4 | 6.12E-3 |
| 0009610 | response to symbiotic fungus | 8 | 5 | 1 | 4 | 6.12E-3 |
| 0043903 | regulation of symbiosis, encompassing mutualism through parasitism | 8 | 5 | 1 | 4 | 6.12E-3 |
| 0015980 | energy derivation by oxidation of organic compounds | 60 | 19 | 11 | 8 | 6.34E-3 |
| 0009611 | response to wounding | 77 | 23 | 14 | 9 | 6.35E-3 |
| 0044273 | sulfur compound catabolic process | 11 | 6 | 2 | 4 | 6.37E-3 |
| 0009071 | serine family amino acid catabolic process | 11 | 6 | 2 | 4 | 6.37E-3 |
| 0009685 | gibberellin metabolic process | 32 | 12 | 6 | 6 | 6.47E-3 |
| 0015807 | L-amino acid transport | 21 | 9 | 4 | 5 | 6.53E-3 |
| 0045489 | pectin biosynthetic process | 36 | 13 | 6 | 7 | 6.75E-3 |
| 0046364 | monosaccharide biosynthetic process | 44 | 15 | 8 | 7 | 6.91E-3 |
| 0046365 | monosaccharide catabolic process | 44 | 15 | 8 | 7 | 6.91E-3 |
| 0042440 | pigment metabolic process | 136 | 36 | 24 | 12 | 7.21E-3 |
| 0052547 | regulation of peptidase activity | 65 | 20 | 12 | 8 | 7.37E-3 |
| 0006528 | asparagine metabolic process | 18 | 8 | 3 | 5 | 7.91E-3 |
| 0043455 | regulation of secondary metabolic process | 18 | 8 | 3 | 5 | 7.91E-3 |
| 0097305 | response to alcohol | 160 | 41 | 28 | 13 | 7.92E-3 |
| 0009142 | nucleoside triphosphate biosynthetic process | 57 | 18 | 10 | 8 | 8.01E-3 |
| 0006558 | L-phenylalanine metabolic process | 29 | 11 | 5 | 6 | 8.15E-3 |
| 0046940 | nucleoside monophosphate phosphorylation | 29 | 11 | 5 | 6 | 8.15E-3 |
| 1902221 | erythrose 4-phosphate/phosphoenolpyruvate family amino acid metabolic process | 29 | 11 | 5 | 6 | 8.15E-3 |
| 0009201 | ribonucleoside triphosphate biosynthetic process | 53 | 17 | 9 | 8 | 8.29E-3 |
| 0061572 | actin filament bundle organization | 33 | 12 | 6 | 6 | 8.55E-3 |
| 0051017 | actin filament bundle assembly | 33 | 12 | 6 | 6 | 8.55E-3 |
| 0007167 | enzyme linked receptor protein signaling pathway | 276 | 65 | 49 | 16 | 8.62E-3 |
| 0007178 | transmembrane receptor protein serine/threonine kinase signaling pathway | 276 | 65 | 49 | 16 | 8.62E-3 |
| 0009607 | response to biotic stimulus | 415 | 93 | 74 | 19 | 8.71E-3 |
| 0009753 | response to jasmonic acid | 37 | 13 | 7 | 6 | 8.74E-3 |
| 1901575 | organic substance catabolic process | 1813 | 360 | 322 | 38 | 8.86E-3 |
| 1901564 | organonitrogen compound metabolic process | 2034 | 401 | 361 | 40 | 9.22E-3 |
| 0043207 | response to external biotic stimulus | 391 | 88 | 69 | 19 | 9.35E-3 |
| 0021700 | developmental maturation | 15 | 7 | 3 | 4 | 9.40E-3 |
| 0010648 | negative regulation of cell communication | 58 | 18 | 10 | 8 | 9.73E-3 |
| 0023057 | negative regulation of signaling | 58 | 18 | 10 | 8 | 9.73E-3 |
| 0009968 | negative regulation of signal transduction | 58 | 18 | 10 | 8 | 9.73E-3 |
| 0009074 | aromatic amino acid family catabolic process | 26 | 10 | 5 | 5 | 0.01 |
| 0006753 | nucleoside phosphate metabolic process | 443 | 98 | 79 | 19 | 0.01 |
| 0009225 | nucleotide-sugar metabolic process | 50 | 16 | 9 | 7 | 0.01 |
| 0044247 | cellular polysaccharide catabolic process | 67 | 20 | 12 | 8 | 0.01 |
| 0009737 | response to abscisic acid | 158 | 40 | 28 | 12 | 0.01 |
| 0009743 | response to carbohydrate | 46 | 15 | 8 | 7 | 0.01 |
| 0015995 | chlorophyll biosynthetic process | 30 | 11 | 5 | 6 | 0.01 |
| 0042362 | fat-soluble vitamin biosynthetic process | 12 | 6 | 2 | 4 | 0.01 |
| 0006552 | leucine catabolic process | 12 | 6 | 2 | 4 | 0.01 |
| 0030036 | actin cytoskeleton organization | 12 | 6 | 2 | 4 | 0.01 |
| 0048870 | cell motility | 12 | 6 | 2 | 4 | 0.01 |
| 0030522 | intracellular receptor signaling pathway | 12 | 6 | 2 | 4 | 0.01 |
| 0006775 | fat-soluble vitamin metabolic process | 12 | 6 | 2 | 4 | 0.01 |
| 0009785 | blue light signaling pathway | 12 | 6 | 2 | 4 | 0.01 |
| 0051238 | sequestering of metal ion | 12 | 6 | 2 | 4 | 0.01 |
| 0031532 | actin cytoskeleton reorganization | 6 | 4 | 1 | 3 | 0.01 |
| 0008295 | spermidine biosynthetic process | 6 | 4 | 1 | 3 | 0.01 |
| 0006536 | glutamate metabolic process | 34 | 12 | 6 | 6 | 0.01 |
| 0006534 | cysteine metabolic process | 38 | 13 | 7 | 6 | 0.01 |
| 1903826 | arginine transmembrane transport | 9 | 5 | 2 | 3 | 0.01 |
| 0042752 | regulation of circadian rhythm | 9 | 5 | 2 | 3 | 0.01 |
| 0010166 | wax metabolic process | 9 | 5 | 2 | 3 | 0.01 |
| 0015809 | arginine transport | 9 | 5 | 2 | 3 | 0.01 |
| 0010025 | wax biosynthetic process | 9 | 5 | 2 | 3 | 0.01 |
| 0009854 | oxidative photosynthetic carbon pathway | 9 | 5 | 2 | 3 | 0.01 |
| 0055086 | nucleobase-containing small molecule metabolic process | 511 | 111 | 91 | 20 | 0.01 |
| 0044272 | sulfur compound biosynthetic process | 145 | 37 | 26 | 11 | 0.01 |
| 0008299 | isoprenoid biosynthetic process | 141 | 36 | 25 | 11 | 0.01 |
| 0006108 | malate metabolic process | 23 | 9 | 4 | 5 | 0.01 |
| 0051707 | response to other organism | 381 | 85 | 68 | 17 | 0.01 |
| 0015781 | pyrimidine nucleotide-sugar transport | 47 | 15 | 8 | 7 | 0.01 |
| 0031407 | oxylipin metabolic process | 27 | 10 | 5 | 5 | 0.01 |
| 0031408 | oxylipin biosynthetic process | 27 | 10 | 5 | 5 | 0.01 |
| 0006164 | purine nucleotide biosynthetic process | 100 | 27 | 18 | 9 | 0.01 |
| 0000096 | sulfur amino acid metabolic process | 60 | 18 | 11 | 7 | 0.01 |
| 0019320 | hexose catabolic process | 39 | 13 | 7 | 6 | 0.01 |
| 0006835 | dicarboxylic acid transport | 31 | 11 | 6 | 5 | 0.01 |
| 0006551 | leucine metabolic process | 16 | 7 | 3 | 4 | 0.01 |
| 0001676 | long-chain fatty acid metabolic process | 16 | 7 | 3 | 4 | 0.01 |
| 0019740 | nitrogen utilization | 16 | 7 | 3 | 4 | 0.01 |
| 0031221 | arabinan metabolic process | 16 | 7 | 3 | 4 | 0.01 |
| 0031222 | arabinan catabolic process | 16 | 7 | 3 | 4 | 0.01 |
| 0015791 | polyol transport | 16 | 7 | 3 | 4 | 0.01 |
| 0046513 | ceramide biosynthetic process | 16 | 7 | 3 | 4 | 0.01 |
| 0006814 | sodium ion transport | 35 | 12 | 6 | 6 | 0.01 |
| 0042430 | indole-containing compound metabolic process | 52 | 16 | 9 | 7 | 0.02 |
| 0010243 | response to organonitrogen compound | 52 | 16 | 9 | 7 | 0.02 |
| 0042451 | purine nucleoside biosynthetic process | 74 | 21 | 13 | 8 | 0.02 |
| 0046129 | purine ribonucleoside biosynthetic process | 74 | 21 | 13 | 8 | 0.02 |
| 0006559 | L-phenylalanine catabolic process | 20 | 8 | 4 | 4 | 0.02 |
| 0005978 | glycogen biosynthetic process | 20 | 8 | 4 | 4 | 0.02 |
| 0030834 | regulation of actin filament depolymerization | 20 | 8 | 4 | 4 | 0.02 |
| 1902222 | erythrose 4-phosphate/phosphoenolpyruvate family amino acid catabolic process | 20 | 8 | 4 | 4 | 0.02 |
| 1901879 | regulation of protein depolymerization | 20 | 8 | 4 | 4 | 0.02 |
| 0072522 | purine-containing compound biosynthetic process | 120 | 31 | 21 | 10 | 0.02 |
| 0072334 | UDP-galactose transmembrane transport | 13 | 6 | 2 | 4 | 0.02 |
| 0030042 | actin filament depolymerization | 13 | 6 | 2 | 4 | 0.02 |
| 0010431 | seed maturation | 13 | 6 | 2 | 4 | 0.02 |
| 0071483 | cellular response to blue light | 13 | 6 | 2 | 4 | 0.02 |
| 0015785 | UDP-galactose transport | 13 | 6 | 2 | 4 | 0.02 |
| 0035337 | fatty-acyl-CoA metabolic process | 13 | 6 | 2 | 4 | 0.02 |
| 0007266 | Rho protein signal transduction | 13 | 6 | 2 | 4 | 0.02 |
| 0046417 | chorismate metabolic process | 24 | 9 | 4 | 5 | 0.02 |
| 0010109 | regulation of photosynthesis | 36 | 12 | 6 | 6 | 0.02 |
| 0019252 | starch biosynthetic process | 28 | 10 | 5 | 5 | 0.02 |
| 0009313 | oligosaccharide catabolic process | 32 | 11 | 6 | 5 | 0.02 |
| 0046352 | disaccharide catabolic process | 32 | 11 | 6 | 5 | 0.02 |
| 0010928 | regulation of auxin mediated signaling pathway | 32 | 11 | 6 | 5 | 0.02 |
| 0048869 | cellular developmental process | 311 | 70 | 55 | 15 | 0.02 |
| 0009069 | serine family amino acid metabolic process | 75 | 21 | 13 | 8 | 0.02 |
| 0042335 | cuticle development | 4 | 3 | 1 | 2 | 0.02 |
| 0007097 | nuclear migration | 4 | 3 | 1 | 2 | 0.02 |
| 0040023 | establishment of nucleus localization | 4 | 3 | 1 | 2 | 0.02 |
| 0051647 | nucleus localization | 4 | 3 | 1 | 2 | 0.02 |
| 0042776 | mitochondrial ATP synthesis coupled proton transport | 4 | 3 | 1 | 2 | 0.02 |
| 0010236 | plastoquinone biosynthetic process | 4 | 3 | 1 | 2 | 0.02 |
| 0032469 | endoplasmic reticulum calcium ion homeostasis | 4 | 3 | 1 | 2 | 0.02 |
| 1902930 | regulation of alcohol biosynthetic process | 4 | 3 | 1 | 2 | 0.02 |
| 0010115 | regulation of abscisic acid biosynthetic process | 4 | 3 | 1 | 2 | 0.02 |
| 0042989 | sequestering of actin monomers | 4 | 3 | 1 | 2 | 0.02 |
| 0009726 | detection of endogenous stimulus | 4 | 3 | 1 | 2 | 0.02 |
| 0009729 | detection of brassinosteroid stimulus | 4 | 3 | 1 | 2 | 0.02 |
| 0009720 | detection of hormone stimulus | 4 | 3 | 1 | 2 | 0.02 |
| 0006637 | acyl-CoA metabolic process | 49 | 15 | 9 | 6 | 0.02 |
| 0035383 | thioester metabolic process | 49 | 15 | 9 | 6 | 0.02 |
| 0042360 | vitamin E metabolic process | 10 | 5 | 2 | 3 | 0.02 |
| 0019755 | one-carbon compound transport | 10 | 5 | 2 | 3 | 0.02 |
| 0046283 | anthocyanin-containing compound metabolic process | 10 | 5 | 2 | 3 | 0.02 |
| 0006268 | DNA unwinding involved in DNA replication | 10 | 5 | 2 | 3 | 0.02 |
| 0010189 | vitamin E biosynthetic process | 10 | 5 | 2 | 3 | 0.02 |
| 0015840 | urea transport | 10 | 5 | 2 | 3 | 0.02 |
| 0070413 | trehalose metabolism in response to stress | 17 | 7 | 3 | 4 | 0.02 |
| 0008154 | actin polymerization or depolymerization | 17 | 7 | 3 | 4 | 0.02 |
| 0045944 | positive regulation of transcription from RNA polymerase II promoter | 258 | 59 | 46 | 13 | 0.02 |
| 0008216 | spermidine metabolic process | 7 | 4 | 1 | 3 | 0.02 |
| 1903338 | regulation of cell wall organization or biogenesis | 7 | 4 | 1 | 3 | 0.02 |
| 0051607 | defense response to virus | 7 | 4 | 1 | 3 | 0.02 |
| 0009118 | regulation of nucleoside metabolic process | 7 | 4 | 1 | 3 | 0.02 |
| 0006638 | neutral lipid metabolic process | 37 | 12 | 7 | 5 | 0.02 |
| 0006639 | acylglycerol metabolic process | 37 | 12 | 7 | 5 | 0.02 |
| 0009060 | aerobic respiration | 25 | 9 | 4 | 5 | 0.02 |
| 0009605 | response to external stimulus | 565 | 119 | 100 | 19 | 0.02 |
| 0009850 | auxin metabolic process | 29 | 10 | 5 | 5 | 0.02 |
| 0045333 | cellular respiration | 29 | 10 | 5 | 5 | 0.02 |
| 0008652 | cellular amino acid biosynthetic process | 275 | 62 | 49 | 13 | 0.02 |
| 0042401 | cellular biogenic amine biosynthetic process | 46 | 14 | 8 | 6 | 0.03 |
| 0009309 | amine biosynthetic process | 46 | 14 | 8 | 6 | 0.03 |
| 0030835 | negative regulation of actin filament depolymerization | 14 | 6 | 2 | 4 | 0.03 |
| 0051693 | actin filament capping | 14 | 6 | 2 | 4 | 0.03 |
| 0051817 | modification of morphology or physiology of other organism involved in symbiotic interaction | 14 | 6 | 2 | 4 | 0.03 |
| 0044003 | modification by symbiont of host morphology or physiology | 14 | 6 | 2 | 4 | 0.03 |
| 1901880 | negative regulation of protein depolymerization | 14 | 6 | 2 | 4 | 0.03 |
| 0045488 | pectin metabolic process | 91 | 24 | 16 | 8 | 0.03 |
| 0010393 | galacturonan metabolic process | 91 | 24 | 16 | 8 | 0.03 |
| 0009152 | purine ribonucleotide biosynthetic process | 91 | 24 | 16 | 8 | 0.03 |
| 0048609 | multicellular organismal reproductive process | 42 | 13 | 7 | 6 | 0.03 |
| 0009751 | response to salicylic acid | 42 | 13 | 7 | 6 | 0.03 |
| 0005982 | starch metabolic process | 55 | 16 | 10 | 6 | 0.03 |
| 0015698 | inorganic anion transport | 148 | 36 | 26 | 10 | 0.03 |
| 1901136 | carbohydrate derivative catabolic process | 73 | 20 | 13 | 7 | 0.03 |
| 0006720 | isoprenoid metabolic process | 187 | 44 | 33 | 11 | 0.03 |
| 0015850 | organic hydroxy compound transport | 38 | 12 | 7 | 5 | 0.03 |
| 0010119 | regulation of stomatal movement | 38 | 12 | 7 | 5 | 0.03 |
| 0065009 | regulation of molecular function | 647 | 134 | 115 | 19 | 0.03 |
| 0006732 | coenzyme metabolic process | 414 | 89 | 74 | 15 | 0.03 |
| 0009423 | chorismate biosynthetic process | 18 | 7 | 3 | 4 | 0.03 |
| 0046460 | neutral lipid biosynthetic process | 18 | 7 | 3 | 4 | 0.03 |
| 0046463 | acylglycerol biosynthetic process | 18 | 7 | 3 | 4 | 0.03 |
| 0070887 | cellular response to chemical stimulus | 173 | 41 | 31 | 10 | 0.03 |
| 0009642 | response to light intensity | 60 | 17 | 11 | 6 | 0.03 |
| 0007265 | Ras protein signal transduction | 69 | 19 | 12 | 7 | 0.03 |
| 1901420 | negative regulation of response to alcohol | 22 | 8 | 4 | 4 | 0.03 |
| 0034284 | response to monosaccharide | 22 | 8 | 4 | 4 | 0.03 |
| 0009788 | negative regulation of abscisic acid-activated signaling pathway | 22 | 8 | 4 | 4 | 0.03 |
| 0009746 | response to hexose | 22 | 8 | 4 | 4 | 0.03 |
| 0033499 | galactose catabolic process via UDP-galactose | 26 | 9 | 5 | 4 | 0.03 |
| 0050832 | defense response to fungus | 111 | 28 | 20 | 8 | 0.03 |
| 0006766 | vitamin metabolic process | 97 | 25 | 17 | 8 | 0.03 |
| 0009073 | aromatic amino acid family biosynthetic process | 65 | 18 | 12 | 6 | 0.03 |
| 0044126 | regulation of growth of symbiont in host | 2 | 2 | 0 | 2 | 0.03 |
| 0044130 | negative regulation of growth of symbiont in host | 2 | 2 | 0 | 2 | 0.03 |
| 0044144 | modulation of growth of symbiont involved in interaction with host | 2 | 2 | 0 | 2 | 0.03 |
| 0044146 | negative regulation of growth of symbiont involved in interaction with host | 2 | 2 | 0 | 2 | 0.03 |
| 0009649 | entrainment of circadian clock | 2 | 2 | 0 | 2 | 0.03 |
| 0048657 | anther wall tapetum cell differentiation | 2 | 2 | 0 | 2 | 0.03 |
| 0034551 | mitochondrial respiratory chain complex III assembly | 2 | 2 | 0 | 2 | 0.03 |
| 0010540 | basipetal auxin transport | 2 | 2 | 0 | 2 | 0.03 |
| 0044364 | disruption of cells of other organism | 2 | 2 | 0 | 2 | 0.03 |
| 0080026 | response to indolebutyric acid | 2 | 2 | 0 | 2 | 0.03 |
| 0080024 | indolebutyric acid metabolic process | 2 | 2 | 0 | 2 | 0.03 |
| 1902850 | microtubule cytoskeleton organization involved in mitosis | 2 | 2 | 0 | 2 | 0.03 |
| 0001505 | regulation of neurotransmitter levels | 2 | 2 | 0 | 2 | 0.03 |
| 0017062 | respiratory chain complex III assembly | 2 | 2 | 0 | 2 | 0.03 |
| 0031341 | regulation of cell killing | 2 | 2 | 0 | 2 | 0.03 |
| 0052865 | 1-deoxy-D-xylulose 5-phosphate biosynthetic process | 2 | 2 | 0 | 2 | 0.03 |
| 0031343 | positive regulation of cell killing | 2 | 2 | 0 | 2 | 0.03 |
| 0052863 | 1-deoxy-D-xylulose 5-phosphate metabolic process | 2 | 2 | 0 | 2 | 0.03 |
| 1905775 | --- | 2 | 2 | 0 | 2 | 0.03 |
| 0030845 | phospholipase C-inhibiting G-protein coupled receptor signaling pathway | 2 | 2 | 0 | 2 | 0.03 |
| 0090307 | mitotic spindle assembly | 2 | 2 | 0 | 2 | 0.03 |
| 0090357 | regulation of tryptophan metabolic process | 2 | 2 | 0 | 2 | 0.03 |
| 0080153 | negative regulation of reductive pentose-phosphate cycle | 2 | 2 | 0 | 2 | 0.03 |
| 0080152 | regulation of reductive pentose-phosphate cycle | 2 | 2 | 0 | 2 | 0.03 |
| 0036066 | protein O-linked fucosylation | 2 | 2 | 0 | 2 | 0.03 |
| 0000098 | sulfur amino acid catabolic process | 2 | 2 | 0 | 2 | 0.03 |
| 0090410 | malonate catabolic process | 2 | 2 | 0 | 2 | 0.03 |
| 0097033 | mitochondrial respiratory chain complex III biogenesis | 2 | 2 | 0 | 2 | 0.03 |
| 0051712 | positive regulation of killing of cells of other organism | 2 | 2 | 0 | 2 | 0.03 |
| 0051714 | positive regulation of cytolysis in other organism | 2 | 2 | 0 | 2 | 0.03 |
| 0051710 | regulation of cytolysis in other organism | 2 | 2 | 0 | 2 | 0.03 |
| 0051709 | regulation of killing of cells of other organism | 2 | 2 | 0 | 2 | 0.03 |
| 2000603 | regulation of secondary growth | 2 | 2 | 0 | 2 | 0.03 |
| 2000605 | positive regulation of secondary growth | 2 | 2 | 0 | 2 | 0.03 |
| 0019447 | D-cysteine catabolic process | 2 | 2 | 0 | 2 | 0.03 |
| 0045919 | positive regulation of cytolysis | 2 | 2 | 0 | 2 | 0.03 |
| 1902025 | nitrate import | 2 | 2 | 0 | 2 | 0.03 |
| 0090548 | response to nitrate starvation | 2 | 2 | 0 | 2 | 0.03 |
| 0046438 | D-cysteine metabolic process | 2 | 2 | 0 | 2 | 0.03 |
| 0051802 | regulation of cytolysis in other organism involved in symbiotic interaction | 2 | 2 | 0 | 2 | 0.03 |
| 0010148 | transpiration | 2 | 2 | 0 | 2 | 0.03 |
| 0051804 | positive regulation of cytolysis in other organism involved in symbiotic interaction | 2 | 2 | 0 | 2 | 0.03 |
| 0010110 | regulation of photosynthesis, dark reaction | 2 | 2 | 0 | 2 | 0.03 |
| 0051841 | positive regulation by host of cytolysis of symbiont cells | 2 | 2 | 0 | 2 | 0.03 |
| 0051839 | regulation by host of cytolysis of symbiont cells | 2 | 2 | 0 | 2 | 0.03 |
| 1900367 | positive regulation of defense response to insect | 2 | 2 | 0 | 2 | 0.03 |
| 0006882 | cellular zinc ion homeostasis | 2 | 2 | 0 | 2 | 0.03 |
| 0009093 | cysteine catabolic process | 2 | 2 | 0 | 2 | 0.03 |
| 0050691 | regulation of defense response to virus by host | 2 | 2 | 0 | 2 | 0.03 |
| 0001906 | cell killing | 2 | 2 | 0 | 2 | 0.03 |
| 0042268 | regulation of cytolysis | 2 | 2 | 0 | 2 | 0.03 |
| 0031640 | killing of cells of other organism | 2 | 2 | 0 | 2 | 0.03 |
| 0060236 | regulation of mitotic spindle organization | 11 | 5 | 2 | 3 | 0.03 |
| 0006529 | asparagine biosynthetic process | 11 | 5 | 2 | 3 | 0.03 |
| 0042548 | regulation of photosynthesis, light reaction | 11 | 5 | 2 | 3 | 0.03 |
| 0090224 | regulation of spindle organization | 11 | 5 | 2 | 3 | 0.03 |
| 0016477 | cell migration | 11 | 5 | 2 | 3 | 0.03 |
| 0006140 | regulation of nucleotide metabolic process | 11 | 5 | 2 | 3 | 0.03 |
| 0010093 | specification of floral organ identity | 11 | 5 | 2 | 3 | 0.03 |
| 0035336 | long-chain fatty-acyl-CoA metabolic process | 11 | 5 | 2 | 3 | 0.03 |
| 0009803 | cinnamic acid metabolic process | 11 | 5 | 2 | 3 | 0.03 |
| 0090701 | specification of plant organ identity | 11 | 5 | 2 | 3 | 0.03 |
| 0009800 | cinnamic acid biosynthetic process | 11 | 5 | 2 | 3 | 0.03 |
| 1900542 | regulation of purine nucleotide metabolic process | 11 | 5 | 2 | 3 | 0.03 |
| 0010817 | regulation of hormone levels | 184 | 43 | 33 | 10 | 0.03 |
| 0043650 | dicarboxylic acid biosynthetic process | 43 | 13 | 8 | 5 | 0.03 |
| 0044275 | cellular carbohydrate catabolic process | 107 | 27 | 19 | 8 | 0.03 |
| 0000302 | response to reactive oxygen species | 75 | 20 | 13 | 7 | 0.04 |
| 0042127 | regulation of cell proliferation | 66 | 18 | 12 | 6 | 0.04 |
| 0015780 | nucleotide-sugar transport | 57 | 16 | 10 | 6 | 0.04 |
| 0010345 | suberin biosynthetic process | 15 | 6 | 3 | 3 | 0.04 |
| 0043242 | negative regulation of protein complex disassembly | 15 | 6 | 3 | 3 | 0.04 |
| 0090481 | pyrimidine nucleotide-sugar transmembrane transport | 31 | 10 | 6 | 4 | 0.04 |
| 0010412 | mannan metabolic process | 8 | 4 | 1 | 3 | 0.04 |
| 0046355 | mannan catabolic process | 8 | 4 | 1 | 3 | 0.04 |
| 0045926 | negative regulation of growth | 8 | 4 | 1 | 3 | 0.04 |
| 1902074 | response to salt | 8 | 4 | 1 | 3 | 0.04 |
| 2000762 | regulation of phenylpropanoid metabolic process | 8 | 4 | 1 | 3 | 0.04 |
| 0006563 | L-serine metabolic process | 44 | 13 | 8 | 5 | 0.04 |
| 0051261 | protein depolymerization | 27 | 9 | 5 | 4 | 0.04 |
| 0046185 | aldehyde catabolic process | 27 | 9 | 5 | 4 | 0.04 |
| 0031122 | cytoplasmic microtubule organization | 27 | 9 | 5 | 4 | 0.04 |
| 0009251 | glucan catabolic process | 71 | 19 | 13 | 6 | 0.04 |
| 0006000 | fructose metabolic process | 19 | 7 | 3 | 4 | 0.04 |
| 0010038 | response to metal ion | 152 | 36 | 27 | 9 | 0.04 |
| 0006641 | triglyceride metabolic process | 23 | 8 | 4 | 4 | 0.04 |
| 0034765 | regulation of ion transmembrane transport | 23 | 8 | 4 | 4 | 0.04 |
| 0044106 | cellular amine metabolic process | 62 | 17 | 11 | 6 | 0.04 |
| 0006576 | cellular biogenic amine metabolic process | 62 | 17 | 11 | 6 | 0.04 |
| 0019725 | cellular homeostasis | 323 | 70 | 57 | 13 | 0.04 |
| 0051186 | cofactor metabolic process | 556 | 115 | 99 | 16 | 0.04 |
| 0046686 | response to cadmium ion | 90 | 23 | 16 | 7 | 0.04 |
| 1901264 | carbohydrate derivative transport | 109 | 27 | 19 | 8 | 0.04 |
| 1901698 | response to nitrogen compound | 114 | 28 | 20 | 8 | 0.04 |
| 0006636 | unsaturated fatty acid biosynthetic process | 5 | 3 | 1 | 2 | 0.04 |
| 0046717 | acid secretion | 5 | 3 | 1 | 2 | 0.04 |
| 0006624 | vacuolar protein processing | 5 | 3 | 1 | 2 | 0.04 |
| 0071715 | icosanoid transport | 5 | 3 | 1 | 2 | 0.04 |
| 1903963 | arachidonate transport | 5 | 3 | 1 | 2 | 0.04 |
| 0009692 | ethylene metabolic process | 5 | 3 | 1 | 2 | 0.04 |
| 0009693 | ethylene biosynthetic process | 5 | 3 | 1 | 2 | 0.04 |
| 0009593 | detection of chemical stimulus | 5 | 3 | 1 | 2 | 0.04 |
| 0010555 | response to mannitol | 5 | 3 | 1 | 2 | 0.04 |
| 1903862 | positive regulation of oxidative phosphorylation | 5 | 3 | 1 | 2 | 0.04 |
| 0002082 | regulation of oxidative phosphorylation | 5 | 3 | 1 | 2 | 0.04 |
| 1901571 | fatty acid derivative transport | 5 | 3 | 1 | 2 | 0.04 |
| 1904659 | glucose transmembrane transport | 5 | 3 | 1 | 2 | 0.04 |
| 0048830 | adventitious root development | 5 | 3 | 1 | 2 | 0.04 |
| 0015909 | long-chain fatty acid transport | 5 | 3 | 1 | 2 | 0.04 |
| 0010206 | photosystem II repair | 5 | 3 | 1 | 2 | 0.04 |
| 0050482 | arachidonic acid secretion | 5 | 3 | 1 | 2 | 0.04 |
| 0006123 | mitochondrial electron transport, cytochrome c to oxygen | 5 | 3 | 1 | 2 | 0.04 |
| 1903578 | regulation of ATP metabolic process | 5 | 3 | 1 | 2 | 0.04 |
| 1903580 | positive regulation of ATP metabolic process | 5 | 3 | 1 | 2 | 0.04 |
| 0045979 | positive regulation of nucleoside metabolic process | 5 | 3 | 1 | 2 | 0.04 |
| 0045981 | positive regulation of nucleotide metabolic process | 5 | 3 | 1 | 2 | 0.04 |
| 0035428 | hexose transmembrane transport | 5 | 3 | 1 | 2 | 0.04 |
| 0032309 | icosanoid secretion | 5 | 3 | 1 | 2 | 0.04 |
| 0006011 | UDP-glucose metabolic process | 5 | 3 | 1 | 2 | 0.04 |
| 0043449 | cellular alkene metabolic process | 5 | 3 | 1 | 2 | 0.04 |
| 0043450 | alkene biosynthetic process | 5 | 3 | 1 | 2 | 0.04 |
| 0015790 | UDP-xylose transport | 5 | 3 | 1 | 2 | 0.04 |
| 0015758 | glucose transport | 5 | 3 | 1 | 2 | 0.04 |
| 1900673 | olefin metabolic process | 5 | 3 | 1 | 2 | 0.04 |
| 1900674 | olefin biosynthetic process | 5 | 3 | 1 | 2 | 0.04 |
| 0033559 | unsaturated fatty acid metabolic process | 5 | 3 | 1 | 2 | 0.04 |
| 1900544 | positive regulation of purine nucleotide metabolic process | 5 | 3 | 1 | 2 | 0.04 |
| 0009786 | regulation of asymmetric cell division | 5 | 3 | 1 | 2 | 0.04 |
| 0009639 | response to red or far red light | 36 | 11 | 6 | 5 | 0.04 |
| 0072531 | pyrimidine-containing compound transmembrane transport | 45 | 13 | 8 | 5 | 0.05 |
| 0019344 | cysteine biosynthetic process | 32 | 10 | 6 | 4 | 0.05 |
| 0009312 | oligosaccharide biosynthetic process | 77 | 20 | 14 | 6 | 0.05 |
| 0043649 | dicarboxylic acid catabolic process | 12 | 5 | 2 | 3 | 0.05 |
| 1904666 | regulation of ubiquitin protein ligase activity | 12 | 5 | 2 | 3 | 0.05 |
| 0043101 | purine-containing compound salvage | 12 | 5 | 2 | 3 | 0.05 |
| 0048832 | specification of plant organ number | 12 | 5 | 2 | 3 | 0.05 |
| 0048833 | specification of floral organ number | 12 | 5 | 2 | 3 | 0.05 |
| 0009900 | dehiscence | 12 | 5 | 2 | 3 | 0.05 |
| 0009901 | anther dehiscence | 12 | 5 | 2 | 3 | 0.05 |
| 0006873 | cellular ion homeostasis | 120 | 29 | 21 | 8 | 0.05 |
| 0050789 | regulation of biological process | 5189 | 963 | 921 | 42 | 0.05 |
| 0006855 | drug transmembrane transport | 101 | 25 | 18 | 7 | 0.05 |
| 0050790 | regulation of catalytic activity | 618 | 126 | 110 | 16 | 0.05 |
| 0005987 | sucrose catabolic process | 24 | 8 | 4 | 4 | 0.05 |
| 0048268 | clathrin coat assembly | 24 | 8 | 4 | 4 | 0.05 |
| 0034762 | regulation of transmembrane transport | 24 | 8 | 4 | 4 | 0.05 |
| 0090627 | plant epidermal cell differentiation | 16 | 6 | 3 | 3 | 0.05 |
